# Supplementary material for: Two New Compounds from the Fungus Xylaria nigripes
Source: Molecules. 2023 Jan 4;28(2):508. doi: 10.3390/molecules28020508 (PMC9862878; doi:10.3390/molecules28020508)
Supplement: Supplementary file 1 [file molecules-28-00508-s001.zip › molecules-2093142-supplementary.pdf]

Supporting Information for

Two new compounds from the fungus *Xylaria nigripes*

Hong-Ping Long [1], Siqian Zhou[1], Lan-Qing Li [3], Jing Li [2,4]*, Ji-Kai Liu [3]*

[1] The First Hospital of Hunan University of Chinese Medicine, Changsha, Hunan 410007, China

[2] Department of Pharmacy, Xiangya Hospital, Central South University, Changsha, Hunan 410008, China

[3] School of Pharmaceutical Sciences, South-Central Minzu University, Wuhan, Hubei 430074, China

[4] National Clinical Research Center for Geriatric Disorders, Xiangya Hospital, Central South University, Changsha, Hunan 410008, China

*Corresponding authors: lijingliyun@csu.edu.cn (J.L.), liujikai@mail.scuec.edu.cn (J.-K.L.)

Contents

Table S1. Cell viability of normal PC12 cells with different concentrations of compounds **1** and **2**. 3
Figure S1. Cell viability of normal PC12 cells with different concentrations of compounds **1** and 2. 3
Table S2. Cell viability of PC12 cells evaluated by the CCK8 test (** *p* < 0.01 compared with control group, ## *p* < 0.01 compared with model group) 4
Table S3. Cell viability of the tested compounds 1 and 2 compared with DMSO group (**p* < 0.05, ** *p* < 0.01) 4
Figure S2. The cell viability of DMSO group with different concentrations of compounds **1** and **2.** 4
Table S4. The neuronal apoptosis rate was presented as the percentage of Annexin FITC/PI-positive neurons. The percentage of Annexin FITC/PI-positive neurons was calculated by dividing the number of Annexin FITC/PI-positive neurons with the total number of neurons. Data are presented as the mean±SD. ***P* < 0.01 vs. Control group; ##*P* < 0.01 vs. Model group. 5
Figure S3. The apoptosis rate of PC12 cells with different groups.. 5
Figure S4. ^{1}H NMR spectrum of **1** (600 MHz, Methanol-d_4) 6
Figure S5. ^{13}C NMR and DEPT spectrum of **1** (150 MHz, Methanol-d_4) 6
Figure S6. ^{1}H-^{1}H COSY spectrum of **1** 7
Figure S7. HSQC spectrum of **1** 7
Figure S8. HMBC spectrum of **1** 8
Figure S9. NOESY spectrum of **1** 8
Figure S10. HR-ESI-MS spectrum of **1** 9
Figure S11. IR spectra of **1** (in MeOH) 10
Figure S12. ^{1}H NMR spectrum of **2** (600 MHz, Methanol-d_4) 10
Figure S13. ^{13}C NMR and DEPT spectrum of **2** (150 MHz, Methanol-d_4) 11
Figure S14. ^{1}H-^{1}H COSY spectrum of **2** 11
Figure S15. HSQC spectrum of **2** 12
Figure S16. HMBC spectrum of **2** 12
Figure S17. NOESY spectrum of **2** 13
Figure S18. HR-ESI-MS spectrum of **2** 13
Figure S19. IR spectra of **2** (in MeOH) 14
Table S5. Geometry data of conformers of structure **1a** 14
Table S6. Geometry data of conformers of structure **1b** 115

1. Cytotoxicity assay

The cytotoxicity experiments were grouped as follows: normal group, 0.1 μmol·L^{-1} Nimodipine group), compound **1** group (0.01, 0.1, 1 and 10 μmol·L^{-1}), compound **2** group (0.01, 0.1, 1 and 10 μmol·L^{-1}). Cells were digested with 1×10^4 cells per well and 100 μL per well. After application, culture media and different concentrations of drug solution were added, and the media was discarded for 24 h, and each group was respectively Medium with 10% CCK8 reagent was added to continue the light-evasive incubation for 3 h, and the absorbance values were measured at 450 nm.

Table S1. Cell viability of normal PC12 cells with different concentrations of compounds **1** and **2**.

Compounds	Drug concentration (μM)	Mean±SD
Control	-	100.00±2.21
Nimodipine	10.00	99.13±1.11
	10.00	95.15±4.84
Compound **1**	1.00	108.12±6.17
	0.10	95.15±4.84
	0.01	111.42±3.28
	10.00	107.51±1.57
Compound **2**	1.00	104.97±1.40
	0.10	105.36±0.92
	0.01	102.28±7.52

Figure S1. Cell viability of normal PC12 cells with different concentrations of compounds **1** and **2**.

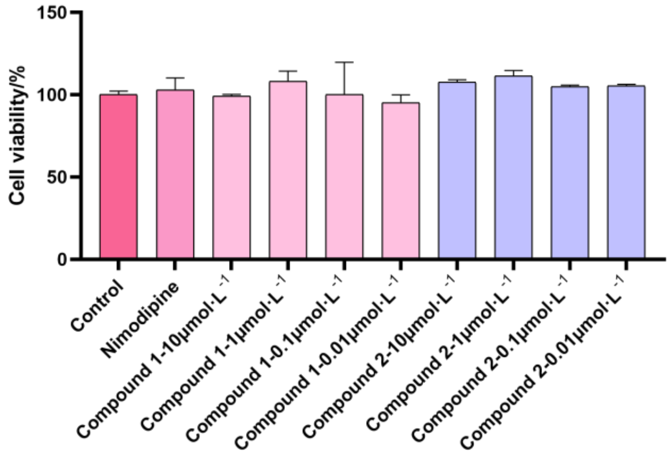

Table S2. Cell viability of PC12 cells evaluated by the CCK8 test (** $p < 0.01$ compared with control group, ## $p < 0.01$ compared with model group)

Compounds	Drug concentration (μM)	Mean±SD
Control	-	100.00±0.53
Model	-	46.75±1.98**
DMSO	-	58.95±3.33**
Nimodipine	10.00	80.06±1.70##
	10.00	65.06±2.88##
Compound **1**	1.00	72.52±4.32##
	0.10	64.67±3.48##
	0.01	61.72±2.63##
	10.00	67.20±3.59##
Compound **2**	1.00	79.72±1.24##
	0.10	73.11±0.67##
	0.01	64.19±5.73##

Table S3. Cell viability of the tested compounds **1** and **2** compared with DMSO group (*$p < 0.05$, ** $p < 0.01$)

**Compounds**	**Drug concentration (μM)**	**Mean±SD**
DMSO	-	58.95±3.33
	10.00	65.07±2.88*
Compound **1**	10.00	72.53±4.32**
	1.00	64.67±3.48
	0.10	61.72±2.63
	0.01	67.20±3.59**
Compound **2**	10.00	79.72±1.24**
	1.00	73.11±0.67**
	0.10	64.20±5.73

Figure S2. The cell viability of DMSO group with different concentrations of compounds **1** and **2**.

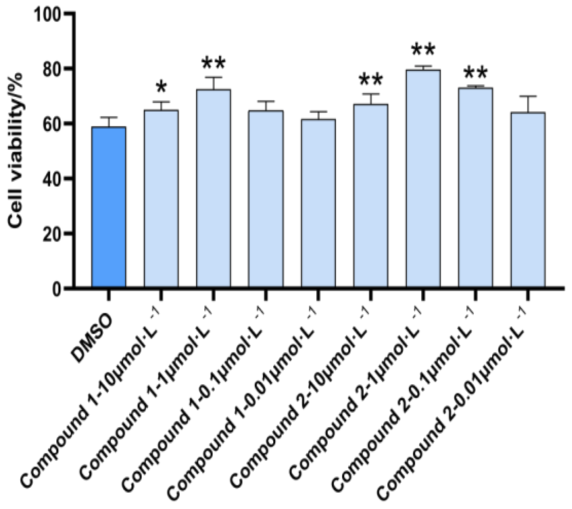

Table S4. The neuronal apoptosis rate was presented as the percentage of Annexin FITC/PI-positive neurons. The percentage of Annexin FITC/PI-positive neurons was calculated by dividing the number of Annexin FITC/PI-positive neurons with the total number of neurons. Data are presented as the mean±SD. $^{**}P < 0.01$ vs. Control group; $^{\#\#}P < 0.01$ vs. Model group.

**Compounds**	**Drug concentration (*μ*M)**	**Mean±SD**
Control	-	3.15±1.62
Model	-	17.43±2.43**
DMSO	-	8.09±3.32**
Nimodipine	10.00	7.04±0.73$^{\#\#}$
Compound **1**	1.00	10.00±1.83$^{\#\#}$
Compound **2**	1.00	3.95±1.95$^{\#\#}$

Figure S3. The apoptosis rate of PC12 cells with different groups..

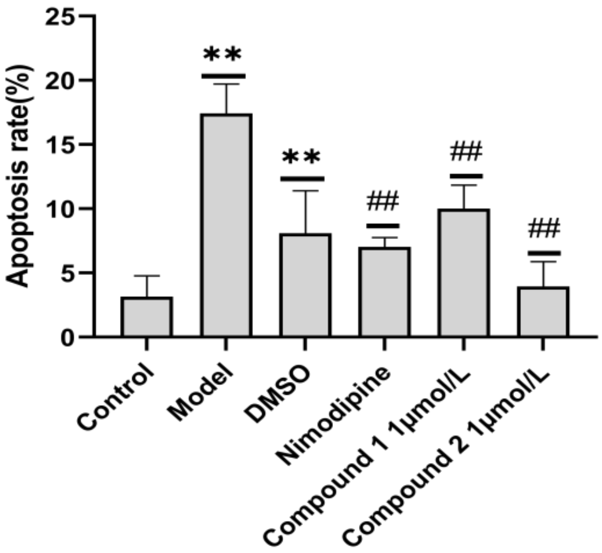

Figure S4. ^{1}H NMR spectrum of **1** (600 MHz, Methanol-d_4)

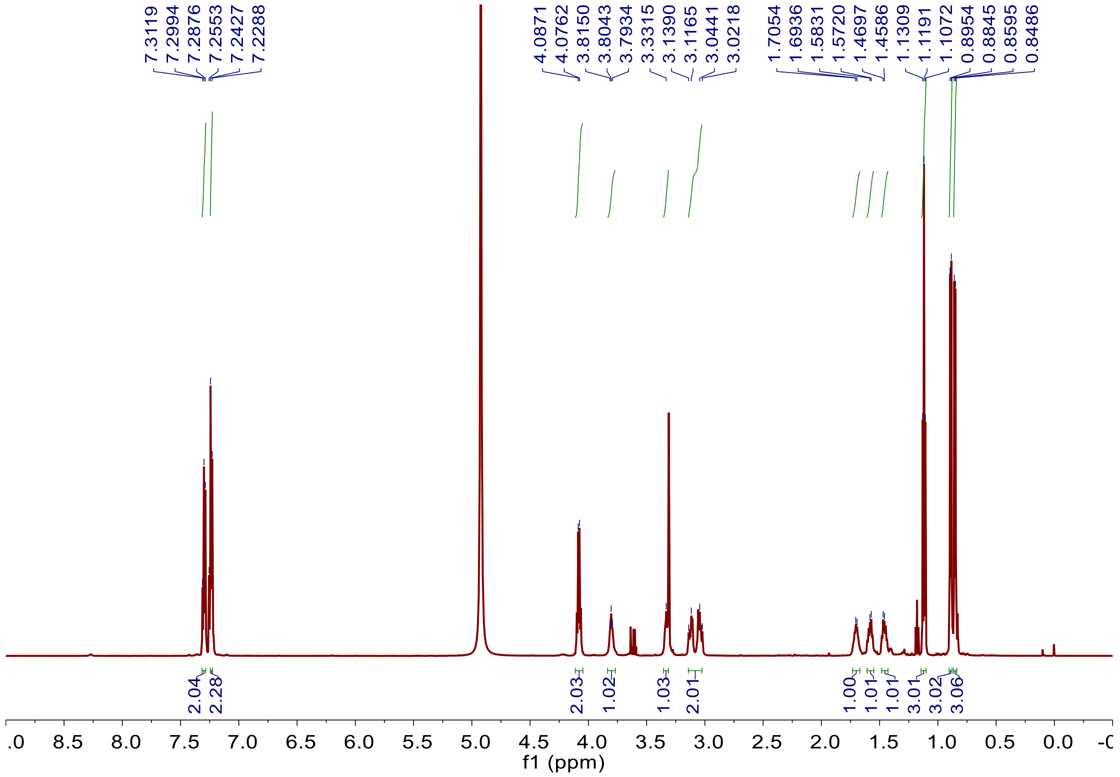

Figure S5. ^{13}C NMR and DEPT spectrum of **1** (150 MHz, Methanol-d_4)

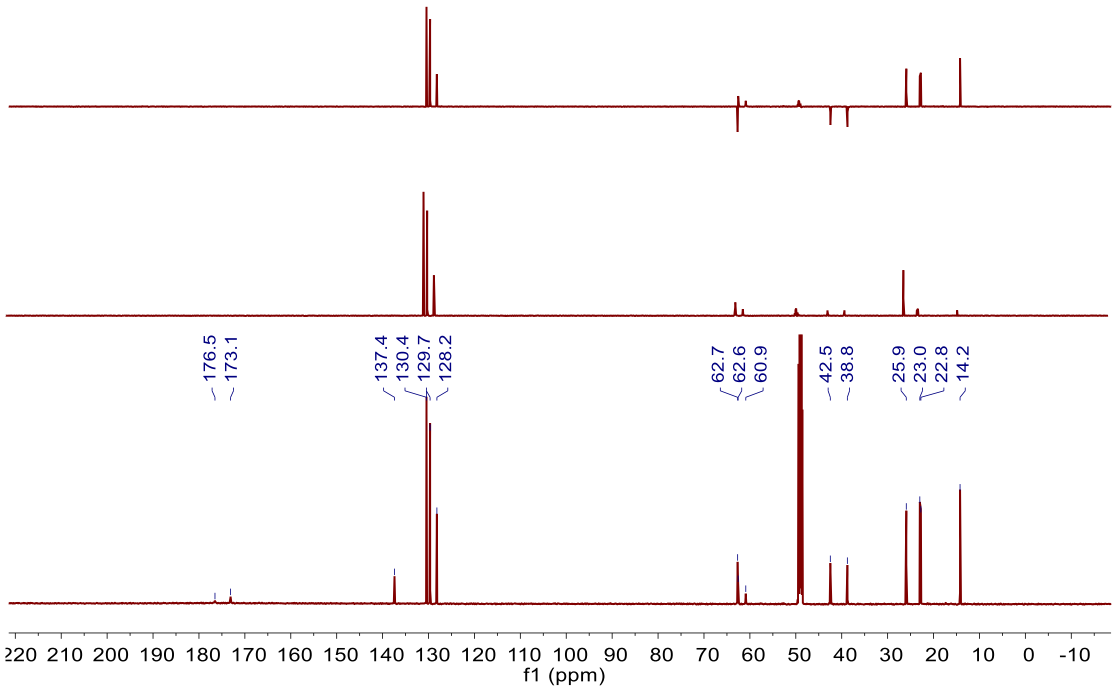

Figure S6. ^{1}H-^{1}H COSY spectrum of **1**

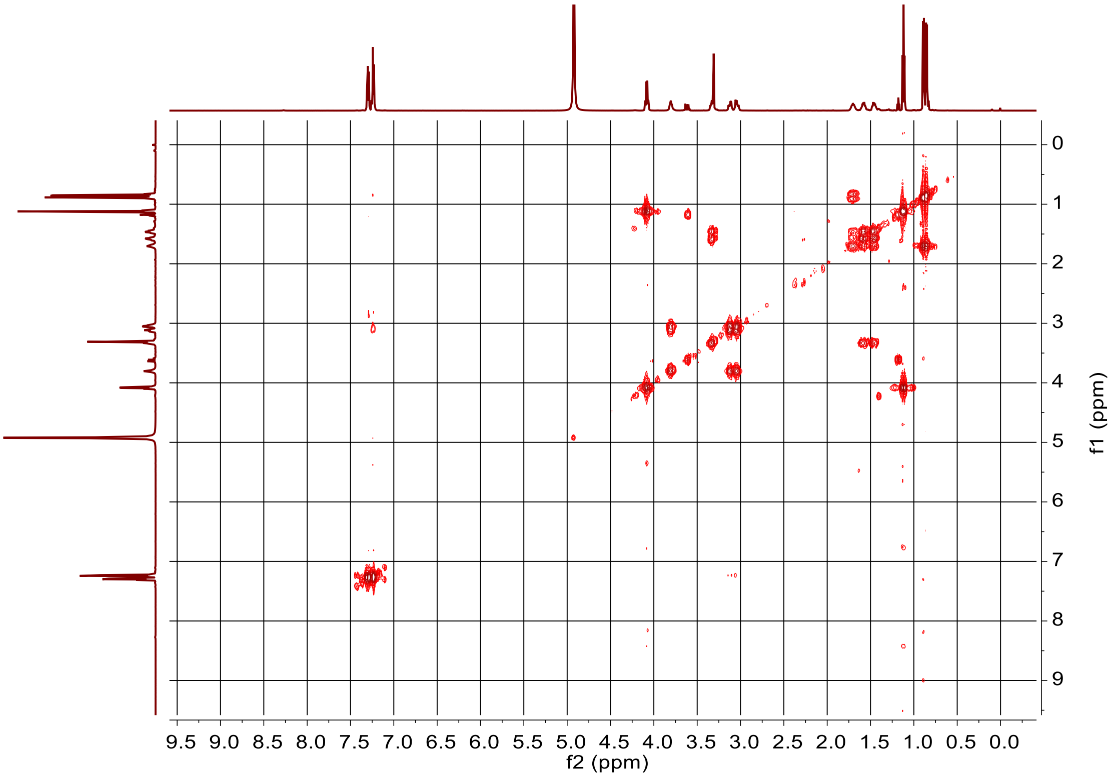

Figure S7. HSQC spectrum of **1**

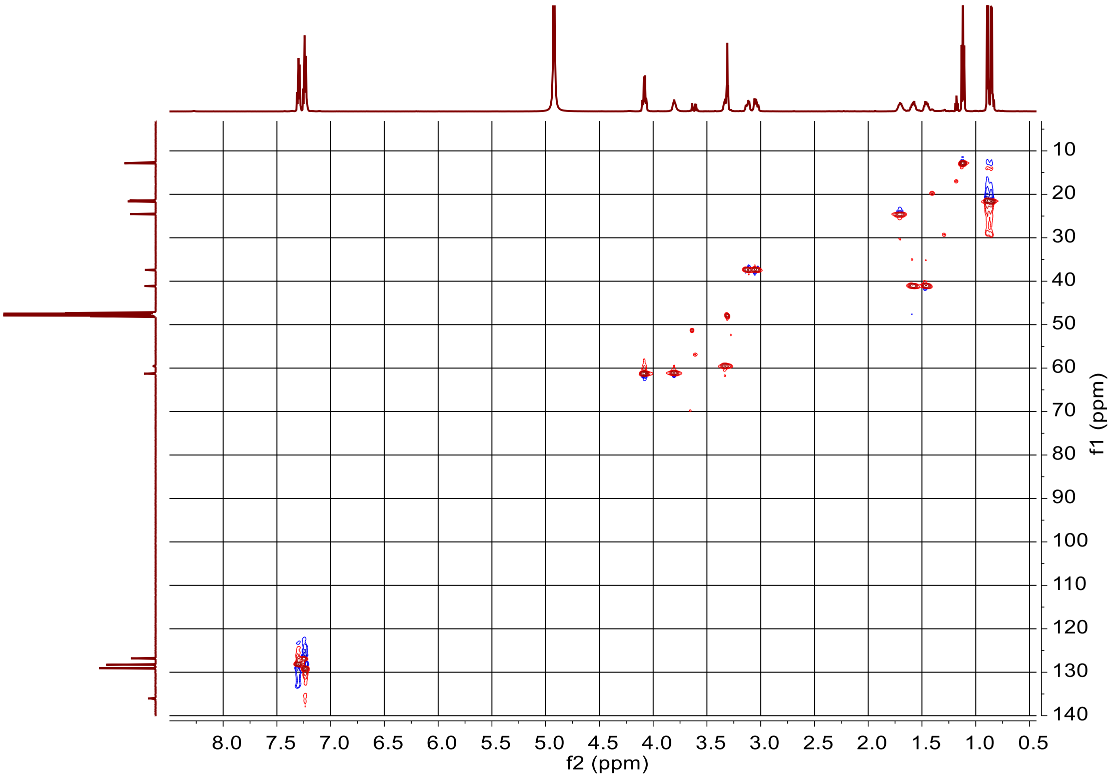

Figure S8. HMBC spectrum of **1**

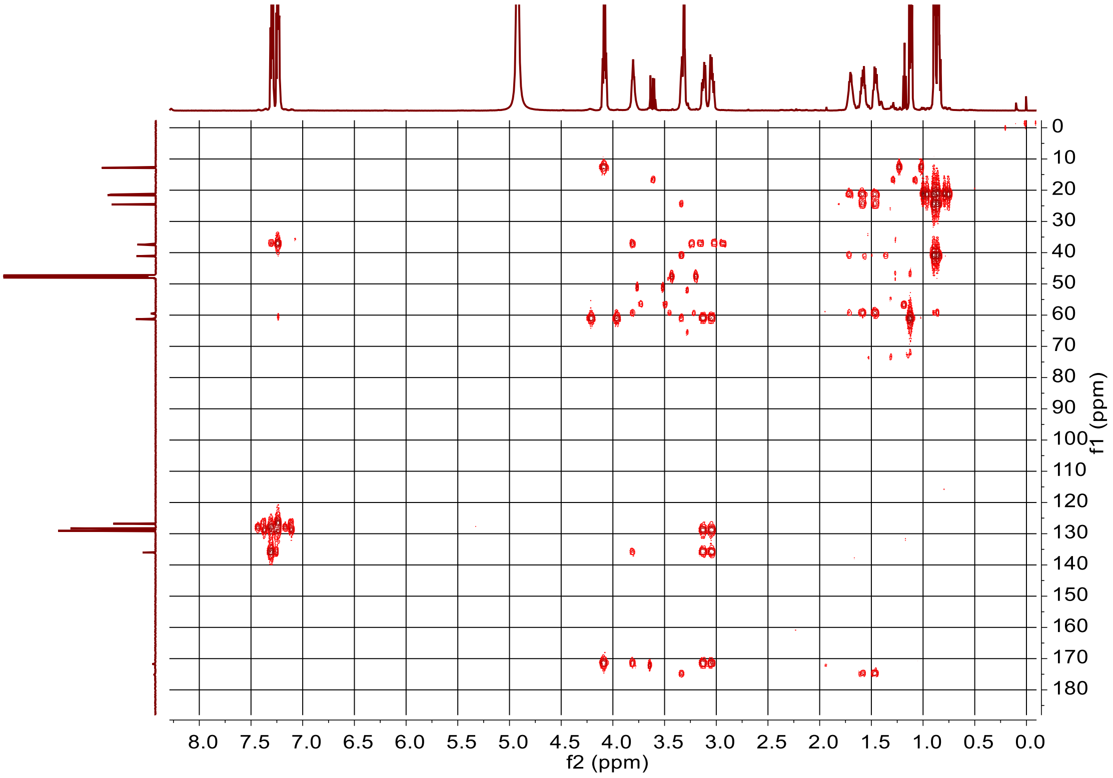

Figure S9. NOESY spectrum of **1**

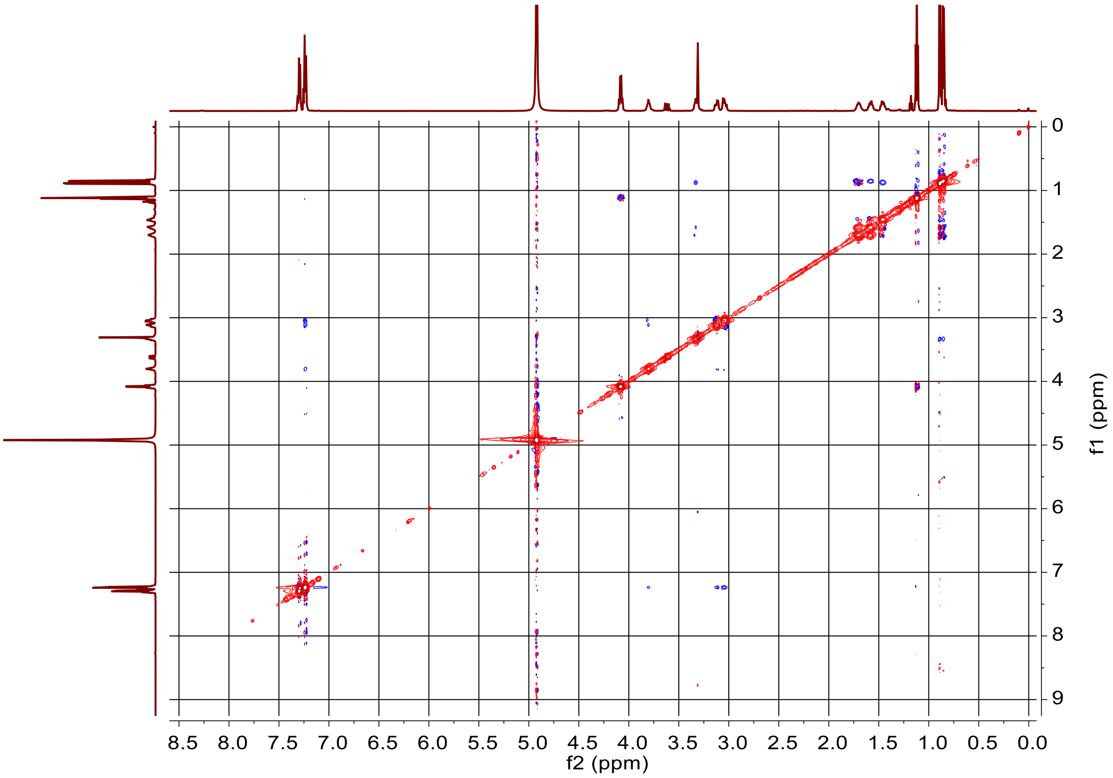

Figure S10. HR-ESI-MS spectrum of **1**

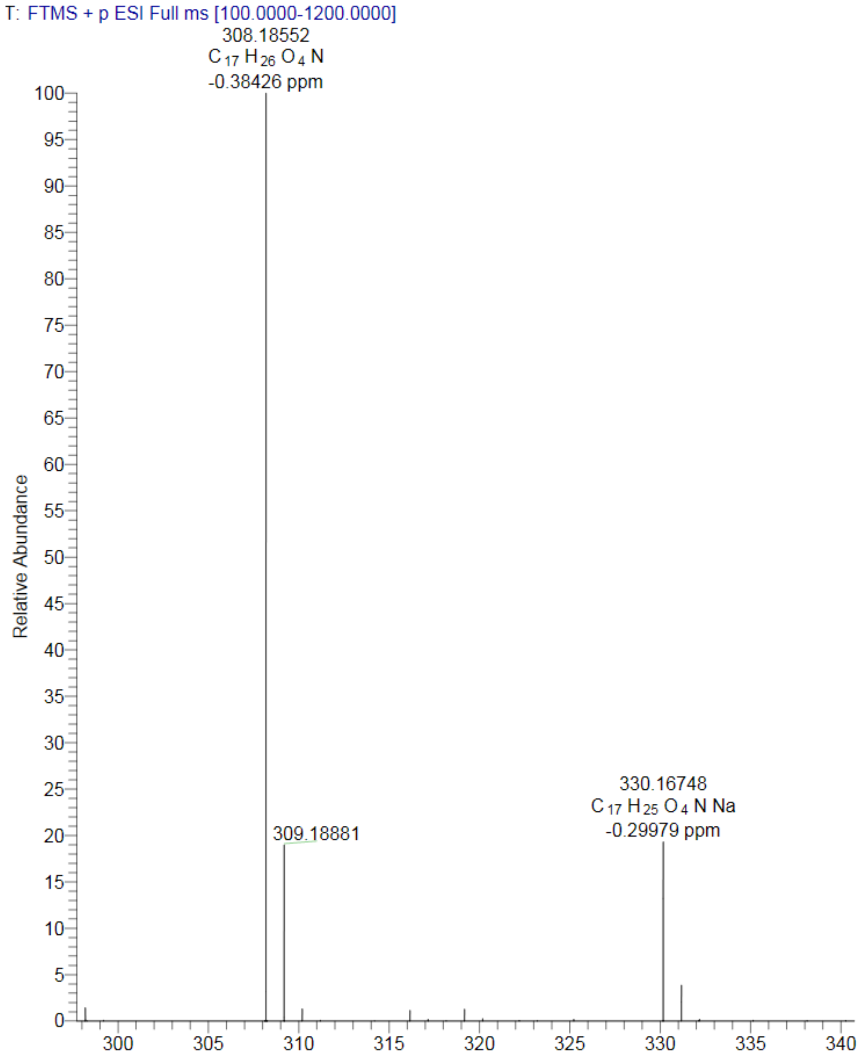

Figure S11. IR spectra of **1** (in MeOH)

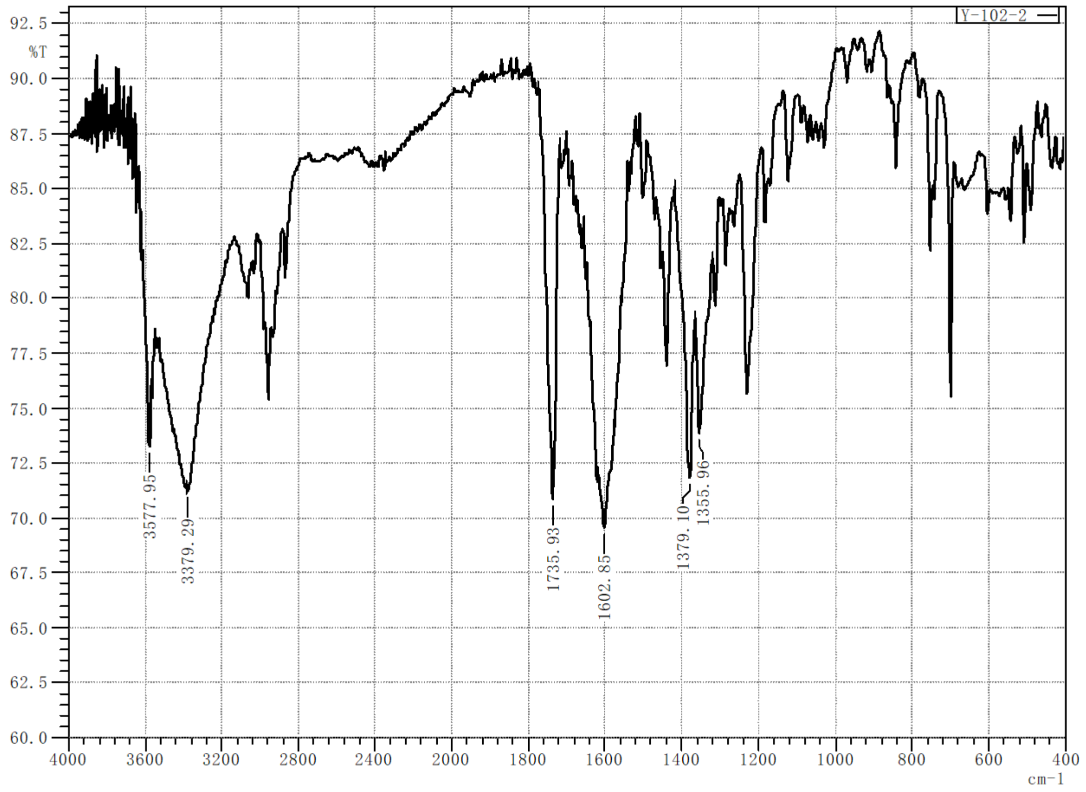

Figure S12. ^{1}H NMR spectrum of **2** (600 MHz, Methanol-d_4)

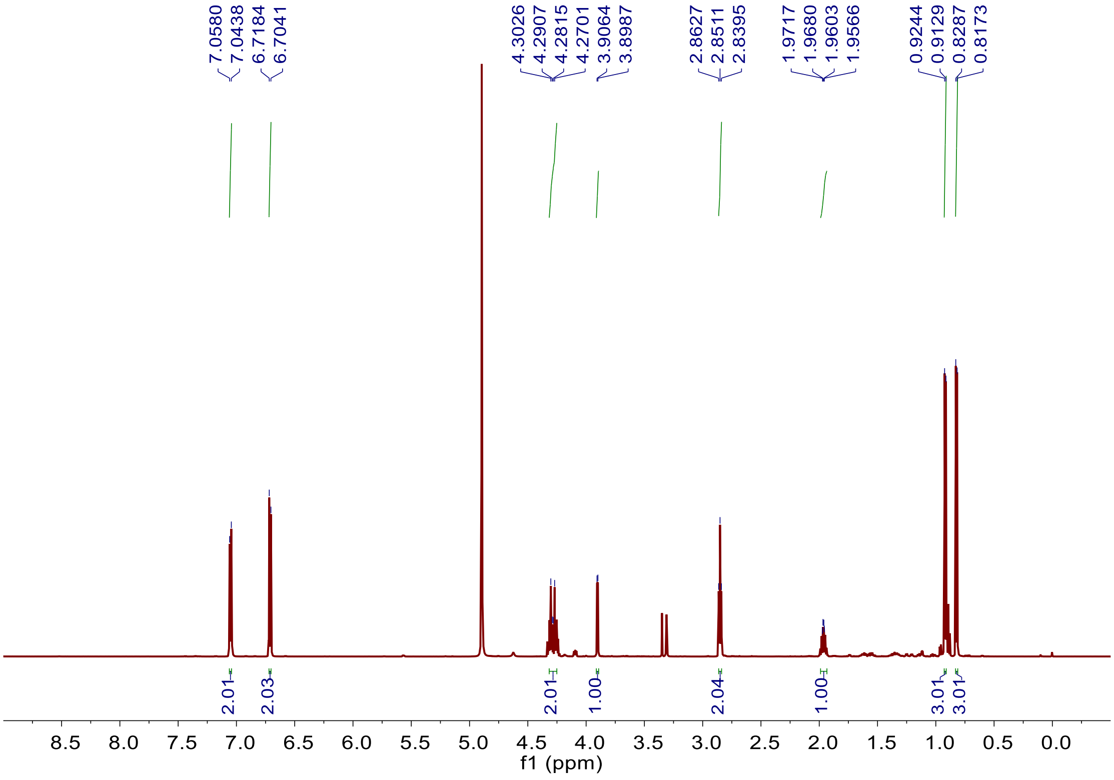

Figure S13. ^{13}C NMR and DEPT spectrum of **2** (150 MHz, Methanol-d_4)

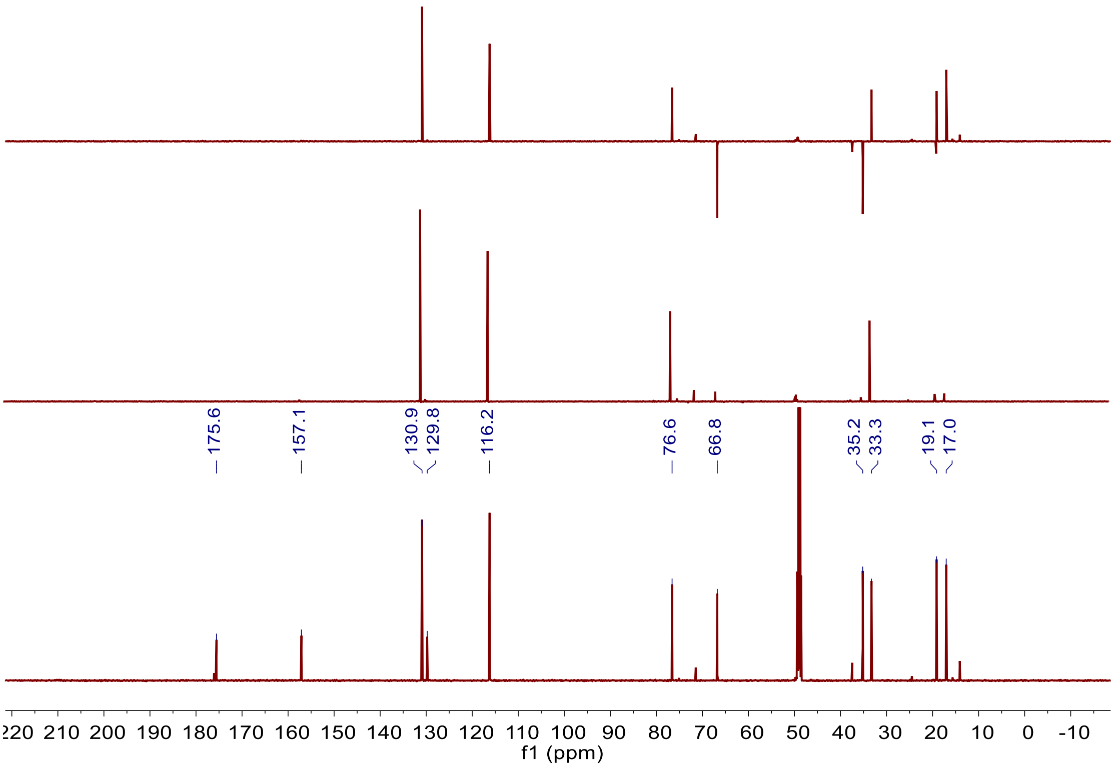

Figure S14. ^{1}H-^{1}H COSY spectrum of **2**

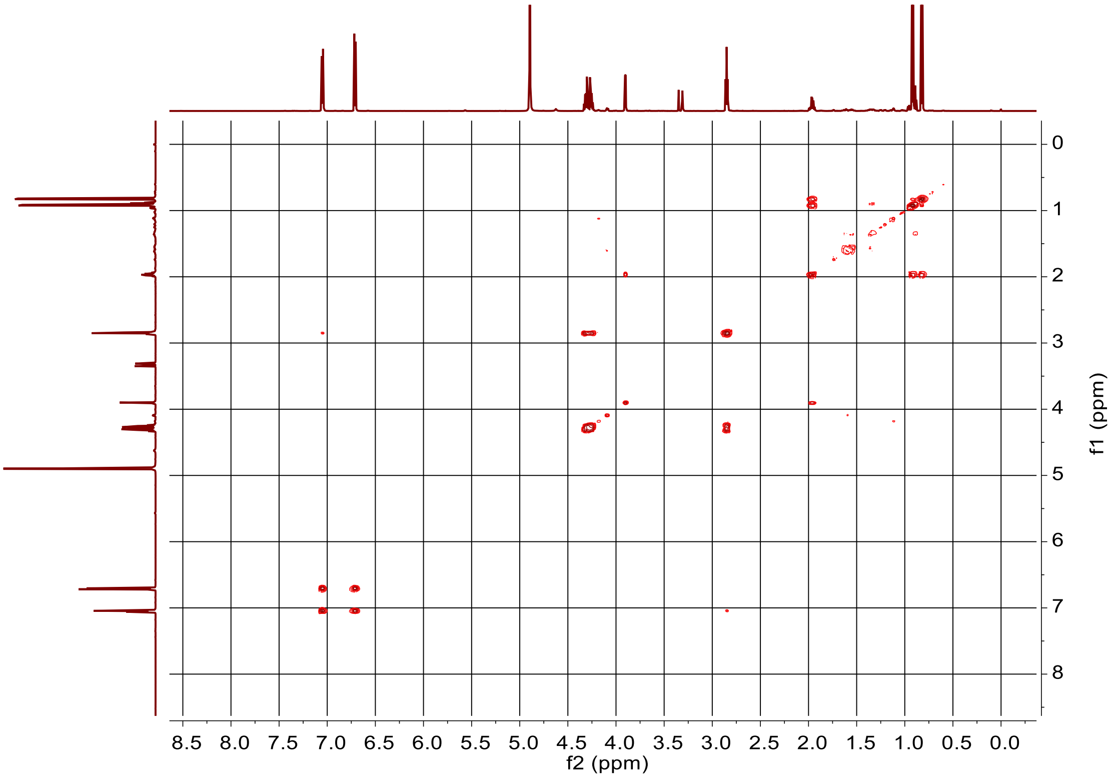

Figure S15. HSQC spectrum of **2**

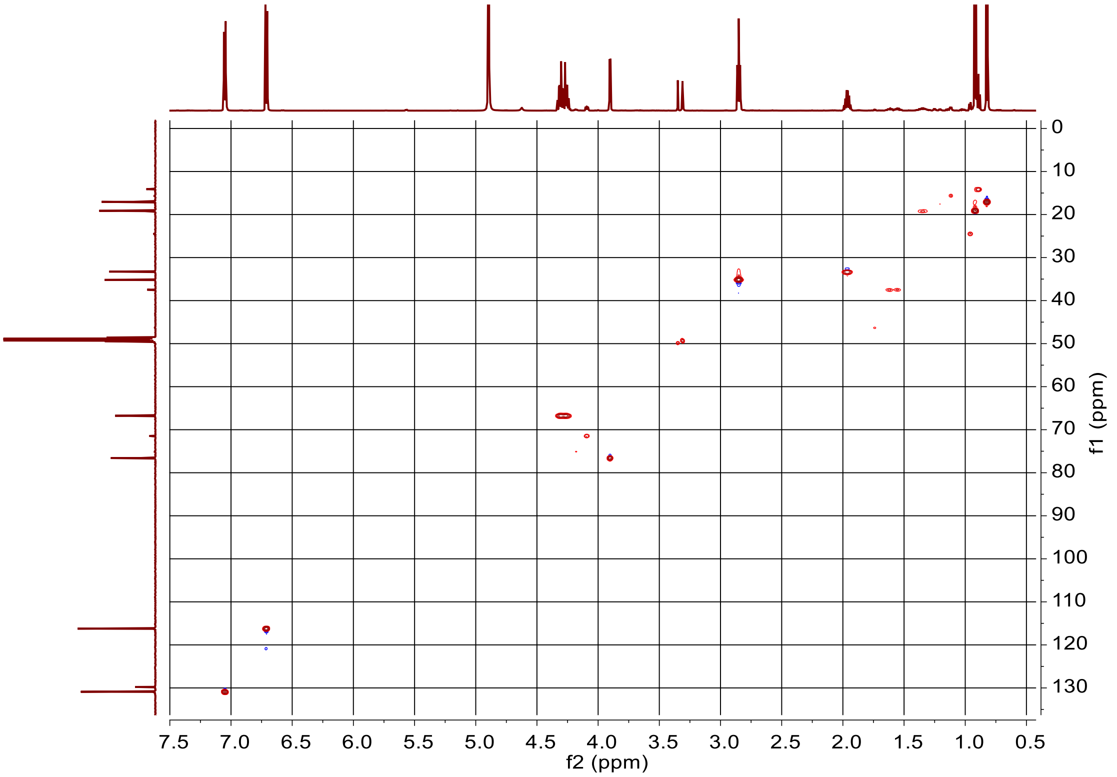

Figure S16. HMBC spectrum of **2**

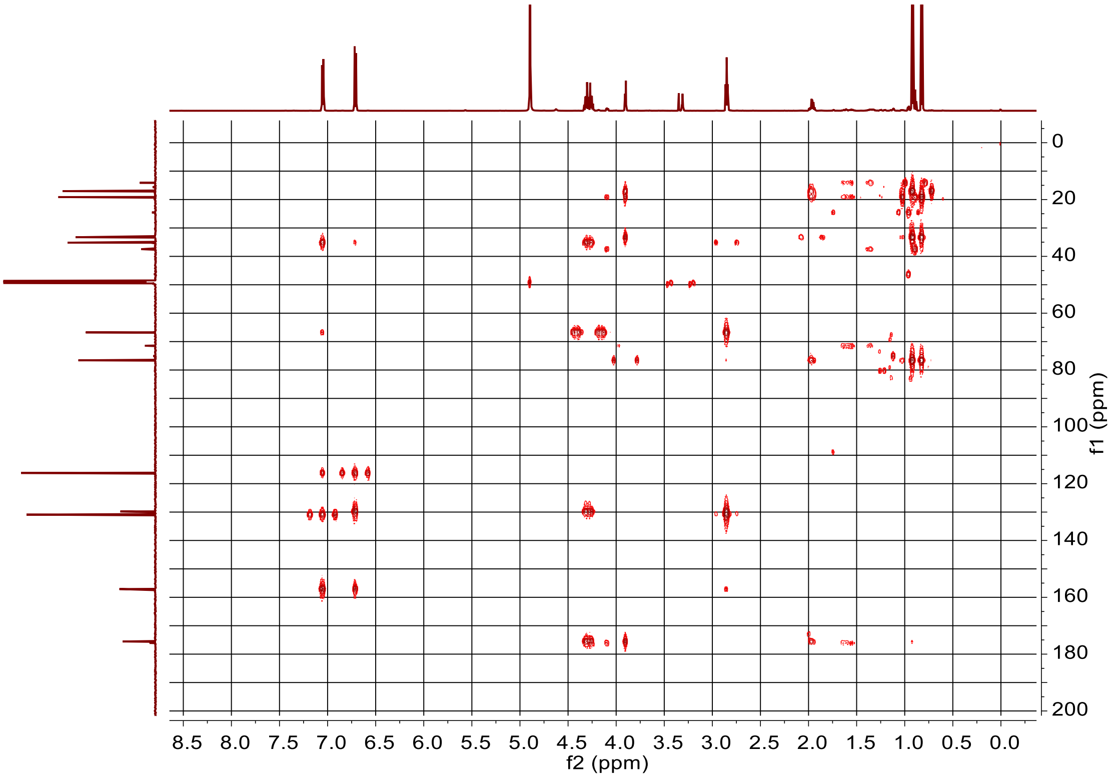

Figure S17. NOESY spectrum of **2**

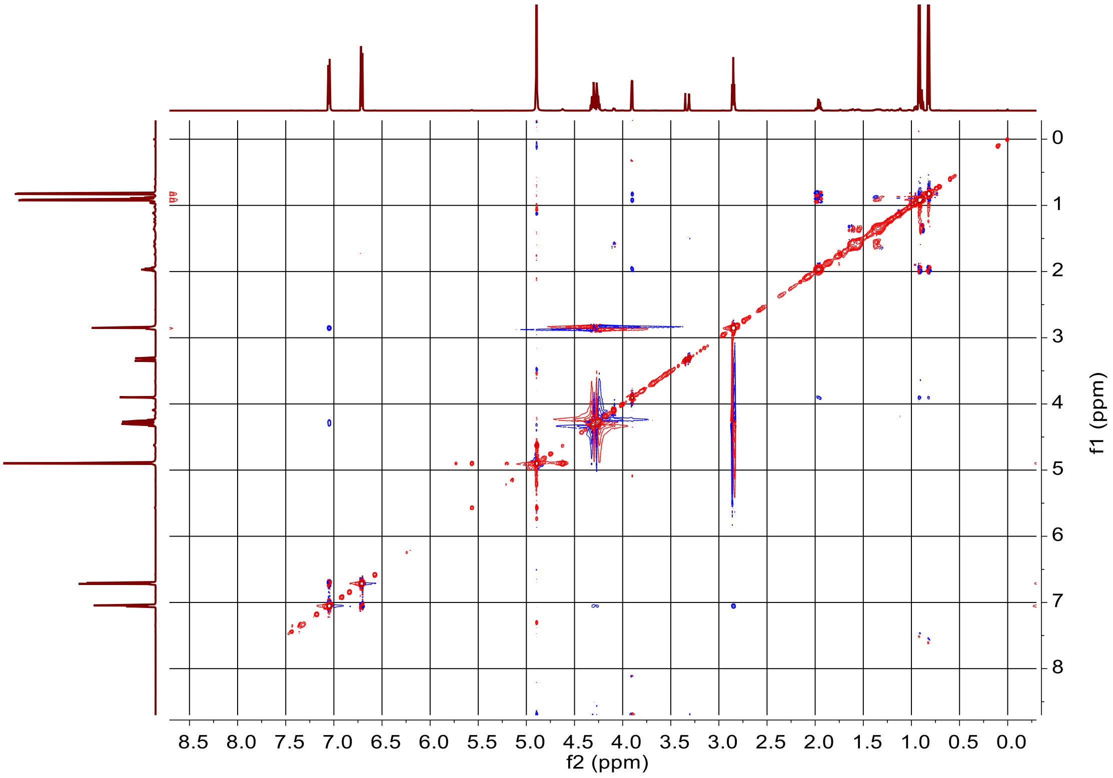

Figure S18. HR-ESI-MS spectrum of **2**

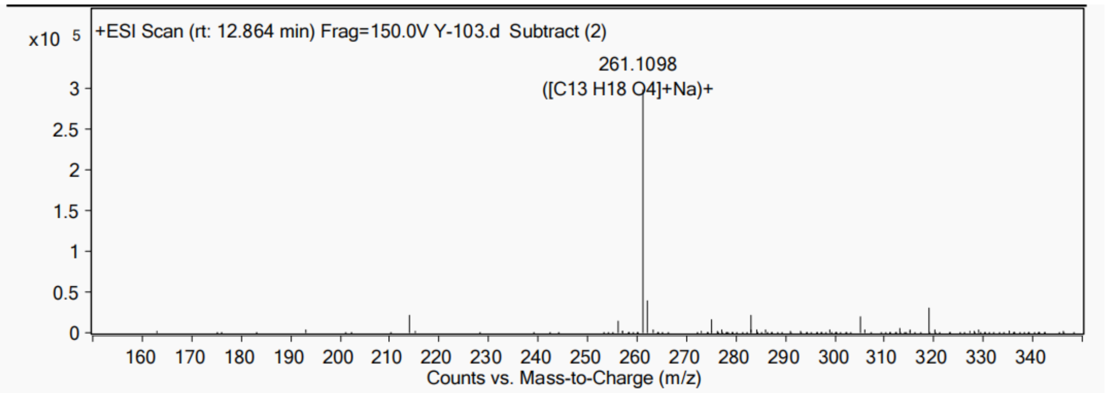

Peak List

m/z	Abund	Formula	Ion
121.0643	120987.83		
261.1098	299228	C13 H18 O4	(M+Na)+
497.2134	76132.87		

Figure S19. IR spectra of **2** (in MeOH)

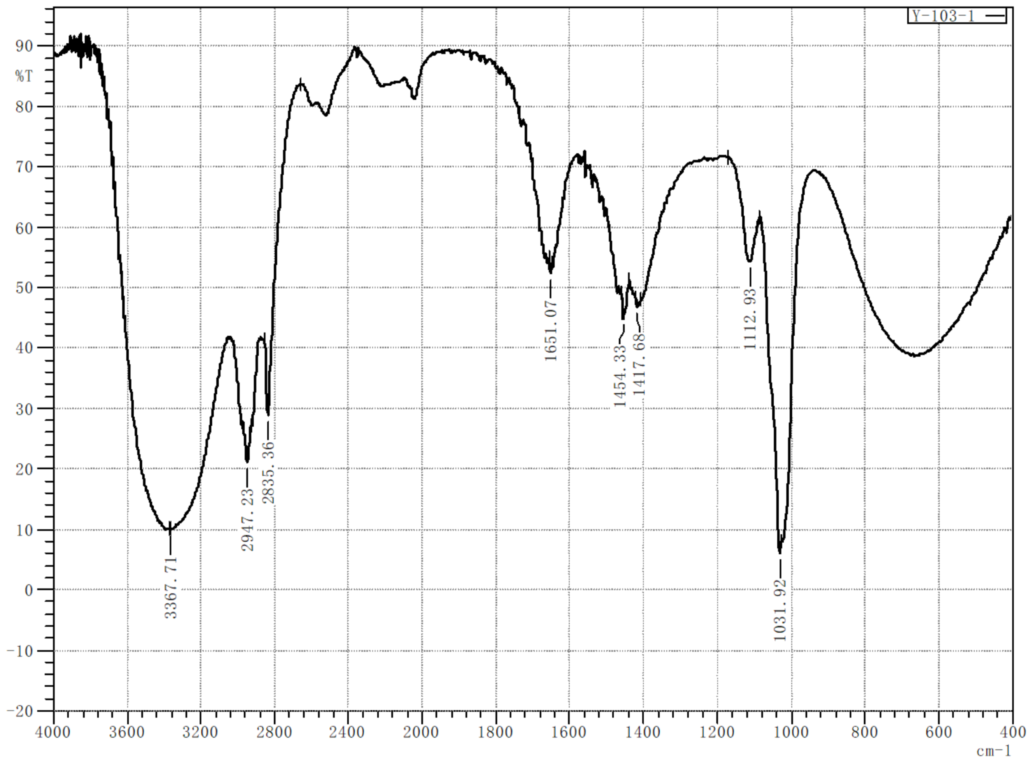

Table S5. Geometry data of conformers of structure **1a**

1a-C25, ΔG = 0.0000 kcal/mol, population = 37.75 %

C 14.877511 -3.017902 9.406069
C 13.859154 -3.239045 8.486134
C 12.729381 -3.964350 8.856815
C 12.599865 -4.469487 10.151179
C 13.635720 -4.252332 11.063295
C 14.765846 -3.533675 10.695249
C 11.337054 -5.164178 10.590216
C 10.442926 -4.228091 11.437686
C 9.257386 -5.039893 11.952855
O 9.600563 -5.752149 13.027082
O 8.162292 -5.065348 11.437244
C 8.593763 -6.644502 13.585475
C 9.219144 -7.355506 14.761014
C 12.093497 -0.874423 11.172325
C 12.399552 -0.916688 12.670086
C 12.894741 0.233881 10.486436
C 10.598130 -0.686953 10.886262
C 9.702397 -1.844136 11.351026
N 9.985027 -3.097779 10.646838
C 8.225042 -1.458156 11.170379
O 7.740595 -0.447291 11.630156

O 7.505788 -2.325932 10.462384
H 15.753892 -2.449677 9.121817
H 13.939982 -2.846485 7.480405
H 11.940895 -4.136625 8.133125
H 13.549094 -4.639293 12.071875
H 15.557654 -3.369663 11.415230
H 10.765467 -5.501896 9.723627
H 11.574697 -6.040497 11.193306
H 11.021099 -3.906726 12.309413
H 7.732495 -6.044463 13.879301
H 8.281186 -7.338526 12.805199
H 9.535340 -6.644366 15.525183
H 8.486823 -8.032119 15.204483
H 10.083135 -7.943141 14.448023
H 12.413531 -1.822643 10.737557
H 12.087551 0.012235 13.155973
H 11.895782 -1.743137 13.174649
H 13.471253 -1.041566 12.836287
H 12.618431 1.217071 10.878423
H 12.717701 0.238472 9.408658
H 13.965113 0.094143 10.649854
H 10.244369 0.230032 11.361457
H 10.450524 -0.558451 9.808399
H 9.832104 -1.992610 12.426250
H 8.120791 -3.078986 10.264125
H 10.607359 -2.933330 9.867289

1a-C82, ΔG = 0.3527 kcal/mol, population = 20.80 %
C 14.014037 -2.922826 7.953976
C 14.257989 -3.252905 9.285289
C 13.354111 -4.041798 9.985024
C 12.193154 -4.514804 9.368430
C 11.965657 -4.192002 8.030109
C 12.868185 -3.397910 7.326628
C 11.177721 -5.296075 10.161238
C 10.377048 -4.380492 11.116590
C 9.467206 -5.264275 11.966051
O 10.138179 -5.783014 12.996920
O 8.306126 -5.496005 11.713802
C 9.425753 -6.699655 13.879332
C 8.649446 -5.943580 14.936850
C 11.377725 -0.885338 10.000420
C 12.119066 -0.724742 11.327949
C 11.749297 0.238899 9.031397

C 9.855043 -0.929551 10.181274
C 9.333730 -2.114639 11.007079
N 9.596468 -3.407231 10.369405
C 7.826088 -1.939008 11.250267
O 7.347265 -0.955273 11.770820
O 7.072091 -2.955130 10.836853
H 14.714381 -2.301297 7.410856
H 15.150267 -2.889425 9.779168
H 13.544437 -4.285074 11.023819
H 11.075809 -4.562099 7.534045
H 12.672869 -3.150241 6.290943
H 10.477622 -5.800612 9.492577
H 11.670946 -6.060444 10.762119
H 11.086424 -3.895305 11.793664
H 8.776319 -7.329279 13.273658
H 10.213580 -7.308385 14.316594
H 7.864987 -5.334312 14.488297
H 8.182066 -6.656372 15.619010
H 9.311480 -5.298952 15.516558
H 11.701522 -1.824040 9.547931
H 11.814073 0.194619 11.836011
H 11.937160 -1.558933 12.007583
H 13.196209 -0.676915 11.158188
H 12.826560 0.255971 8.854830
H 11.457195 1.213487 9.433045
H 11.252500 0.108911 8.067346
H 9.515396 -0.009083 10.660143
H 9.375934 -0.965993 9.197002
H 9.793974 -2.092839 11.998456
H 7.707873 -3.645492 10.515736
H 9.981001 -3.276642 9.443834

1a-C5, ΔG = 0.5221 kcal/mol, population = 15.62 %
C 14.825601 -3.094033 9.334830
C 14.758712 -3.535254 10.654464
C 13.640848 -4.227249 11.102556
C 12.572374 -4.490737 10.241968
C 12.656737 -4.060338 8.917180
C 13.774472 -3.362560 8.465787
C 11.324880 -5.155034 10.763599
C 10.469987 -4.172851 11.599679
C 9.301756 -4.954326 12.194499
O 9.685480 -5.611080 13.292225
O 8.190675 -5.000511 11.716702

C 8.725536 -6.513815 13.915459
C 8.735882 -7.868035 13.238158
C 12.128751 -0.830688 11.051770
C 12.526525 -0.842594 12.527996
C 12.877895 0.269037 10.296641
C 10.617980 -0.656184 10.852740
C 9.752603 -1.785634 11.429704
N 9.990611 -3.075147 10.775521
C 8.268598 -1.400685 11.315812
O 7.812176 -0.371764 11.763966
O 7.510374 -2.289438 10.677648
H 15.692516 -2.546565 8.987724
H 15.575778 -3.333654 11.335576
H 13.590134 -4.556786 12.133706
H 11.842676 -4.270331 8.232951
H 13.820215 -3.028159 7.437095
H 10.717888 -5.530686 9.937625
H 11.581738 -6.002652 11.398981
H 11.083433 -3.817662 12.433122
H 9.058310 -6.578067 14.948608
H 7.741304 -6.050306 13.880616
H 8.062624 -8.542957 13.770031
H 8.398292 -7.793883 12.204327
H 9.737195 -8.300442 13.253655
H 12.428365 -1.785256 10.616372
H 13.608598 -0.944426 12.629481
H 12.225407 0.086651 13.020265
H 12.072571 -1.671674 13.073438
H 12.626667 0.258869 9.233691
H 13.957366 0.133804 10.388063
H 12.626044 1.256610 10.693958
H 10.293844 0.283244 11.304887
H 10.402481 -0.578582 9.781662
H 9.947347 -1.876560 12.501544
H 8.111215 -3.052725 10.475150
H 10.580981 -2.957066 9.963313

1a-C41, ΔG = 1.1734 kcal/mol, population = 5.20 %
C 14.904537 -3.085580 9.530146
C 14.745785 -3.513140 10.846721
C 13.621257 -4.241232 11.213451
C 12.635365 -4.553584 10.273110
C 12.808469 -4.128736 8.955536
C 13.935630 -3.398978 8.584380

C 11.372542 -5.262217 10.695692
C 10.493111 -4.345862 11.575458
C 9.224927 -5.089527 11.984777
O 9.496738 -6.009303 12.913360
O 8.124715 -4.890191 11.522173
C 8.393391 -6.843260 13.368729
C 8.938382 -7.787973 14.412408
C 11.962213 -0.697989 10.744501
C 13.138023 0.168959 11.199812
C 11.191164 -0.017076 9.612098
C 11.084681 -1.046670 11.955846
C 9.872915 -1.951432 11.683735
N 10.144783 -3.131393 10.857309
C 8.657255 -1.208368 11.107189
O 7.963039 -1.926272 10.222569
O 8.311476 -0.101878 11.453375
H 15.777674 -2.512120 9.246393
H 15.497788 -3.274654 11.588246
H 13.502964 -4.563816 12.241340
H 12.052450 -4.364407 8.215771
H 14.050480 -3.072116 7.558660
H 10.801614 -5.565824 9.816260
H 11.604117 -6.160053 11.268538
H 11.051325 -4.128832 12.492632
H 7.616446 -6.192530 13.770020
H 7.987990 -7.374150 12.507144
H 9.344410 -7.238968 15.262969
H 8.133514 -8.431028 14.772259
H 9.722599 -8.421205 13.995576
H 12.392614 -1.624099 10.353437
H 13.812903 0.374361 10.366470
H 12.785975 1.126623 11.593495
H 13.713127 -0.329139 11.983255
H 10.722763 0.907100 9.958124
H 10.405016 -0.653446 9.200248
H 11.866058 0.230517 8.790325
H 11.711039 -1.546217 12.699028
H 10.716191 -0.129217 12.420346
H 9.507531 -2.305884 12.652654
H 8.466978 -2.780714 10.146435
H 10.861479 -2.921729 10.174149

1a-C1, ΔG = 1.7382 kcal/mol, population = 2.00 %
C 14.845109 -2.827374 9.044022

C 13.769581 -3.230450 8.261037
C 12.786235 -4.057447 8.798645
C 12.860951 -4.484794 10.125168
C 13.953170 -4.083436 10.898759
C 14.937898 -3.262715 10.364030
C 11.745915 -5.287046 10.744532
C 10.843523 -4.392720 11.626280
C 9.855991 -5.288502 12.368502
O 8.733295 -5.500299 11.679171
O 10.105447 -5.782938 13.445743
C 7.702609 -6.330560 12.290411
C 6.826915 -5.509204 13.213309
C 12.020762 -0.874733 10.999854
C 12.498050 -0.831860 12.451823
C 12.583048 0.308094 10.208765
C 10.490609 -0.891051 10.887547
C 9.809499 -2.126417 11.495334
N 10.192139 -3.369675 10.818212
C 8.284427 -1.947068 11.434290
O 7.699984 -1.021785 11.952806
O 7.646452 -2.893788 10.748329
H 15.607726 -2.180284 8.630019
H 13.691861 -2.901299 7.232582
H 11.952076 -4.370351 8.181176
H 14.025298 -4.407574 11.930353
H 15.775471 -2.956860 10.978122
H 11.132896 -5.752603 9.971006
H 12.150444 -6.080980 11.373111
H 11.469924 -3.949403 12.405938
H 7.142089 -6.717496 11.442824
H 8.185624 -7.154466 12.812417
H 6.020981 -6.136668 13.598598
H 7.397596 -5.127880 14.060208
H 6.385635 -4.668850 12.677076
H 12.414323 -1.785165 10.543636
H 13.588453 -0.802012 12.493523
H 12.115151 0.057810 12.960084
H 12.176573 -1.705711 13.021036
H 12.280840 0.260687 9.160256
H 13.674234 0.312030 10.244144
H 12.228403 1.257504 10.620258
H 10.077548 -0.003264 11.370537
H 10.206870 -0.834174 9.831194
H 10.057860 -2.192033 12.557588

H 8.352926 -3.544327 10.495581
H 10.749767 -3.166860 9.998840

1a-C97, ΔG = 1.8775 kcal/mol, population = 1.58 %
C 14.382287 -6.844617 10.335017
C 13.169295 -7.144695 9.723806
C 12.300552 -6.121032 9.358076
C 12.627953 -4.786931 9.601581
C 13.851986 -4.495751 10.206363
C 14.723563 -5.515260 10.571272
C 11.649568 -3.687269 9.281889
C 10.875493 -3.187630 10.523085
C 10.176513 -4.378730 11.170616
O 10.926646 -4.898336 12.141009
O 9.110037 -4.824675 10.810793
C 10.518837 -6.180119 12.696326
C 11.687762 -6.715372 13.487449
C 11.241398 0.259240 11.516267
C 11.568065 -0.168053 12.949108
C 11.640528 1.719065 11.286118
C 9.756140 0.071266 11.179805
C 9.280633 -1.384835 11.166630
N 9.893316 -2.196403 10.107576
C 7.749185 -1.456863 11.034456
O 6.986446 -0.808832 11.715907
O 7.323851 -2.314450 10.109249
H 15.059101 -7.639418 10.622019
H 12.897911 -8.175197 9.531568
H 11.354093 -6.361012 8.888043
H 14.121724 -3.463593 10.398096
H 15.669059 -5.273380 11.040554
H 12.171854 -2.826242 8.858144
H 10.920262 -4.028804 8.545646
H 11.594319 -2.790207 11.244848
H 9.635705 -6.018678 13.315038
H 10.249438 -6.838591 11.871218
H 11.422928 -7.682806 13.917325
H 12.555811 -6.849249 12.840922
H 11.953845 -6.039524 14.301267
H 11.834687 -0.355896 10.830582
H 11.348515 -1.221040 13.133526
H 12.627551 -0.014828 13.163263
H 10.990939 0.422699 13.665920
H 11.071502 2.382501 11.943515

H 11.450143 2.023638 10.254744
H 12.701485 1.871141 11.494156
H 9.147889 0.624809 11.898024
H 9.548985 0.503857 10.195304
H 9.500203 -1.844547 12.133627
H 8.158251 -2.710231 9.737888
H 10.275737 -1.606520 9.378624

1a-C18, ΔG = 1.8838 kcal/mol, population = 1.56 %
C 14.748637 -2.862793 8.936638
C 13.639145 -3.262753 8.201160
C 12.687144 -4.102420 8.774930
C 12.829966 -4.550373 10.088732
C 13.954410 -4.148923 10.815542
C 14.906355 -3.313289 10.245591
C 11.753440 -5.377516 10.745343
C 10.963100 -4.536173 11.772749
C 9.953936 -5.437240 12.476881
O 8.697741 -5.234963 12.081571
O 10.286327 -6.266564 13.295685
C 7.667735 -6.070959 12.681930
C 6.346167 -5.659996 12.078988
C 11.902567 -0.810040 10.813981
C 13.035951 0.166886 11.133755
C 10.951376 -0.216148 9.772490
C 11.200021 -1.222950 12.115164
C 10.058185 -2.241613 11.980464
N 10.341924 -3.393927 11.118622
C 8.717374 -1.623925 11.554324
O 8.309400 -0.552972 11.940968
O 7.995170 -2.414287 10.757196
H 15.486535 -2.205000 8.495564
H 13.508846 -2.919484 7.182689
H 11.822013 -4.407223 8.197868
H 14.080482 -4.488147 11.837103
H 15.769761 -3.008370 10.823340
H 11.062331 -5.762567 9.993294
H 12.185415 -6.228983 11.271125
H 11.666494 -4.222813 12.552378
H 7.909226 -7.113159 12.473162
H 7.692146 -5.920234 13.761233
H 5.548435 -6.266903 12.510288
H 6.131478 -4.611190 12.286132
H 6.346107 -5.810939 10.998733

H 12.370345 -1.697341 10.378476
H 12.642796 1.093913 11.560323
H 13.736195 -0.265467 11.851691
H 13.594176 0.420820 10.230473
H 11.505199 0.081521 8.879906
H 10.441430 0.666112 10.166270
H 10.185313 -0.925609 9.451759
H 11.948887 -1.650685 12.786184
H 10.799825 -0.338275 12.615338
H 9.843490 -2.627033 12.982275
H 8.568004 -3.214804 10.618163
H 10.915849 -3.105756 10.335661

1a-C73, ΔG = 2.1542 kcal/mol, population = 0.99 %
C 14.696998 -6.655592 9.953677
C 13.449126 -7.012998 9.452829
C 12.496097 -6.033558 9.190473
C 12.774470 -4.687049 9.427364
C 14.030897 -4.338302 9.926049
C 14.986523 -5.313708 10.187503
C 11.720341 -3.634321 9.206237
C 10.995302 -3.230759 10.506995
C 10.368977 -4.475614 11.129445
O 11.109934 -4.933365 12.139375
O 9.352017 -4.994452 10.727542
C 10.702869 -6.184846 12.765428
C 9.667958 -5.943862 13.843966
C 11.198848 0.213246 11.638163
C 11.645018 -0.251795 13.026348
C 11.481190 1.706039 11.452146
C 9.711472 -0.067328 11.384846
C 9.345454 -1.553567 11.328727
N 9.951812 -2.262334 10.194318
C 7.818523 -1.734574 11.281687
O 7.057552 -1.202911 12.059141
O 7.396183 -2.543580 10.312435
H 15.440528 -7.416037 10.156473
H 13.217391 -8.053689 9.263360
H 11.524548 -6.317375 8.803501
H 14.261307 -3.295346 10.111129
H 15.957628 -5.027183 10.571598
H 12.172669 -2.730325 8.791704
H 10.970464 -3.987006 8.496607
H 11.732254 -2.838095 11.213033

H 10.333831 -6.853809 11.990017
H 11.627208 -6.584263 13.175875
H 9.438593 -6.888545 14.340672
H 10.045249 -5.246538 14.593230
H 8.745264 -5.545556 13.422410
H 11.785976 -0.329883 10.889452
H 11.514623 -1.325750 13.169879
H 12.701746 -0.028415 13.185226
H 11.071933 0.260267 13.804415
H 12.540194 1.925079 11.602488
H 10.910571 2.298948 12.172483
H 11.205173 2.039211 10.449268
H 9.112870 0.405308 12.165995
H 9.410321 0.391559 10.437252
H 9.659577 -2.034785 12.258535
H 8.230950 -2.846074 9.862577
H 10.279157 -1.608451 9.493430

1a-C9, ΔG = 2.1793 kcal/mol, population = 0.95 %
C 14.884806 -3.012772 9.366041
C 13.876883 -3.286263 8.448953
C 12.776579 -4.051325 8.829076
C 12.668783 -4.551119 10.127450
C 13.693102 -4.277580 11.038216
C 14.791858 -3.515598 10.662044
C 11.434242 -5.298558 10.566684
C 10.576180 -4.435783 11.519059
C 9.332929 -5.211421 11.943969
O 9.659884 -6.193778 12.788915
O 8.209911 -4.981425 11.557519
C 8.589921 -7.052199 13.281241
C 7.913410 -6.440152 14.489793
C 11.974775 -0.750178 10.878310
C 13.133372 0.117464 11.374810
C 11.204855 -0.041260 9.762156
C 11.091619 -1.154898 12.067495
C 9.895270 -2.065321 11.749234
N 10.187286 -3.197507 10.865092
C 8.670210 -1.313636 11.204005
O 8.307666 -0.229204 11.599473
O 7.989825 -1.998957 10.283635
H 15.737390 -2.412314 9.075704
H 13.940904 -2.901518 7.439006
H 11.990983 -4.256724 8.111430

H 13.625844 -4.658848 12.050414
H 15.575086 -3.309321 11.380545
H 10.833009 -5.571482 9.697483
H 11.701210 -6.216890 11.088981
H 11.165398 -4.251134 12.424406
H 7.885764 -7.229066 12.470170
H 9.093609 -7.983023 13.531178
H 7.164700 -7.135393 14.874436
H 8.637775 -6.245091 15.281751
H 7.412469 -5.507609 14.230164
H 12.423080 -1.655395 10.459541
H 13.707337 -0.396896 12.148669
H 13.814363 0.358710 10.556124
H 12.764031 1.057038 11.795314
H 11.883944 0.242774 8.955878
H 10.721346 0.864796 10.134565
H 10.430682 -0.673222 9.321477
H 11.718957 -1.673125 12.796685
H 10.706536 -0.260629 12.562366
H 9.532450 -2.472541 12.698175
H 8.506998 -2.841511 10.171117
H 10.895126 -2.936266 10.190354

1a-C84, ΔG = 2.1875 kcal/mol, population = 0.94 %
C 13.844093 -4.961640 8.427689
C 13.970316 -3.672030 8.931694
C 13.423505 -3.350934 10.171334
C 12.738901 -4.307601 10.923948
C 12.630277 -5.604065 10.411143
C 13.176490 -5.928817 9.175204
C 12.096175 -3.941006 12.237842
C 10.580061 -3.646579 12.110492
C 9.788169 -4.912360 11.754328
O 10.033794 -5.904477 12.608523
O 9.016618 -4.996888 10.823309
C 9.334565 -7.164321 12.390353
C 9.810523 -8.134027 13.444392
C 11.734283 0.112615 11.745020
C 11.648302 0.353320 13.253271
C 12.439057 1.277619 11.046327
C 10.354473 -0.111193 11.113649
C 9.625262 -1.371414 11.595865
N 10.279803 -2.594418 11.164769
C 8.164961 -1.337682 11.106129

O 7.406019 -0.424474 11.349476
O 7.781464 -2.393841 10.391480
H 14.269844 -5.215017 7.465121
H 14.497861 -2.915127 8.364929
H 13.544901 -2.347999 10.562917
H 12.125410 -6.367610 10.989532
H 13.085214 -6.939219 8.796563
H 12.227810 -4.743548 12.962185
H 12.565539 -3.046500 12.647519
H 10.225821 -3.352098 13.102254
H 8.263263 -6.975217 12.457217
H 9.561491 -7.509909 11.381843
H 9.586982 -7.764183 14.445777
H 9.302422 -9.090369 13.310482
H 10.885139 -8.302216 13.362982
H 12.338104 -0.784642 11.581205
H 11.022937 1.224258 13.469094
H 11.227722 -0.501176 13.786270
H 12.640183 0.540277 13.669503
H 13.438412 1.433169 11.457795
H 11.874880 2.205476 11.176691
H 12.539379 1.093384 9.974368
H 9.719073 0.751729 11.321069
H 10.456561 -0.172253 10.025049
H 9.558582 -1.348504 12.686785
H 8.544756 -3.027181 10.366410
H 11.013148 -2.446373 10.487237

1a-C55, ΔG = 2.2597 kcal/mol, population = 0.83 %
C 14.711029 -3.599095 7.931710
C 15.009858 -3.866537 9.266174
C 14.088735 -4.528312 10.068624
C 12.854753 -4.934482 9.552826
C 12.566582 -4.664141 8.214596
C 13.488059 -4.001426 7.407128
C 11.837556 -5.601468 10.443700
C 11.205522 -4.599569 11.432621
C 10.296455 -5.357392 12.393527
O 9.007187 -5.057674 12.250514
O 10.727573 -6.153538 13.198931
C 8.063666 -5.723635 13.137686
C 6.692911 -5.166777 12.838631
C 11.025484 0.076403 12.047307
C 12.024245 1.122812 11.545720

C 11.266652 -0.237898 13.525223
C 11.124290 -1.176422 11.167609
C 10.174975 -2.343210 11.487118
N 10.519012 -3.541024 10.703440
C 8.701194 -1.995725 11.234438
O 8.120232 -1.079321 11.773017
O 8.098431 -2.797387 10.357053
H 15.428706 -3.082880 7.306768
H 15.962655 -3.560793 9.680032
H 14.329364 -4.733648 11.105330
H 11.614890 -4.976315 7.800770
H 13.248077 -3.800319 6.370609
H 11.045088 -6.048284 9.840583
H 12.302450 -6.395801 11.027448
H 12.010441 -4.196123 12.060472
H 8.122445 -6.796318 12.953003
H 8.369530 -5.532670 14.166186
H 6.410606 -5.357009 11.802745
H 5.959959 -5.646681 13.489202
H 6.661038 -4.091629 13.017412
H 10.018907 0.486175 11.945706
H 11.847179 1.368974 10.496114
H 11.946822 2.044579 12.125976
H 13.050998 0.755707 11.635616
H 11.259785 0.679057 14.118052
H 12.238655 -0.720205 13.666604
H 10.499562 -0.896986 13.935001
H 10.981540 -0.894625 10.119493
H 12.141191 -1.575959 11.247554
H 10.245096 -2.582671 12.548669
H 8.783521 -3.494407 10.164138
H 11.088853 -3.274480 9.907347

1a-C8, ΔG = 2.2672 kcal/mol, population = 0.82 %
C 14.847075 -3.118581 9.408735
C 14.762511 -3.484769 10.750408
C 13.666568 -4.203618 11.209946
C 12.635895 -4.568465 10.339259
C 12.734754 -4.205000 8.995689
C 13.833033 -3.484438 8.531585
C 11.404781 -5.265205 10.862497
C 10.562906 -4.312471 11.740769
C 9.323905 -5.044915 12.246895
O 9.656238 -5.927344 13.194134

O 8.199600 -4.863716 11.838829
C 8.602601 -6.782047 13.726141
C 8.372033 -7.983241 12.833366
C 11.955578 -0.698448 10.673905
C 13.156518 0.176421 11.040733
C 11.135945 -0.052876 9.554715
C 11.135402 -0.996204 11.937386
C 9.921614 -1.921457 11.762673
N 10.168928 -3.137142 10.982169
C 8.674479 -1.216529 11.205263
O 8.332289 -0.097968 11.514144
O 7.950443 -1.980704 10.385738
H 15.697869 -2.552157 9.052349
H 15.549991 -3.205497 11.438958
H 13.605662 -4.478013 12.256582
H 11.943665 -4.481276 8.308442
H 13.890331 -3.205842 7.486955
H 10.791368 -5.616711 10.030838
H 11.675063 -6.131281 11.466181
H 11.163845 -4.048087 12.617840
H 8.972182 -7.074601 14.706052
H 7.699425 -6.185888 13.843981
H 9.294233 -8.550919 12.702440
H 7.629210 -8.637570 13.293605
H 8.000627 -7.680377 11.854373
H 12.363026 -1.640581 10.296593
H 13.788909 0.350629 10.167864
H 12.829245 1.148468 11.420377
H 13.769176 -0.298771 11.809753
H 11.773967 0.161368 8.695061
H 10.689796 0.886371 9.889640
H 10.327853 -0.697244 9.201825
H 11.798914 -1.456700 12.673404
H 10.781002 -0.060424 12.375514
H 9.603040 -2.231901 12.762737
H 8.458585 -2.834131 10.329287
H 10.854875 -2.953849 10.260919

1a-C7, ΔG = 2.3048 kcal/mol, population = 0.77 %
C 14.817164 -2.845786 9.045836
C 14.914502 -3.384493 10.326979
C 13.922419 -4.231239 10.805116
C 12.817671 -4.555258 10.012900
C 12.735512 -4.019490 8.726743

C 13.727703 -3.168529 8.245291
C 11.694435 -5.389693 10.575165
C 10.874068 -4.577949 11.602792
C 9.804891 -5.477817 12.213296
O 8.580532 -5.231312 11.744865
O 10.072136 -6.338369 13.023268
C 7.475020 -6.013727 12.283041
C 6.940229 -5.382670 13.551251
C 11.915619 -0.814760 10.940558
C 13.027542 0.131488 11.398511
C 11.049409 -0.154687 9.865773
C 11.113068 -1.291009 12.160303
C 9.977283 -2.287856 11.879691
N 10.319476 -3.388891 10.973783
C 8.677640 -1.631530 11.387844
O 8.242471 -0.585557 11.811480
O 8.023035 -2.359878 10.481015
H 15.586477 -2.180061 8.676045
H 15.762361 -3.140397 10.954499
H 14.001215 -4.640074 11.805722
H 11.885707 -4.264149 8.100248
H 13.644637 -2.756494 7.247576
H 11.032259 -5.721016 9.773254
H 12.082166 -6.275497 11.078244
H 11.543687 -4.322507 12.431805
H 6.735000 -6.004818 11.486359
H 7.817611 -7.033221 12.448871
H 6.631501 -4.352483 13.369266
H 6.070989 -5.945563 13.896507
H 7.690235 -5.393113 14.342299
H 12.409834 -1.682429 10.493815
H 13.669819 -0.346010 12.141722
H 13.653597 0.429989 10.555251
H 12.608338 1.037214 11.845474
H 11.670759 0.185843 9.035235
H 10.517157 0.709549 10.269685
H 10.305204 -0.837205 9.450063
H 11.805043 -1.763628 12.861532
H 10.681519 -0.430632 12.676896
H 9.683992 -2.728450 12.837683
H 8.606989 -3.152521 10.337446
H 10.958709 -3.062297 10.259104

1a-C77, ΔG = 2.3092 kcal/mol, population = 0.76 %

C 14.234536 -4.842002 8.757386
C 13.384690 -5.795761 9.312645
C 12.651992 -5.499887 10.456011
C 12.750782 -4.244230 11.064181
C 13.616772 -3.302490 10.505825
C 14.352824 -3.595127 9.360663
C 11.921163 -3.893750 12.274293
C 10.433480 -3.628709 11.938472
C 9.674411 -4.909625 11.565218
O 9.910982 -5.893964 12.431539
O 8.918513 -5.006255 10.623194
C 9.211871 -7.154476 12.218431
C 9.650950 -8.103865 13.306456
C 11.716117 0.019141 10.283897
C 12.295854 0.370097 11.654248
C 12.026219 1.114963 9.261057
C 10.200564 -0.217792 10.332905
C 9.732385 -1.318776 11.297603
N 10.237388 -2.624814 10.908589
C 8.196606 -1.326792 11.277221
O 7.522111 -0.456033 11.780492
O 7.646804 -2.353756 10.627610
H 14.804545 -5.073411 7.866545
H 13.296004 -6.773875 8.856547
H 12.008393 -6.256301 10.886403
H 13.726965 -2.334991 10.979424
H 15.018438 -2.849444 8.944284
H 11.970102 -4.688372 13.017809
H 12.315668 -2.989047 12.737240
H 9.955854 -3.283005 12.860249
H 8.140099 -6.959110 12.248801
H 9.467558 -7.522095 11.224592
H 9.395473 -7.713755 14.292404
H 9.145238 -9.061779 13.174942
H 10.727250 -8.275309 13.263704
H 12.203299 -0.895410 9.932865
H 13.368807 0.558115 11.580912
H 11.823074 1.271433 12.054242
H 12.152811 -0.431439 12.380736
H 11.634118 0.857734 8.274684
H 13.103489 1.265916 9.167766
H 11.578438 2.065419 9.564258
H 9.704628 0.709112 10.629030
H 9.842961 -0.462838 9.328117

H 10.022147 -1.048838 12.317856
H 8.381980 -2.961876 10.360494
H 11.016387 -2.579326 10.267874

1a-C26, ΔG = 2.4360 kcal/mol, population = 0.62 %
C 14.738195 -3.704269 7.940708
C 15.001861 -4.088003 9.253967
C 14.019979 -4.720498 10.006554
C 12.759879 -4.981115 9.460746
C 12.507381 -4.596254 8.143372
C 13.489414 -3.961816 7.386479
C 11.680739 -5.615416 10.301216
C 11.115814 -4.623197 11.340413
C 10.120100 -5.355545 12.231664
O 8.850828 -5.081777 11.930538
O 10.473562 -6.118744 13.103380
C 7.806508 -5.674594 12.755754
C 7.545449 -4.817799 13.976623
C 11.015109 0.074024 12.209116
C 12.024666 1.141819 11.779062
C 11.211386 -0.297390 13.680225
C 11.146031 -1.143089 11.284972
C 10.197951 -2.328194 11.535076
N 10.538150 -3.467296 10.666313
C 8.725409 -1.955994 11.310304
O 8.131776 -1.125026 11.961558
O 8.143221 -2.626425 10.316598
H 15.503007 -3.210652 7.354623
H 15.974139 -3.895527 9.690085
H 14.232658 -5.017380 11.027065
H 11.535624 -4.795030 7.706985
H 13.276839 -3.670085 6.365630
H 10.863780 -5.961382 9.665804
H 12.074844 -6.476784 10.841266
H 11.935045 -4.329561 12.008066
H 6.941969 -5.711693 12.097446
H 8.104535 -6.686623 13.022469
H 7.285435 -3.799682 13.684290
H 6.711113 -5.236959 14.541964
H 8.418732 -4.786112 14.628440
H 10.010731 0.485802 12.093096
H 11.875788 1.431533 10.736300
H 11.928993 2.038173 12.395165
H 13.049356 0.772435 11.881816

H 12.178566 -0.786161 13.832003
H 10.431476 -0.970662 14.039086
H 11.186551 0.595765 14.307943
H 11.025326 -0.822004 10.245384
H 12.163146 -1.539289 11.375150
H 10.275820 -2.633349 12.579389
H 8.828710 -3.300318 10.055150
H 11.175294 -3.167934 9.935872

1a-C40, ΔG = 2.5013 kcal/mol, population = 0.55 %
C 13.699213 -4.884737 8.102419
C 13.148678 -5.883934 8.901394
C 12.718594 -5.591365 10.190205
C 12.829037 -4.296056 10.704899
C 13.397589 -3.306550 9.899531
C 13.827271 -3.595506 8.607079
C 12.302109 -3.964000 12.077925
C 10.775140 -3.698605 12.090040
C 9.982643 -4.979185 11.794785
O 10.299591 -5.957165 12.643787
O 9.161428 -5.083172 10.909646
C 9.600914 -7.230712 12.511331
C 8.277763 -7.205240 13.246334
C 11.775116 0.046715 11.877598
C 11.704279 0.193425 13.398805
C 12.454102 1.263211 11.244333
C 10.392694 -0.162087 11.248057
C 9.691287 -1.462025 11.662218
N 10.375959 -2.648893 11.180243
C 8.229980 -1.444327 11.173852
O 7.855758 -2.509282 10.467329
O 7.460746 -0.539584 11.416205
H 14.033686 -5.112799 7.098356
H 13.057326 -6.893549 8.520685
H 12.302359 -6.378410 10.806685
H 13.519775 -2.302950 10.289645
H 14.265378 -2.813989 7.999026
H 12.513554 -4.774299 12.774172
H 12.789576 -3.066452 12.458865
H 10.505071 -3.417766 13.111657
H 9.472236 -7.445175 11.452002
H 10.288761 -7.952531 12.945250
H 7.597046 -6.476681 12.806234
H 7.812322 -8.190714 13.183179

H 8.424466 -6.963120 14.299736
H 12.389781 -0.829824 11.652271
H 11.071422 1.041464 13.675314
H 11.300837 -0.697077 13.883863
H 12.699009 0.367432 13.813809
H 12.545250 1.145957 10.162230
H 13.455364 1.409728 11.654464
H 11.876896 2.172087 11.436505
H 9.743677 0.675742 11.509492
H 10.485573 -0.158487 10.156880
H 9.621264 -1.497488 12.752712
H 8.629219 -3.129753 10.435909
H 11.059886 -2.459959 10.462437

1a-C46, ΔG = 2.5483 kcal/mol, population = 0.51 %
C 14.991107 -6.368958 9.738453
C 15.158993 -4.986750 9.713232
C 14.061552 -4.154199 9.524298
C 12.782042 -4.688100 9.357669
C 12.624851 -6.074413 9.381733
C 13.720779 -6.910928 9.571141
C 11.586123 -3.783738 9.210754
C 11.105583 -3.215665 10.559936
C 10.826772 -4.351443 11.540695
O 9.747177 -5.054393 11.192002
O 11.522430 -4.589305 12.501900
C 9.346637 -6.159876 12.052594
C 8.479767 -5.667647 13.192510
C 11.063851 0.315611 11.360108
C 11.777403 0.016096 12.680335
C 11.134756 1.809836 11.034969
C 9.598495 -0.139263 11.375513
C 9.398655 -1.653140 11.496616
N 9.904792 -2.410818 10.343692
C 7.910175 -1.988041 11.699711
O 7.445890 -2.916253 10.865330
O 7.216588 -1.471256 12.546470
H 15.845304 -7.018052 9.884332
H 16.145462 -4.557763 9.837820
H 14.198865 -3.079117 9.503277
H 11.636968 -6.500723 9.253688
H 13.582045 -7.984793 9.585865
H 11.831298 -2.935547 8.567079
H 10.755466 -4.320261 8.751634

H 11.917328 -2.637184 11.009779
H 8.799375 -6.824403 11.388341
H 10.243132 -6.663093 12.409685
H 7.603037 -5.143155 12.811709
H 8.142143 -6.520906 13.783722
H 9.035601 -4.996742 13.847822
H 11.586386 -0.219655 10.559374
H 11.279601 0.525361 13.510197
H 11.801870 -1.050226 12.910952
H 12.811505 0.364192 12.644444
H 12.171118 2.150311 10.989263
H 10.622679 2.396658 11.802788
H 10.663312 2.028815 10.074470
H 9.076662 0.341965 12.205154
H 9.103996 0.195821 10.457584
H 9.894605 -2.009147 12.402772
H 8.238608 -3.164871 10.315377
H 10.037966 -1.802202 9.544298

1a-C62, ΔG = 2.5521 kcal/mol, population = 0.51 %
C 12.610386 -6.104449 7.727568
C 12.776232 -4.725028 7.643280
C 13.011034 -3.978589 8.793286
C 13.080420 -4.594689 10.044398
C 12.925883 -5.980787 10.114742
C 12.690779 -6.731216 8.967290
C 13.261831 -3.782749 11.301171
C 11.928092 -3.280758 11.908540
C 11.063591 -4.488188 12.249722
O 10.083404 -4.692266 11.372428
O 11.292045 -5.194197 13.207147
C 9.200144 -5.831685 11.578694
C 8.061847 -5.467464 12.507984
C 11.054198 0.193377 12.770409
C 10.525856 -0.277574 14.127080
C 11.242998 1.712419 12.762173
C 10.132554 -0.219651 11.614888
C 10.019202 -1.734445 11.403315
N 11.284754 -2.351885 10.986583
C 8.945590 -2.036227 10.344783
O 7.787129 -1.699657 10.455582
O 9.400333 -2.686006 9.275976
H 12.427382 -6.686720 6.833295
H 12.724508 -4.229622 6.681730

H 13.144177 -2.906621 8.716367
H 12.987206 -6.476573 11.076264
H 12.572327 -7.805044 9.041776
H 13.769465 -4.371586 12.064865
H 13.881116 -2.904871 11.105849
H 12.162205 -2.811695 12.870464
H 8.844478 -6.069021 10.579069
H 9.789773 -6.662657 11.960843
H 7.376955 -6.313540 12.589151
H 8.429962 -5.228445 13.505804
H 7.505919 -4.612429 12.121317
H 12.037616 -0.262353 12.609720
H 10.446696 -1.364226 14.190638
H 11.189016 0.047570 14.930942
H 9.534066 0.141364 14.318508
H 10.287334 2.220341 12.919929
H 11.653180 2.055736 11.809971
H 11.923404 2.025448 13.556541
H 9.130473 0.177984 11.789187
H 10.491996 0.231272 10.683999
H 9.663399 -2.197281 12.324098
H 10.345777 -2.895413 9.509066
H 11.952522 -1.640100 10.715069

1a-C42, ΔG = 2.5948 kcal/mol, population = 0.47 %
C 14.674137 -3.787019 8.021717
C 14.960888 -4.008939 9.367130
C 14.009286 -4.589321 10.196441
C 12.757471 -4.959414 9.697118
C 12.482030 -4.737383 8.347251
C 13.433507 -4.154683 7.513371
C 11.711032 -5.532308 10.618849
C 11.116409 -4.445976 11.541777
C 10.174220 -5.114341 12.535888
O 8.886830 -4.895069 12.268589
O 10.581931 -5.786980 13.457299
C 7.894561 -5.525008 13.130227
C 7.595222 -6.935882 12.669699
C 11.124792 0.247576 11.904005
C 12.222220 1.192508 11.406889
C 11.256347 0.009813 13.409665
C 11.187014 -1.060235 11.104392
C 10.167108 -2.156642 11.449167
N 10.469847 -3.413138 10.744283

C 8.716848 -1.756687 11.137738
O 8.185295 -0.752179 11.556290
O 8.071282 -2.628951 10.363901
H 15.414830 -3.333263 7.375538
H 15.927181 -3.730417 9.768724
H 14.239068 -4.758823 11.242008
H 11.516958 -5.023242 7.945568
H 13.203363 -3.989257 6.468350
H 10.902808 -5.982563 10.039874
H 12.143554 -6.307874 11.251063
H 11.933485 -4.036509 12.148481
H 8.260162 -5.501876 14.155164
H 7.024358 -4.879210 13.040868
H 6.798015 -7.354937 13.286649
H 7.262708 -6.940700 11.630879
H 8.472651 -7.575642 12.764002
H 10.157310 0.716324 11.716323
H 13.213650 0.765375 11.584729
H 12.126494 1.382873 10.335417
H 12.173677 2.152078 11.925789
H 10.417734 -0.562571 13.809521
H 11.287578 0.960274 13.946353
H 12.177958 -0.533720 13.638907
H 11.098764 -0.834743 10.036788
H 12.177349 -1.505900 11.248304
H 10.191740 -2.346655 12.523652
H 8.729858 -3.366792 10.238706
H 11.045907 -3.219138 9.931766

1a-C38, ΔG = 2.6305 kcal/mol, population = 0.44 %
C 14.602504 -6.922437 11.075717
C 14.496832 -5.872001 11.984681
C 13.678233 -4.784922 11.702222
C 12.950804 -4.730075 10.510524
C 13.066375 -5.785713 9.606429
C 13.887350 -6.874431 9.884194
C 12.020775 -3.577581 10.232520
C 10.738613 -3.620057 11.097926
C 10.005545 -4.927769 10.840513
O 10.094794 -5.762418 11.875998
O 9.426043 -5.167719 9.804070
C 9.513271 -7.092665 11.731545
C 10.482898 -8.035457 11.051449
C 10.225722 1.312539 10.514335

C 9.671566 2.217676 9.410567
C 11.742685 1.473221 10.635384
C 9.818033 -0.137589 10.228418
C 10.233206 -1.194449 11.267068
N 9.805601 -2.524771 10.818910
C 9.581300 -0.910407 12.627313
O 9.946919 -0.042943 13.386795
O 8.538861 -1.701266 12.898102
H 15.240001 -7.769625 11.295101
H 15.054921 -5.899256 12.912342
H 13.603507 -3.969568 12.412640
H 12.504119 -5.757091 8.680708
H 13.963562 -7.686255 9.171676
H 12.527482 -2.630264 10.424612
H 11.720084 -3.582971 9.183370
H 11.008205 -3.581654 12.152653
H 9.304997 -7.397871 12.754158
H 8.577507 -7.004425 11.182629
H 10.064296 -9.043674 11.051303
H 10.665526 -7.737243 10.019956
H 11.436628 -8.055593 11.579507
H 9.774405 1.618239 11.461264
H 9.911206 3.263931 9.610418
H 10.102159 1.955348 8.439763
H 8.585659 2.128704 9.331494
H 12.146186 0.917445 11.482580
H 12.005978 2.523574 10.776002
H 12.242819 1.121444 9.728065
H 8.732821 -0.191111 10.100007
H 10.265786 -0.443564 9.277193
H 11.312052 -1.143938 11.420087
H 8.523503 -2.344393 12.140060
H 9.552162 -2.525983 9.837768

1a-C72, ΔG = 2.6531 kcal/mol, population = 0.43 %
C 14.974505 -6.110868 10.539854
C 14.948704 -4.992464 11.369229
C 13.937582 -4.047630 11.234844
C 12.939151 -4.203791 10.271158
C 12.976005 -5.326185 9.442673
C 13.985763 -6.274411 9.574686
C 11.809053 -3.212851 10.175934
C 10.702692 -3.476189 11.227515
C 10.134842 -4.868418 11.001775

O 10.573847 -5.740404 11.908523
O 9.398581 -5.138812 10.078369
C 10.170960 -7.134361 11.759574
C 8.823401 -7.383847 12.402996
C 9.911786 0.040500 9.351996
C 11.302743 0.667025 9.467756
C 9.021958 0.870122 8.423287
C 9.235932 -0.108156 10.722845
C 9.868904 -1.164730 11.638703
N 9.589721 -2.526675 11.173465
C 9.306633 -1.001574 13.060255
O 8.512178 -2.001612 13.443998
O 9.540020 -0.047774 13.766902
H 15.761815 -6.846872 10.643339
H 15.717952 -4.855336 12.119030
H 13.922745 -3.178763 11.882986
H 12.206253 -5.459614 8.691730
H 14.000800 -7.138956 8.922752
H 12.185748 -2.201393 10.328433
H 11.352300 -3.250402 9.185363
H 11.138043 -3.439435 12.225361
H 10.167096 -7.380822 10.699357
H 10.963752 -7.688714 12.255867
H 8.036128 -6.825909 11.896397
H 8.585151 -8.447341 12.339800
H 8.838571 -7.098842 13.455808
H 10.020449 -0.953362 8.904565
H 11.969340 0.089256 10.109856
H 11.775759 0.738994 8.486570
H 11.233094 1.675738 9.884019
H 8.043340 0.403605 8.292148
H 9.480891 0.978332 7.438590
H 8.865858 1.872468 8.831916
H 9.261093 0.853222 11.239930
H 8.180981 -0.367027 10.588646
H 10.942100 -0.978680 11.718427
H 8.535154 -2.630327 12.672385
H 9.156677 -2.540056 10.257982

1a-C30, ΔG = 2.6845 kcal/mol, population = 0.40 %
C 13.776489 -4.812925 8.434998
C 13.177571 -5.815479 9.194074
C 12.671374 -5.527833 10.455797
C 12.751585 -4.235876 10.983283

C 13.367591 -3.242817 10.218474
C 13.874142 -3.526257 8.952779
C 12.145572 -3.912824 12.325786
C 10.614989 -3.675722 12.258769
C 9.848107 -4.977046 11.983126
O 10.132187 -5.913293 12.889473
O 9.055498 -5.122769 11.078052
C 9.428409 -7.188540 12.800435
C 10.114473 -8.136016 11.839516
C 11.583186 0.102468 11.869498
C 11.467769 0.374168 13.370470
C 12.262273 1.273099 11.154853
C 10.220935 -0.179688 11.224273
C 9.536474 -1.463015 11.712108
N 10.239794 -2.664276 11.296370
C 8.077214 -1.491160 11.218763
O 7.288921 -0.595297 11.431057
O 7.728252 -2.583205 10.541337
H 14.170769 -5.036070 7.451747
H 13.107894 -6.823282 8.804122
H 12.218395 -6.316433 11.041809
H 13.464565 -2.240918 10.619051
H 14.347945 -2.742035 8.375750
H 12.336101 -4.718218 13.033858
H 12.595651 -3.006001 12.729905
H 10.295637 -3.362893 13.256626
H 9.448301 -7.567904 13.819251
H 8.398726 -6.993041 12.507009
H 11.150714 -8.308229 12.133788
H 9.594705 -9.095891 11.852937
H 10.091925 -7.751261 10.820186
H 12.216702 -0.778944 11.734903
H 10.813876 1.230648 13.557579
H 11.065347 -0.480293 13.917227
H 12.447471 0.600236 13.796122
H 11.666195 2.184953 11.252046
H 12.387798 1.066189 10.089748
H 13.248366 1.471266 11.579786
H 9.550020 0.660217 11.413383
H 10.338854 -0.249387 10.137722
H 9.463570 -1.436124 12.802834
H 8.512677 -3.190366 10.534910
H 10.948207 -2.498455 10.596670

1a-C11, ΔG = 2.6989 kcal/mol, population = 0.39 %
C 14.776695 -2.972654 9.056706
C 13.703081 -3.437899 8.306569
C 12.725245 -4.229923 8.904245
C 12.807183 -4.566224 10.255945
C 13.895997 -4.099855 10.998084
C 14.872829 -3.310271 10.405017
C 11.701720 -5.343056 10.926343
C 10.880818 -4.432823 11.867044
C 9.829060 -5.277353 12.579112
O 8.585735 -5.036959 12.160116
O 10.123295 -6.090053 13.428329
C 7.509107 -5.828196 12.742562
C 7.345539 -7.142067 12.008226
C 11.892652 -0.765342 10.675803
C 13.044992 0.201535 10.956379
C 10.961544 -0.203023 9.599887
C 11.172903 -1.099956 11.990583
C 10.020456 -2.112429 11.899320
N 10.310241 -3.320988 11.121051
C 8.695644 -1.510340 11.405733
O 8.285105 -0.418525 11.725649
O 7.990209 -2.336471 10.630042
H 15.534249 -2.351341 8.596461
H 13.620737 -3.182098 7.257794
H 11.887162 -4.585051 8.316186
H 13.975318 -4.351926 12.049116
H 15.707478 -2.953103 10.994889
H 11.035766 -5.772078 10.175174
H 12.106253 -6.161877 11.521092
H 11.553370 -4.084317 12.659048
H 7.721461 -5.978428 13.799450
H 6.631800 -5.194783 12.635345
H 8.232166 -7.766606 12.117667
H 6.492440 -7.682886 12.422295
H 7.160492 -6.971761 10.946832
H 12.344942 -1.682621 10.288151
H 12.669228 1.154550 11.339243
H 13.610793 0.403458 10.044658
H 13.734885 -0.211986 11.695058
H 11.527326 0.034776 8.696974
H 10.472082 0.710302 9.945566
H 10.180560 -0.909893 9.312711
H 11.910283 -1.500652 12.690408

H 10.778855 -0.185330 12.439387
H 9.782256 -2.428229 12.919772
H 8.561157 -3.147119 10.552222
H 10.919169 -3.093079 10.344550

1a-C67, ΔG = 2.7064 kcal/mol, population = 0.39 %
C 14.931720 -3.210106 9.233874
C 13.916841 -3.533705 8.341146
C 12.781838 -4.205288 8.788509
C 12.648007 -4.566300 10.129407
C 13.676356 -4.238682 11.017789
C 14.808606 -3.565992 10.575307
C 11.396375 -5.250004 10.621783
C 10.571752 -4.332038 11.549996
C 9.312118 -5.066599 11.994506
O 9.605927 -5.989217 12.913750
O 8.200554 -4.861371 11.563091
C 8.512337 -6.820515 13.396669
C 9.084585 -7.777570 14.414223
C 11.374443 0.013277 11.103117
C 11.893376 -0.609754 9.803825
C 12.460398 0.885401 11.740121
C 10.885619 -1.010120 12.140302
C 9.784088 -1.994426 11.715972
N 10.216135 -3.105435 10.855304
C 8.572350 -1.305951 11.073407
O 8.063305 -1.972041 10.037044
O 8.090577 -0.272385 11.479174
H 15.812813 -2.683650 8.889738
H 14.002585 -3.259040 7.297391
H 11.989817 -4.448248 8.090231
H 13.589438 -4.511786 12.062959
H 15.596090 -3.318968 11.276171
H 10.775427 -5.542229 9.773209
H 11.646061 -6.153738 11.178197
H 11.167381 -4.134582 12.449027
H 7.753082 -6.168424 13.828367
H 8.075955 -7.341212 12.544094
H 9.523487 -7.238353 15.254665
H 8.287278 -8.417225 14.796225
H 9.849217 -8.413550 13.966342
H 10.529120 0.660580 10.853039
H 12.320974 0.161341 9.159937
H 12.678980 -1.344119 9.999879

H 11.101939 -1.092326 9.226314
H 12.780111 1.672121 11.053770
H 13.338549 0.284271 11.993234
H 12.102586 1.359805 12.656510
H 11.733677 -1.608406 12.488944
H 10.519477 -0.460017 13.008939
H 9.378016 -2.433936 12.631246
H 8.644092 -2.777294 9.958902
H 10.993889 -2.819300 10.275277

1a-C79, ΔG = 2.7705 kcal/mol, population = 0.35 %
C 14.309260 -4.650465 9.082330
C 13.740804 -5.774603 9.675379
C 12.952781 -5.641073 10.813052
C 12.717366 -4.384317 11.376943
C 13.304129 -3.266457 10.780715
C 14.092518 -3.395062 9.641415
C 11.815965 -4.233394 12.572153
C 10.318911 -4.043272 12.191821
C 9.818375 -5.268883 11.440168
O 9.791337 -6.340495 12.236561
O 9.518551 -5.282617 10.268678
C 9.402272 -7.606959 11.631824
C 9.489801 -8.666320 12.703489
C 10.848490 0.908635 11.407879
C 11.060651 1.812432 10.190267
C 12.056547 0.972144 12.345508
C 10.557791 -0.519236 10.931128
C 10.254496 -1.563724 12.021574
N 10.016894 -2.861803 11.391517
C 8.989184 -1.164355 12.790233
O 7.881800 -1.770292 12.346707
O 8.964166 -0.358202 13.690621
H 14.923588 -4.753907 8.196895
H 13.914474 -6.756777 9.253717
H 12.521170 -6.522302 11.272643
H 13.157217 -2.287955 11.220571
H 14.540050 -2.515907 9.194940
H 11.883034 -5.109733 13.216454
H 12.123158 -3.374998 13.169738
H 9.753719 -3.982515 13.124526
H 8.390954 -7.501979 11.239148
H 10.075649 -7.809486 10.798754
H 9.203727 -9.631079 12.281468

H 10.506800 -8.748714 13.089062
H 8.816753 -8.441306 13.531706
H 9.975060 1.276001 11.952791
H 10.194807 1.792794 9.524651
H 11.226191 2.846383 10.499273
H 11.934230 1.490674 9.615911
H 12.271274 2.005981 12.623390
H 12.947303 0.565899 11.856974
H 11.889255 0.411782 13.266060
H 9.718288 -0.505123 10.230348
H 11.425577 -0.884116 10.373181
H 11.072076 -1.584410 12.743722
H 8.210418 -2.416398 11.669412
H 10.446254 -2.926886 10.476826

1a-C50, ΔG = 2.8859 kcal/mol, population = 0.29 %
C 14.679371 -6.348922 11.243381
C 14.456171 -5.350630 12.189097
C 13.548113 -4.331667 11.926889
C 12.846395 -4.294836 10.719278
C 13.079494 -5.297725 9.778493
C 13.990804 -6.317459 10.035810
C 11.821288 -3.221193 10.460519
C 10.532286 -3.409203 11.294523
C 9.940922 -4.778838 10.995443
O 10.100187 -5.624321 12.013078
O 9.403580 -5.050890 9.944301
C 9.654280 -7.001815 11.831318
C 10.710288 -7.826629 11.126938
C 10.341366 0.073629 9.249770
C 11.711350 0.628700 9.644164
C 9.718127 0.918639 8.136000
C 9.384365 0.007308 10.448211
C 9.747028 -1.050188 11.500192
N 9.495185 -2.411979 11.018072
C 8.910521 -0.809340 12.767606
O 8.009443 -1.762204 13.008734
O 9.036507 0.162008 13.477819
H 15.386763 -7.142906 11.446879
H 14.992556 -5.365018 13.129737
H 13.383193 -3.556547 12.666556
H 12.538975 -5.282520 8.839610
H 14.157732 -7.089039 9.294642
H 12.238010 -2.241465 10.696820

H 11.544622 -3.215376 9.404977
H 10.773407 -3.365620 12.355553
H 9.479349 -7.353218 12.845281
H 8.713065 -6.992122 11.284607
H 11.663471 -7.764346 11.652536
H 10.393535 -8.871119 11.104133
H 10.857547 -7.488261 10.102274
H 10.481819 -0.938738 8.856224
H 11.615919 1.655081 10.008850
H 12.190294 0.042163 10.429821
H 12.385650 0.637285 8.785789
H 8.764204 0.500104 7.808246
H 10.380806 0.971586 7.269938
H 9.537056 1.940207 8.481966
H 9.357727 0.982495 10.938701
H 8.367448 -0.198512 10.099314
H 10.788249 -0.918783 11.801461
H 8.161372 -2.420551 12.277382
H 9.241511 -2.430838 10.037818

1a-C13, ΔG = 2.9135 kcal/mol, population = 0.27 %
C 14.805161 -3.828874 8.436012
C 14.880996 -3.978616 9.819280
C 13.811644 -4.519291 10.522339
C 12.649400 -4.919642 9.857187
C 12.585914 -4.770518 8.471509
C 13.656219 -4.228909 7.763437
C 11.469014 -5.446675 10.633555
C 10.780596 -4.321460 11.435703
C 9.621249 -4.892297 12.247032
O 10.041774 -5.754454 13.162722
O 8.452393 -4.589871 12.085124
C 9.043909 -6.419953 13.996534
C 8.478178 -7.636429 13.295865
C 11.255262 0.210273 11.879611
C 12.297693 1.217579 11.389227
C 11.543029 -0.196626 13.327578
C 11.224070 -1.005842 10.942153
C 10.062822 -1.976258 11.155602
N 10.284835 -3.292474 10.548249
C 8.717775 -1.438835 10.646243
O 8.544542 -0.327403 10.199513
O 7.700559 -2.301452 10.741396
H 15.637706 -3.405634 7.888466

H 15.775068 -3.674603 10.349204
H 13.878750 -4.631716 11.598269
H 11.692448 -5.080453 7.942220
H 13.589450 -4.119509 6.688269
H 10.739804 -5.891261 9.953742
H 11.784856 -6.219073 11.333771
H 11.508479 -3.937083 12.164639
H 9.595234 -6.690583 14.893484
H 8.269574 -5.698459 14.249462
H 7.791370 -8.151436 13.970114
H 7.928299 -7.354370 12.398080
H 9.273777 -8.330319 13.021807
H 10.275346 0.693084 11.842341
H 12.332229 2.095008 12.038676
H 13.295435 0.768548 11.380446
H 12.073262 1.555621 10.375152
H 10.790588 -0.880358 13.725395
H 11.562474 0.680139 13.978173
H 12.516193 -0.690931 13.401475
H 11.203226 -0.667803 9.903030
H 12.152374 -1.571364 11.074173
H 9.915816 -2.132579 12.228101
H 8.019399 -3.172727 11.085276
H 10.880599 -3.205327 9.734902

1a-C56, ΔG = 2.9192 kcal/mol, population = 0.27 %
C 14.907928 -6.765195 10.896207
C 14.914587 -5.623433 11.693475
C 14.025906 -4.586208 11.433731
C 13.118618 -4.672010 10.375543
C 13.122009 -5.819100 9.580749
C 14.009609 -6.859591 9.837826
C 12.113127 -3.575135 10.143033
C 10.891432 -3.680533 11.090026
C 10.189972 -5.005602 10.839969
O 10.449417 -5.897206 11.795981
O 9.508287 -5.217689 9.861151
C 9.901347 -7.239521 11.636374
C 8.485678 -7.316336 12.167634
C 10.198829 1.245935 10.687462
C 9.566791 2.161324 9.635229
C 11.714060 1.451640 10.745441
C 9.823262 -0.208522 10.379760
C 10.322055 -1.279668 11.365658

N 9.909429 -2.610016 10.904403
C 9.736277 -1.050488 12.765913
O 8.735712 -1.881670 13.071406
O 10.115291 -0.190353 13.527194
H 15.600021 -7.573250 11.097006
H 15.613842 -5.540424 12.516215
H 14.036042 -3.699590 12.057455
H 12.422939 -5.898975 8.756590
H 14.000387 -7.742169 9.210379
H 12.578230 -2.600819 10.299244
H 11.746673 -3.604756 9.115207
H 11.232689 -3.660985 12.124540
H 9.955079 -7.511314 10.583721
H 10.580963 -7.868120 12.206551
H 8.129650 -8.345995 12.097966
H 8.447784 -7.011612 13.214325
H 7.813929 -6.681957 11.589597
H 9.781990 1.511942 11.661729
H 9.959319 1.937780 8.638918
H 8.481593 2.039950 9.603604
H 9.783307 3.209177 9.852263
H 12.171374 0.888833 11.560016
H 11.951381 2.506070 10.900995
H 12.183834 1.137563 9.808538
H 8.735175 -0.293846 10.302702
H 10.232792 -0.473534 9.399540
H 11.405781 -1.201237 11.463152
H 8.700409 -2.506533 12.298688
H 9.589733 -2.592561 9.943052

1a-C94, ΔG = 2.9512 kcal/mol, population = 0.26 %
C 14.624849 -7.057082 9.825754
C 15.049467 -5.817020 10.296093
C 14.231733 -4.701072 10.157602
C 12.980195 -4.805781 9.548040
C 12.566803 -6.051276 9.074048
C 13.381351 -7.170849 9.212187
C 12.068748 -3.610023 9.458289
C 11.300316 -3.340274 10.769183
C 10.481887 -4.578842 11.124473
O 11.089239 -5.306454 12.062699
O 9.436454 -4.874009 10.590399
C 10.480199 -6.577690 12.433210
C 9.420589 -6.383087 13.497275

C 10.649459 0.776940 11.265626
C 11.341800 1.963352 11.943086
C 9.354940 1.226152 10.584466
C 10.442401 -0.341720 12.297901
C 9.804068 -1.644465 11.792597
N 10.402969 -2.207679 10.576492
C 8.280091 -1.540149 11.604119
O 7.538125 -1.023640 12.408322
O 7.831708 -2.120204 10.491132
H 15.260966 -7.926618 9.933076
H 16.018688 -5.719272 10.769143
H 14.567151 -3.738556 10.526313
H 11.598135 -6.145934 8.597705
H 13.045307 -8.130157 8.838773
H 12.646603 -2.711794 9.229115
H 11.337039 -3.747001 8.660535
H 12.021231 -3.169069 11.574351
H 10.073654 -7.040194 11.535612
H 11.314776 -7.171290 12.798510
H 9.840686 -5.893169 14.376723
H 8.591410 -5.784479 13.120661
H 9.031181 -7.356644 13.801146
H 11.333392 0.413201 10.489543
H 12.285672 1.662522 12.402968
H 11.553597 2.754480 11.221211
H 10.704859 2.383953 12.726099
H 8.888697 0.427933 10.004455
H 9.556109 2.049260 9.896038
H 8.628389 1.572336 11.323158
H 11.412935 -0.597303 12.729864
H 9.821850 0.031092 13.115752
H 9.911520 -2.379977 12.595412
H 8.651135 -2.473150 10.050144
H 10.864461 -1.490136 10.031853

1a-C70, ΔG = 2.9612 kcal/mol, population = 0.25 %
C 14.846136 -6.464411 10.074804
C 13.584340 -6.989051 9.815007
C 12.514483 -6.137027 9.557003
C 12.689166 -4.753317 9.556178
C 13.960923 -4.236565 9.813850
C 15.032367 -5.084041 10.070780
C 11.518962 -3.831753 9.331797
C 11.009194 -3.185416 10.634414

C 10.666682 -4.260994 11.661924
O 9.539709 -4.906951 11.353803
O 11.353327 -4.499640 12.629471
C 9.093867 -5.965586 12.250452
C 9.742644 -7.287994 11.901372
C 11.113528 0.411673 11.028875
C 11.837016 0.235829 12.365944
C 11.238814 1.854588 10.533383
C 9.631981 0.021705 11.114981
C 9.380376 -1.461823 11.404478
N 9.844930 -2.355647 10.335486
C 7.885373 -1.717978 11.661873
O 7.223701 -1.084364 12.453037
O 7.374238 -2.716500 10.943434
H 15.680038 -7.125170 10.275421
H 13.431220 -8.061039 9.812220
H 11.533741 -6.549800 9.355374
H 14.112371 -3.163207 9.811635
H 16.013084 -4.668556 10.266312
H 11.804038 -3.018418 8.659661
H 10.692235 -4.371001 8.868453
H 11.823878 -2.609109 11.081955
H 9.313751 -5.665258 13.273241
H 8.016485 -5.994216 12.105990
H 10.822819 -7.245306 12.037833
H 9.343312 -8.066642 12.554068
H 9.527889 -7.564977 10.868469
H 11.599328 -0.233425 10.288149
H 12.884252 0.531059 12.277021
H 11.375961 0.859547 13.136789
H 11.819462 -0.796423 12.719617
H 10.766820 2.546686 11.236518
H 10.757462 1.982689 9.561424
H 12.286991 2.142987 10.432652
H 9.144022 0.611222 11.893754
H 9.134660 0.273735 10.172459
H 9.879980 -1.733252 12.337903
H 8.145083 -3.055229 10.412512
H 9.997651 -1.840069 9.476695

1a-C22, ΔG = 2.9964 kcal/mol, population = 0.24 %
C 14.920595 -3.109486 9.110337
C 13.878904 -3.397140 8.236365
C 12.771315 -4.113301 8.683069

C 12.692005 -4.555896 10.003668
C 13.747361 -4.264700 10.872848
C 14.852044 -3.546936 10.431520
C 11.469905 -5.289945 10.497797
C 10.658934 -4.438567 11.498965
C 9.421650 -5.214988 11.934581
O 9.754399 -6.174630 12.803124
O 8.299425 -5.007893 11.533312
C 8.691296 -7.036610 13.304173
C 7.986338 -6.401517 14.484343
C 11.375682 -0.058484 11.270960
C 11.878148 -0.597061 9.927811
C 12.456693 0.802740 11.930816
C 10.935124 -1.147331 12.262303
C 9.839243 -2.127871 11.815766
N 10.268244 -3.180800 10.883386
C 8.599149 -1.428613 11.242724
O 8.077498 -2.045049 10.182383
O 8.108051 -0.429654 11.718147
H 15.780260 -2.547870 8.767589
H 13.922150 -3.059774 7.208504
H 11.958606 -4.328837 7.999641
H 13.703832 -4.602198 11.901638
H 15.661165 -3.328889 11.117220
H 10.826913 -5.548849 9.654863
H 11.754320 -6.216834 10.996443
H 11.275812 -4.283363 12.392120
H 8.002726 -7.249079 12.488233
H 9.206827 -7.950852 13.588610
H 7.247365 -7.100134 14.881430
H 8.696370 -6.166686 15.278485
H 7.469739 -5.488241 14.189465
H 10.510790 0.581864 11.075931
H 11.084624 -1.063925 9.340050
H 12.275865 0.217799 9.319653
H 12.682305 -1.324443 10.065886
H 13.353769 0.209482 12.130281
H 12.110306 1.217338 12.880031
H 12.742366 1.633616 11.282624
H 11.803808 -1.747240 12.552651
H 10.585037 -0.653104 13.170276
H 9.466913 -2.624525 12.716046
H 8.671689 -2.832626 10.044865
H 11.028716 -2.849023 10.304614

1a-C21, ΔG = 3.0309 kcal/mol, population = 0.23 %
C 14.885716 -3.267951 9.147859
C 13.827359 -3.619793 8.318473
C 12.716761 -4.274938 8.844319
C 12.651309 -4.592089 10.201223
C 13.722875 -4.236710 11.025507
C 14.830681 -3.579649 10.504851
C 11.427954 -5.260057 10.778155
C 10.651520 -4.313294 11.719419
C 9.416878 -5.035845 12.244919
O 9.758765 -5.918455 13.188630
O 8.288343 -4.851211 11.850518
C 8.709989 -6.772136 13.731891
C 8.483253 -7.983005 12.851341
C 11.358962 0.036338 11.024985
C 11.835082 -0.638725 9.735297
C 12.438200 0.987267 11.551112
C 10.971431 -0.944561 12.143145
C 9.865797 -1.970525 11.847332
N 10.261644 -3.108620 11.004896
C 8.597280 -1.332509 11.263419
O 8.044619 -2.038237 10.277558
O 8.113896 -0.300278 11.671274
H 15.747924 -2.753861 8.742604
H 13.860170 -3.379510 7.263299
H 11.890971 -4.540404 8.194984
H 13.688977 -4.476277 12.081873
H 15.652630 -3.311055 11.156508
H 10.763745 -5.576957 9.972313
H 11.706897 -6.146701 11.347879
H 11.290951 -4.089868 12.581300
H 9.082786 -7.053369 14.713883
H 7.804683 -6.178515 13.845346
H 9.406944 -8.549666 12.726616
H 7.741930 -8.634587 13.317843
H 8.111533 -7.691317 11.869027
H 10.474522 0.634410 10.787147
H 12.193300 0.110822 9.026865
H 12.661509 -1.327709 9.928359
H 11.036695 -1.186600 9.230283
H 12.688933 1.743109 10.804132
H 13.353197 0.438099 11.791792
H 12.108040 1.502138 12.455985

H 11.857071 -1.506071 12.458153
H 10.653601 -0.359845 13.008093
H 9.537745 -2.375159 12.808670
H 8.637303 -2.833817 10.188681
H 11.010869 -2.840908 10.380521

1a-C43, ΔG = 3.0729 kcal/mol, population = 0.21 %
C 14.151590 -4.471247 8.833473
C 14.006618 -3.261077 9.504239
C 13.332094 -3.215420 10.720300
C 12.790047 -4.371806 11.284619
C 12.951756 -5.582614 10.606278
C 13.625466 -5.633560 9.390964
C 12.013877 -4.305301 12.572009
C 10.481057 -4.126449 12.359193
C 9.910038 -5.354959 11.665901
O 9.934911 -6.414997 12.479568
O 9.523642 -5.376393 10.520069
C 9.466895 -7.689424 11.948752
C 7.961373 -7.807893 12.058483
C 10.943499 0.809828 11.457947
C 10.958636 1.708954 10.218702
C 12.293121 0.858451 12.177793
C 10.557526 -0.614409 11.042504
C 10.405349 -1.646294 12.175503
N 10.083291 -2.949104 11.596189
C 9.257459 -1.234698 13.104585
O 8.099153 -1.842703 12.823996
O 9.356637 -0.417007 13.989393
H 14.676578 -4.510493 7.887448
H 14.420608 -2.352912 9.084273
H 13.238822 -2.271738 11.243087
H 12.553775 -6.494250 11.035790
H 13.742986 -6.581361 8.880616
H 12.160399 -5.212579 13.158212
H 12.369602 -3.474629 13.181871
H 10.023150 -4.071084 13.349103
H 9.805349 -7.780989 10.918143
H 9.974328 -8.430102 12.562142
H 7.652740 -8.799081 11.720680
H 7.637565 -7.682529 13.092531
H 7.462154 -7.063964 11.437983
H 10.179794 1.190103 12.141272
H 11.194001 2.739881 10.490212

H 11.713072 1.370748 9.502408
H 9.990213 1.704722 9.713541
H 12.279360 0.299052 13.113946
H 12.562656 1.889362 12.415731
H 13.085083 0.445261 11.545852
H 9.621149 -0.588468 10.477999
H 11.323473 -1.000091 10.362568
H 11.313027 -1.661368 12.780432
H 8.330972 -2.496090 12.114600
H 10.390844 -3.025227 10.634575

1a-C33, ΔG = 3.1570 kcal/mol, population = 0.18 %
C 14.215405 -4.831289 8.512016
C 14.320925 -3.567858 9.082398
C 13.655431 -3.282670 10.271751
C 12.872126 -4.248226 10.906554
C 12.787026 -5.519965 10.330712
C 13.450302 -5.808793 9.144074
C 12.112596 -3.908241 12.164596
C 10.598877 -3.688208 11.923498
C 9.863786 -4.994615 11.594363
O 10.152823 -5.953656 12.474929
O 9.091669 -5.128548 10.670443
C 9.468015 -7.234374 12.337525
C 8.112422 -7.200675 13.010703
C 11.660878 0.029022 10.356475
C 12.234696 0.331029 11.740900
C 11.924129 1.187657 9.391041
C 10.156801 -0.270179 10.396417
C 9.737781 -1.419684 11.326821
N 10.309506 -2.689464 10.911041
C 8.203782 -1.500282 11.308239
O 7.491013 -0.658478 11.808505
O 7.699590 -2.556891 10.669974
H 14.731362 -5.057153 7.587446
H 14.922428 -2.803752 8.606174
H 13.756099 -2.301833 10.719708
H 12.208841 -6.293535 10.819800
H 13.374077 -6.799639 8.713940
H 12.229970 -4.695152 12.908653
H 12.510763 -2.988943 12.594609
H 10.169805 -3.358689 12.874349
H 9.388870 -7.471845 11.278314
H 10.139015 -7.942377 12.817934

H 7.655597 -8.190264 12.949359
H 8.209746 -6.933355 14.063689
H 7.448434 -6.486843 12.523422
H 12.179782 -0.846815 9.955861
H 12.143640 -0.517520 12.420858
H 13.295033 0.581206 11.670137
H 11.718331 1.180808 12.196101
H 12.994704 1.381042 9.298919
H 11.446334 2.104363 9.747730
H 11.532843 0.969784 8.394855
H 9.621944 0.624410 10.722199
H 9.809648 -0.497504 9.383782
H 10.012737 -1.164892 12.355097
H 8.459754 -3.132232 10.400616
H 11.058634 -2.594048 10.241225

1a-C53, ΔG = 3.1953 kcal/mol, population = 0.17 %
C 12.400372 -6.800212 8.827649
C 13.164159 -6.549811 9.964146
C 13.360040 -5.243841 10.398123
C 12.793412 -4.167801 9.710970
C 12.043180 -4.429322 8.562720
C 11.844980 -5.735356 8.124829
C 12.945042 -2.763467 10.235942
C 11.773959 -2.312806 11.153174
C 11.765752 -3.225034 12.377605
O 10.933477 -4.253330 12.225277
O 12.492994 -3.069181 13.334259
C 10.950554 -5.300305 13.234391
C 10.061842 -6.413151 12.734611
C 8.294296 -2.451250 12.854415
C 6.841977 -2.273552 12.405237
C 8.376511 -2.589197 14.375621
C 9.178086 -1.286850 12.379860
C 9.489782 -1.312189 10.881097
N 10.523246 -2.244040 10.403649
C 9.811868 0.084806 10.317747
O 10.606188 0.052051 9.241445
O 9.362345 1.120703 10.747018
H 12.246315 -7.816630 8.487975
H 13.608185 -7.371534 10.512071
H 13.956176 -5.057209 11.283828
H 11.610988 -3.605074 8.009144
H 11.259132 -5.920331 7.233038

H 13.868731 -2.674948 10.808966
H 12.998803 -2.049874 9.413156
H 12.017454 -1.313934 11.514565
H 11.981475 -5.626530 13.367976
H 10.595577 -4.876780 14.173674
H 10.053342 -7.225772 13.462642
H 9.037494 -6.064455 12.597741
H 10.432026 -6.802559 11.785508
H 8.675954 -3.374507 12.412920
H 6.409339 -1.372617 12.849273
H 6.752962 -2.186696 11.320321
H 6.234961 -3.126722 12.714968
H 7.770647 -3.426832 14.727478
H 8.011965 -1.682339 14.866791
H 9.404754 -2.754250 14.705034
H 8.677299 -0.343637 12.605920
H 10.113867 -1.269461 12.943414
H 8.577924 -1.591842 10.344489
H 10.147763 -3.167025 10.233338
H 10.833295 -0.906539 9.134179

1a-C86, ΔG = 3.3051 kcal/mol, population = 0.14 %
C 14.770240 -2.911444 9.468500
C 14.620562 -3.178513 10.828006
C 13.578531 -3.983654 11.270889
C 12.668235 -4.535246 10.365771
C 12.836682 -4.274080 9.006402
C 13.878631 -3.466014 8.558081
C 11.484423 -5.327827 10.858640
C 10.477739 -4.417550 11.601149
C 9.280053 -5.237887 12.086659
O 9.659288 -6.176545 12.954119
O 8.134699 -5.060753 11.733624
C 8.623298 -7.046864 13.493794
C 9.294022 -8.027416 14.425035
C 10.581102 -0.478422 9.143507
C 10.161714 -1.547657 8.130400
C 11.652226 0.433028 8.536752
C 11.103982 -1.038338 10.475728
C 10.168233 -1.950974 11.289896
N 10.031821 -3.311907 10.772671
C 8.780866 -1.315174 11.475677
O 8.615359 -0.179052 11.862557
O 7.756560 -2.120982 11.197867

H 15.577542 -2.277731 9.124205
H 15.315286 -2.755875 11.542968
H 13.465983 -4.179286 12.330911
H 12.144900 -4.702165 8.290297
H 13.987371 -3.266919 7.499435
H 10.978111 -5.804801 10.016512
H 11.797532 -6.118712 11.539450
H 10.970796 -4.037661 12.500324
H 7.890936 -6.425798 14.009616
H 8.128210 -7.545455 12.660534
H 9.794568 -7.509322 15.244003
H 8.541543 -8.693721 14.850034
H 10.027182 -8.634125 13.892031
H 9.703633 0.137736 9.363020
H 10.989838 -2.227367 7.911971
H 9.310526 -2.136679 8.475743
H 9.865312 -1.079302 7.189752
H 11.293231 0.898272 7.616474
H 12.552419 -0.139312 8.294983
H 11.936301 1.228269 9.229474
H 12.029708 -1.593920 10.306222
H 11.360952 -0.191190 11.113528
H 10.581737 -2.006351 12.300741
H 8.153593 -2.997054 10.949004
H 10.391190 -3.388321 9.834483

1a-C14, ΔG = 3.3095 kcal/mol, population = 0.14 %
C 14.855920 -3.712338 8.390885
C 13.715740 -4.005318 7.651522
C 12.613802 -4.590822 8.270615
C 12.636583 -4.890812 9.633024
C 13.790105 -4.596584 10.365365
C 14.890820 -4.012394 9.751199
C 11.425113 -5.470662 10.318225
C 10.735129 -4.421622 11.216533
C 9.550290 -5.047787 11.945201
O 9.936399 -6.009396 12.772270
O 8.389961 -4.707354 11.796052
C 8.915614 -6.687452 13.568240
C 8.601246 -5.905634 14.825585
C 11.182349 0.041152 12.160396
C 12.249774 1.086849 11.830849
C 11.388989 -0.508485 13.574515
C 11.203351 -1.076320 11.106997

C 10.032184 -2.055649 11.162301
N 10.276708 -3.300473 10.425684
C 8.712621 -1.459506 10.651823
O 8.568918 -0.315226 10.284742
O 7.682233 -2.311774 10.645066
H 15.713364 -3.256192 7.912556
H 13.680548 -3.778768 6.593301
H 11.727728 -4.817923 7.689367
H 13.825652 -4.827469 11.423733
H 15.777725 -3.792267 10.332208
H 10.707048 -5.824019 9.576060
H 11.706734 -6.319483 10.940308
H 11.451375 -4.125985 11.997141
H 8.033979 -6.832233 12.947161
H 9.362645 -7.652252 13.793878
H 7.890466 -6.471808 15.430453
H 9.502930 -5.745264 15.417891
H 8.154957 -4.939888 14.589059
H 10.205634 0.529923 12.116496
H 13.246613 0.635752 11.833700
H 12.082446 1.524686 10.844339
H 12.247619 1.895745 12.564755
H 10.618634 -1.228159 13.858871
H 11.366450 0.298652 14.309608
H 12.358974 -1.008055 13.654394
H 11.239204 -0.638378 10.106265
H 12.122466 -1.657927 11.233444
H 9.837148 -2.325860 12.203668
H 7.978218 -3.212088 10.930139
H 10.907263 -3.130090 9.652590

1a-C24, ΔG = 3.3139 kcal/mol, population = 0.14 %
C 14.362820 -4.619711 8.617695
C 14.428760 -3.367069 9.216950
C 13.769164 -3.135428 10.421056
C 13.033194 -4.144666 11.044310
C 12.985624 -5.404505 10.437833
C 13.642361 -5.639310 9.235431
C 12.288157 -3.862666 12.326129
C 10.754184 -3.782629 12.138403
C 10.160706 -5.185695 11.960167
O 9.269325 -5.261394 10.968173
O 10.457459 -6.114064 12.678919
C 8.572076 -6.527967 10.777722

C 7.360185 -6.616636 11.680641
C 11.535764 -0.148448 10.128854
C 12.301962 0.367057 11.347340
C 11.589786 0.865729 8.983285
C 10.071634 -0.471384 10.454543
C 9.853248 -1.539759 11.537473
N 10.330059 -2.847120 11.107604
C 8.343524 -1.619272 11.810471
O 7.728101 -0.773242 12.418459
O 7.744078 -2.691482 11.287068
H 14.873685 -4.804089 7.681139
H 14.993184 -2.569057 8.750998
H 13.835425 -2.160987 10.888245
H 12.441710 -6.210468 10.913637
H 13.595808 -6.621410 8.781580
H 12.496476 -4.631067 13.070762
H 12.621063 -2.910075 12.738350
H 10.333668 -3.448802 13.091497
H 8.292773 -6.516620 9.726959
H 9.274365 -7.339457 10.957867
H 6.820312 -7.541574 11.469389
H 7.652219 -6.623171 12.730784
H 6.685738 -5.777582 11.505950
H 12.027985 -1.059507 9.775611
H 12.337936 -0.363522 12.157098
H 13.332182 0.609067 11.078791
H 11.835015 1.274857 11.739412
H 11.119090 1.807952 9.277587
H 11.069949 0.491753 8.098570
H 12.623026 1.079974 8.703150
H 9.572165 0.441092 10.787775
H 9.563242 -0.797396 9.541985
H 10.318941 -1.210266 12.471246
H 8.464751 -3.241770 10.887883
H 10.996857 -2.795559 10.350396

1a-C36, ΔG = 3.3471 kcal/mol, population = 0.13 %
C 14.716493 -2.934891 9.334434
C 13.779171 -3.523570 8.494036
C 12.765246 -4.316030 9.026773
C 12.670702 -4.527319 10.401964
C 13.626109 -3.941166 11.236183
C 14.640684 -3.152013 10.708803
C 11.516170 -5.300028 10.987482

C 10.555269 -4.358746 11.751195
C 9.394297 -5.159314 12.344980
O 9.832409 -6.027778 13.258863
O 8.232699 -5.020833 12.030878
C 8.855951 -6.908927 13.887696
C 8.607147 -8.136992 13.037804
C 10.492814 -0.530157 9.127184
C 9.980148 -1.640221 8.204560
C 11.539834 0.319423 8.401201
C 11.091530 -1.032116 10.450949
C 10.204786 -1.911220 11.352137
N 10.054404 -3.293997 10.900385
C 8.822976 -1.278657 11.582064
O 8.665568 -0.126570 11.922062
O 7.794331 -2.106555 11.401262
H 15.502162 -2.313236 8.924407
H 13.830794 -3.363519 7.424501
H 12.037575 -4.771473 8.365249
H 13.570930 -4.096914 12.307372
H 15.371120 -2.702279 11.369621
H 10.962324 -5.804463 10.192505
H 11.866240 -6.066871 11.677750
H 11.103293 -3.938176 12.598784
H 9.309795 -7.165399 14.841969
H 7.941678 -6.344568 14.061836
H 9.537829 -8.674960 12.852637
H 7.922915 -8.806432 13.562618
H 8.156908 -7.868863 12.081990
H 9.646004 0.118552 9.371519
H 10.777929 -2.346590 7.959125
H 9.148686 -2.195119 8.642128
H 9.621277 -1.213644 7.265828
H 11.128558 0.746722 7.484428
H 12.406741 -0.289538 8.129331
H 11.890175 1.140477 9.030442
H 12.006894 -1.595802 10.253598
H 11.382728 -0.158520 11.036184
H 10.669387 -1.920891 12.342169
H 8.187122 -2.989055 11.168836
H 10.371049 -3.406116 9.950365

1a-C75, ΔG = 3.3641 kcal/mol, population = 0.13 %
C 14.265929 -4.930462 8.999452
C 14.355354 -3.714504 9.667794

C 13.608426 -3.497627 10.822399
C 12.758691 -4.486092 11.324369
C 12.686100 -5.708411 10.649286
C 13.432667 -5.929218 9.497582
C 11.896500 -4.210298 12.529663
C 10.466471 -3.756883 12.142764
C 9.626234 -4.881957 11.526448
O 9.723435 -6.010977 12.228518
O 8.924104 -4.752070 10.548172
C 8.933136 -7.147775 11.775607
C 9.223781 -8.300096 12.706195
C 10.789210 0.278398 9.825153
C 11.156792 -0.786118 8.785857
C 11.578266 1.564662 9.561622
C 11.019697 -0.157341 11.281722
C 10.149545 -1.299018 11.837840
N 10.450278 -2.603515 11.262353
C 8.662499 -0.974734 11.613711
O 8.029112 -1.819382 10.799425
O 8.103716 -0.026030 12.117816
H 14.845347 -5.102720 8.101296
H 15.006984 -2.934620 9.294226
H 13.693706 -2.552601 11.345818
H 12.047014 -6.493215 11.033560
H 13.365584 -6.882737 8.988687
H 11.824566 -5.092451 13.163790
H 12.337256 -3.408810 13.123996
H 9.945404 -3.496838 13.068649
H 7.881830 -6.860535 11.791220
H 9.214692 -7.370329 10.746413
H 8.947067 -8.053742 13.732017
H 8.644439 -9.170592 12.394267
H 10.281518 -8.565180 12.680991
H 9.725408 0.512486 9.708992
H 11.059310 -0.377717 7.777988
H 12.194197 -1.110807 8.910279
H 10.514754 -1.664896 8.843106
H 11.402770 1.927809 8.547187
H 12.652042 1.387150 9.672186
H 11.294572 2.355213 10.259373
H 12.067395 -0.439604 11.421450
H 10.842110 0.706765 11.923672
H 10.294154 -1.311643 12.922627
H 8.692392 -2.520687 10.560995

H 11.257862 -2.590257 10.656864

1a-C59, ΔG = 3.4337 kcal/mol, population = 0.11 %
C 13.264998 -5.043739 8.447553
C 13.016752 -6.102105 9.317817
C 12.738075 -5.853961 10.656548
C 12.697248 -4.547073 11.149504
C 12.965545 -3.495685 10.271365
C 13.245127 -3.739104 8.929806
C 12.307273 -4.276570 12.577332
C 10.768292 -4.110904 12.768554
C 10.103480 -5.433824 12.418548
O 9.590222 -5.430613 11.189595
O 10.114370 -6.396310 13.154090
C 9.087826 -6.689354 10.660272
C 8.872898 -6.495236 9.178822
C 10.777974 0.763498 11.479254
C 10.519758 1.561118 10.197953
C 12.229079 0.927762 11.936507
C 10.405000 -0.704263 11.240804
C 10.542413 -1.657894 12.442108
N 10.158691 -3.008158 12.028140
C 9.602455 -1.214444 13.569201
O 9.855688 -0.339266 14.363480
O 8.433666 -1.865963 13.577716
H 13.481332 -5.235740 7.404275
H 13.041166 -7.121522 8.953526
H 12.547641 -6.683521 11.327364
H 12.969946 -2.477780 10.640904
H 13.449890 -2.909940 8.264137
H 12.631578 -5.091110 13.226010
H 12.791062 -3.369437 12.939990
H 10.587801 -3.951107 13.832424
H 9.822608 -7.466297 10.868495
H 8.164828 -6.935250 11.185814
H 8.493890 -7.420832 8.742599
H 8.147637 -5.703375 8.987538
H 9.812069 -6.240153 8.686216
H 10.127849 1.162411 12.262241
H 11.154834 1.201649 9.383149
H 9.479316 1.472037 9.877471
H 10.737530 2.620058 10.349657
H 12.917431 0.483866 11.211105
H 12.407688 0.459407 12.904930

H 12.481955 1.985693 12.031402
H 9.375400 -0.763248 10.876630
H 11.042144 -1.101162 10.444598
H 11.558609 -1.605036 12.836044
H 8.534044 -2.560164 12.877107
H 10.265923 -3.141829 11.030334

1a-C12, ΔG = 3.5285 kcal/mol, population = 0.10 %
C 14.261934 -4.658155 9.081889
C 14.194355 -3.380023 9.625677
C 13.449391 -3.152609 10.779062
C 12.757124 -4.190600 11.405100
C 12.845290 -5.473035 10.856268
C 13.589020 -5.706058 9.705313
C 11.900818 -3.923147 12.616094
C 10.395619 -3.763496 12.271370
C 9.770290 -5.080700 11.809775
O 9.889867 -6.017462 12.757855
O 9.220658 -5.251137 10.746838
C 9.298086 -7.324817 12.500659
C 10.241894 -8.221922 11.728356
C 11.249646 -0.384314 9.682151
C 12.414529 0.551381 9.347474
C 10.010311 -0.019111 8.861864
C 11.004243 -0.358071 11.197870
C 9.976347 -1.351422 11.764516
N 10.112964 -2.729172 11.289588
C 8.523911 -0.892212 11.521213
O 8.105238 0.201036 11.827858
O 7.752224 -1.828377 10.968837
H 14.840015 -4.839364 8.184659
H 14.721524 -2.559034 9.156130
H 13.415943 -2.158225 11.205071
H 12.338193 -6.297507 11.339322
H 13.646333 -6.707218 9.296625
H 12.001209 -4.727336 13.344716
H 12.223786 -3.001705 13.102592
H 9.875113 -3.501963 13.195084
H 9.095595 -7.718765 13.493766
H 8.358134 -7.179687 11.971313
H 10.435474 -7.828054 10.730820
H 11.189173 -8.337990 12.256819
H 9.789635 -9.209838 11.622401
H 11.563291 -1.392859 9.391061

H 13.319068 0.270304 9.891316
H 12.640100 0.522944 8.279869
H 12.169488 1.583522 9.612814
H 9.196476 -0.733031 8.993588
H 10.253099 -0.000613 7.797584
H 9.639305 0.970409 9.140195
H 11.951435 -0.541973 11.708115
H 10.684555 0.644002 11.492368
H 10.084397 -1.332040 12.851985
H 8.379531 -2.595969 10.833763
H 10.730569 -2.803580 10.491536

1a-C32, ΔG = 3.5793 kcal/mol, population = 0.09 %
C 14.807336 -2.822638 9.376064
C 14.697283 -3.149758 10.726156
C 13.680626 -3.991372 11.159976
C 12.757119 -4.520762 10.254830
C 12.885084 -4.198179 8.904113
C 13.901405 -3.352885 8.465354
C 11.599728 -5.353970 10.742989
C 10.599606 -4.490258 11.547507
C 9.430151 -5.349604 12.031828
O 9.847336 -6.286007 12.886113
O 8.279509 -5.203151 11.682801
C 8.849472 -7.185040 13.453737
C 8.185076 -6.565756 14.665169
C 10.588205 -0.441270 9.284958
C 10.168964 -1.470901 8.230934
C 11.653277 0.496916 8.710020
C 11.118067 -1.051635 10.592358
C 10.201375 -2.026512 11.354360
N 10.114346 -3.364381 10.769165
C 8.793156 -1.445200 11.556621
O 8.587996 -0.332487 11.989650
O 7.797317 -2.272157 11.239720
H 15.595034 -2.160947 9.039087
H 15.402920 -2.745278 11.440959
H 13.597855 -4.232829 12.213401
H 12.181290 -4.607610 8.188686
H 13.979382 -3.106422 7.413992
H 11.079440 -5.804198 9.894596
H 11.943263 -6.166008 11.383055
H 11.112882 -4.135687 12.445785
H 8.127074 -7.433781 12.678443

H 9.417183 -8.074273 13.717171
H 7.618323 -5.675803 14.391810
H 7.495955 -7.288235 15.106750
H 8.926940 -6.296333 15.418140
H 9.709127 0.161431 9.532690
H 11.000355 -2.135123 7.979902
H 9.323838 -2.080461 8.555042
H 9.864718 -0.965896 7.312018
H 11.944396 1.259838 9.435282
H 11.285467 1.002958 7.815105
H 12.550263 -0.064706 8.433708
H 12.056895 -1.578251 10.401302
H 11.352919 -0.230276 11.270998
H 10.606987 -2.118981 12.365953
H 8.223494 -3.122935 10.954915
H 10.493127 -3.377923 9.835551

1a-C39, ΔG = 3.6327 kcal/mol, population = 0.08 %
C 13.615771 -5.038587 8.418355
C 13.664543 -3.694109 8.769681
C 13.299270 -3.294582 10.052511
C 12.875223 -4.227758 11.001066
C 12.846659 -5.577928 10.639602
C 13.210314 -5.980747 9.360501
C 12.413003 -3.787388 12.366906
C 10.871711 -3.651728 12.469630
C 10.236276 -5.047248 12.426999
O 9.380966 -5.212808 11.414880
O 10.494012 -5.901795 13.245988
C 8.710337 -6.502054 11.289098
C 9.563088 -7.490773 10.523001
C 11.574162 0.144184 11.623603
C 11.705663 0.540940 13.095203
C 12.046115 1.276269 10.708635
C 10.139994 -0.256211 11.257209
C 9.631094 -1.523361 11.956434
N 10.311866 -2.727965 11.499990
C 8.113590 -1.656608 11.731866
O 7.311919 -0.818387 12.080720
O 7.740187 -2.777296 11.114849
H 13.899803 -5.352095 7.421818
H 13.991258 -2.954890 8.049105
H 13.360867 -2.246891 10.321056
H 12.543322 -6.318507 11.368589

H 13.180486 -7.031188 9.099446
H 12.735788 -4.499221 13.126249
H 12.844399 -2.818280 12.617814
H 10.650381 -3.287729 13.475757
H 8.467196 -6.863258 12.286517
H 7.792735 -6.265226 10.755881
H 8.986027 -8.401131 10.349869
H 9.857677 -7.081005 9.556293
H 10.460184 -7.756470 11.080921
H 12.226472 -0.716140 11.448506
H 11.450645 -0.278326 13.769488
H 12.730572 0.841846 13.320928
H 11.048406 1.383598 13.327240
H 11.421122 2.164431 10.837901
H 11.997466 0.981455 9.658063
H 13.076927 1.556487 10.934375
H 9.462422 0.563978 11.502523
H 10.067362 -0.411232 10.175634
H 9.752908 -1.409516 13.036519
H 8.571775 -3.300405 10.976880
H 10.924067 -2.567106 10.712820

1a-C34, ΔG = 3.6477 kcal/mol, population = 0.08 %
C 13.166716 -6.162724 8.325008
C 13.024485 -6.862637 9.518255
C 12.966915 -6.176393 10.726836
C 13.048300 -4.783477 10.764801
C 13.206935 -4.091985 9.561070
C 13.262191 -4.773788 8.350613
C 12.923479 -4.053495 12.078293
C 11.493825 -3.543748 12.378588
C 10.533181 -4.729727 12.374000
O 9.725728 -4.749424 11.314980
O 10.535813 -5.561952 13.254308
C 8.765732 -5.840185 11.201203
C 9.386914 -7.051811 10.540418
C 11.156439 0.529215 11.866702
C 11.213932 1.796882 12.723629
C 10.947484 0.882915 10.391987
C 10.080698 -0.412528 12.427352
C 9.888541 -1.756780 11.708222
N 11.120773 -2.503405 11.427859
C 9.074528 -1.641025 10.407589
O 9.564211 -2.360196 9.397958

O 8.057203 -0.993158 10.306809
H 13.210068 -6.694229 7.382731
H 12.955099 -7.943207 9.509758
H 12.851263 -6.727747 11.652111
H 13.291896 -3.012728 9.568623
H 13.383791 -4.221084 7.427326
H 13.215981 -4.708038 12.898538
H 13.590386 -3.188825 12.105154
H 11.490792 -3.174634 13.410900
H 8.385440 -6.070161 12.194886
H 7.966563 -5.418285 10.596362
H 10.167189 -7.485650 11.164072
H 8.614118 -7.805895 10.378910
H 9.819364 -6.787432 9.575197
H 12.132381 0.038194 11.963112
H 12.014821 2.458188 12.387117
H 10.272201 2.348632 12.657921
H 11.391047 1.557663 13.774406
H 9.970066 1.346023 10.238568
H 11.008768 0.009692 9.739976
H 11.712002 1.587414 10.058958
H 10.321805 -0.628139 13.470958
H 9.116046 0.099967 12.427386
H 9.255141 -2.373351 12.349753
H 10.368471 -2.801340 9.785864
H 11.897777 -1.863319 11.320276

1a-C49, ΔG = 3.7713 kcal/mol, population = 0.06 %
C 13.063876 -5.933938 7.953550
C 13.237653 -4.560444 8.098884
C 13.268459 -3.990536 9.367263
C 13.121727 -4.780044 10.509662
C 12.961122 -6.158243 10.351704
C 12.930281 -6.732517 9.085149
C 13.080656 -4.164424 11.885080
C 11.667032 -3.694608 12.310085
C 10.724306 -4.892218 12.263089
O 9.921718 -4.884455 11.200189
O 10.751519 -5.766759 13.100949
C 8.995916 -5.995604 11.031345
C 7.714649 -5.750878 11.799941
C 11.129204 0.412206 12.067460
C 11.159720 1.626166 13.000361
C 10.839075 0.844708 10.627973

C 10.127132 -0.617127 12.610590
C 9.974937 -1.927149 11.821585
N 11.231119 -2.594132 11.459732
C 9.112381 -1.776461 10.555932
O 9.615251 -2.384672 9.482357
O 8.051954 -1.193312 10.532459
H 13.040193 -6.378550 6.966627
H 13.351359 -3.932175 7.224223
H 13.409522 -2.921681 9.468918
H 12.854968 -6.786026 11.228348
H 12.804225 -7.803198 8.982876
H 13.425763 -4.881060 12.629820
H 13.742723 -3.297560 11.940225
H 11.720341 -3.404382 13.365474
H 8.821641 -6.028213 9.958654
H 9.492819 -6.911308 11.345740
H 7.011596 -6.561213 11.598343
H 7.900329 -5.717827 12.873488
H 7.253077 -4.812007 11.491270
H 12.132009 -0.031210 12.092915
H 10.189038 2.129503 13.008328
H 11.394939 1.332950 14.025751
H 11.910068 2.348953 12.673943
H 9.832792 1.261466 10.543112
H 10.919401 0.018715 9.919242
H 11.550074 1.610507 10.312096
H 10.420506 -0.878853 13.629975
H 9.138083 -0.157999 12.676459
H 9.392758 -2.607099 12.447276
H 10.455372 -2.803114 9.816045
H 11.980472 -1.917091 11.388035

1a-C96, ΔG = 3.7933 kcal/mol, population = 0.06 %
C 14.279584 -4.348217 9.156947
C 13.493433 -5.447334 9.494440
C 12.747427 -5.440792 10.667428
C 12.772750 -4.336218 11.524495
C 13.574302 -3.245132 11.180553
C 14.321176 -3.247570 10.005673
C 11.911497 -4.290013 12.760847
C 10.444578 -3.902839 12.447823
C 9.656620 -5.017477 11.746410
O 9.885247 -6.209639 12.296699
O 8.883101 -4.830858 10.833055

C 9.158514 -7.348082 11.751172
C 9.627558 -8.578742 12.488637
C 11.457411 -0.020251 10.714793
C 12.577968 1.022983 10.745951
C 10.323682 0.432699 9.792301
C 10.996556 -0.305073 12.152013
C 9.972932 -1.434741 12.352786
N 10.317990 -2.679628 11.682235
C 8.544124 -1.018216 11.949425
O 7.929476 -1.877460 11.135797
O 7.998626 -0.017805 12.359492
H 14.859411 -4.353759 8.242697
H 13.463613 -6.312429 8.843775
H 12.146075 -6.303349 10.925034
H 13.621023 -2.388753 11.842454
H 14.934737 -2.390713 9.757072
H 11.918618 -5.250291 13.274250
H 12.301155 -3.542561 13.452772
H 9.931947 -3.766957 13.404290
H 8.091890 -7.167825 11.885294
H 9.367164 -7.405626 10.682964
H 9.098800 -9.452791 12.105084
H 10.697523 -8.735180 12.345427
H 9.424154 -8.496430 13.557026
H 11.890314 -0.934112 10.293025
H 12.959219 1.213424 9.740901
H 12.212255 1.970264 11.152043
H 13.412159 0.690167 11.367480
H 9.566553 -0.339715 9.652993
H 10.716186 0.681433 8.804274
H 9.829074 1.321151 10.192498
H 11.872679 -0.555861 12.754472
H 10.571261 0.606016 12.578854
H 9.904547 -1.607638 13.430794
H 8.589885 -2.602115 10.960681
H 11.069159 -2.575716 11.014726

1a-C37, ΔG = 3.8542 kcal/mol, population = 0.06 %
C 14.162642 -4.373055 9.084873
C 13.542465 -5.543068 9.514733
C 12.767692 -5.539161 10.669373
C 12.595647 -4.368892 11.412355
C 13.235804 -3.206299 10.979046
C 14.011227 -3.204168 9.824084

C 11.713407 -4.347908 12.631493
C 10.208374 -4.103735 12.309434
C 9.625481 -5.305459 11.579379
O 9.498944 -6.349482 12.404101
O 9.336078 -5.318209 10.405027
C 8.982352 -7.595731 11.850213
C 10.076873 -8.410746 11.194455
C 11.037593 0.776003 11.398454
C 11.130286 1.678135 10.164821
C 12.382601 0.721437 12.127111
C 10.552665 -0.614739 10.972553
C 10.278789 -1.623361 12.104160
N 9.914349 -2.912633 11.519756
C 9.108223 -1.133611 12.964074
O 7.934208 -1.681856 12.630955
O 9.206292 -0.307421 13.841188
H 14.766134 -4.375105 8.186038
H 13.665387 -6.460632 8.952975
H 12.298939 -6.456731 11.001242
H 13.139446 -2.296520 11.557607
H 14.498673 -2.291261 9.505437
H 11.783344 -5.289611 13.176648
H 12.040912 -3.564898 13.315393
H 9.686752 -4.025594 13.265575
H 8.564691 -8.108688 12.713110
H 8.183151 -7.357327 11.150650
H 9.667830 -9.374002 10.883784
H 10.470194 -7.908765 10.310938
H 10.893716 -8.597535 11.892999
H 10.298676 1.206911 12.078643
H 10.167143 1.754146 9.655360
H 11.445982 2.685444 10.443448
H 11.859015 1.284330 9.450350
H 12.328112 0.144915 13.051734
H 12.717384 1.727273 12.388125
H 13.149147 0.271155 11.489655
H 9.642128 -0.517452 10.374695
H 11.308570 -1.064975 10.321851
H 11.148565 -1.682685 12.759482
H 8.165066 -2.356468 11.941336
H 10.268727 -3.015726 10.576910

1a-C58, ΔG = 3.8768 kcal/mol, population = 0.05 %
C 14.764545 -3.309162 8.373969

C 13.498811 -3.529798 7.841874
C 12.520471 -4.159730 8.606312
C 12.791552 -4.571049 9.912683
C 14.068483 -4.350921 10.433707
C 15.048443 -3.726484 9.671937
C 11.714800 -5.182005 10.771697
C 11.034862 -4.129117 11.679321
C 10.083299 -4.860002 12.616004
O 8.822120 -4.853422 12.187078
O 10.466535 -5.411481 13.624000
C 7.828024 -5.574928 12.971412
C 7.800440 -7.040280 12.592304
C 11.662510 -0.188781 11.696171
C 12.844839 -0.996693 11.156586
C 11.674851 -0.153574 13.226018
C 10.293184 -0.610208 11.133848
C 9.746452 -1.987572 11.552908
N 10.387054 -3.113346 10.859148
C 8.236644 -2.012421 11.252609
O 7.433122 -1.301939 11.815194
O 7.887465 -2.853071 10.281697
H 15.526600 -2.819640 7.780909
H 13.271186 -3.214270 6.831399
H 11.537933 -4.335020 8.184060
H 14.293105 -4.668201 11.445364
H 16.033415 -3.563047 10.090908
H 10.948405 -5.646227 10.148754
H 12.140129 -5.952217 11.415345
H 11.800286 -3.699523 12.335389
H 8.050383 -5.432391 14.027320
H 6.892816 -5.077013 12.727169
H 7.002487 -7.540736 13.144166
H 7.606248 -7.159574 11.525625
H 8.743963 -7.527697 12.837730
H 11.794319 0.840767 11.346313
H 12.818512 -1.082564 10.068079
H 13.786348 -0.514324 11.426783
H 12.876648 -2.003604 11.571887
H 11.611249 -1.157898 13.652025
H 10.840138 0.431547 13.618508
H 12.601978 0.293349 13.590428
H 9.559507 0.132928 11.449863
H 10.321320 -0.574101 10.039602
H 9.841799 -2.100730 12.634449

H 8.720716 -3.366829 10.104552
H 11.043890 -2.781153 10.164387

1a-C88, ΔG = 3.9866 kcal/mol, population = 0.04 %
C 14.929132 -3.264786 8.687281
C 13.736034 -3.278604 7.972994
C 12.614735 -3.914260 8.499354
C 12.669152 -4.538989 9.746563
C 13.875082 -4.525199 10.451327
C 14.996858 -3.894801 9.927439
C 11.440406 -5.163491 10.355324
C 10.730546 -4.201467 11.337248
C 9.569606 -4.934110 11.993248
O 10.020610 -5.771993 12.931863
O 8.405288 -4.797481 11.696397
C 9.039548 -6.579981 13.645265
C 8.444735 -5.812224 14.807078
C 11.645701 -0.350856 12.002983
C 12.820999 -1.182255 11.484002
C 11.486891 -0.512950 13.516476
C 10.321805 -0.581643 11.252034
C 9.641443 -1.952324 11.419781
N 10.266364 -3.019094 10.625302
C 8.170403 -1.808211 10.991681
O 7.861103 -2.461660 9.874654
O 7.367992 -1.121955 11.585437
H 15.802017 -2.770895 8.279619
H 13.676178 -2.796477 7.005314
H 11.689286 -3.926607 7.935755
H 13.932297 -5.009147 11.419464
H 15.924028 -3.893371 10.486733
H 10.729236 -5.442781 9.576054
H 11.710842 -6.064896 10.904619
H 11.436263 -3.949312 12.137505
H 8.275593 -6.901452 12.939632
H 9.606562 -7.444417 13.982360
H 7.768413 -6.465141 15.362140
H 9.226849 -5.471977 15.487094
H 7.877739 -4.948957 14.459028
H 11.888117 0.699961 11.811684
H 12.917695 -1.120480 10.397748
H 13.756972 -0.827591 11.920226
H 12.726724 -2.234513 11.751632
H 12.393982 -0.192350 14.032649

H 11.309490 -1.555304 13.792847
H 10.653127 0.081914 13.896038
H 9.610230 0.171429 11.594177
H 10.472462 -0.403463 10.181928
H 9.625061 -2.220078 12.477620
H 8.667776 -3.015076 9.695563
H 11.024633 -2.651638 10.064988

1a-C71, ΔG = 3.9966 kcal/mol, population = 0.04 %
C 14.434228 -7.153101 10.064968
C 13.278149 -7.224914 9.296091
C 12.483694 -6.095296 9.123775
C 12.833652 -4.880773 9.712173
C 14.000844 -4.816955 10.477208
C 14.794850 -5.943469 10.654817
C 11.950407 -3.670403 9.554284
C 11.186645 -3.293759 10.839327
C 10.349845 -4.486813 11.298381
O 10.930960 -5.119880 12.318654
O 9.303343 -4.815620 10.786504
C 10.297786 -6.335084 12.817581
C 10.705377 -7.541496 11.999434
C 10.711657 0.841893 11.018107
C 11.460966 2.038827 11.610584
C 9.446970 1.303755 10.290373
C 10.439271 -0.182309 12.129415
C 9.733858 -1.483143 11.719751
N 10.302791 -2.169511 10.554012
C 8.217016 -1.321062 11.518587
O 7.503292 -0.687634 12.262275
O 7.737027 -1.995342 10.473168
H 15.052622 -8.031086 10.202755
H 12.989913 -8.160798 8.834042
H 11.578834 -6.158495 8.531305
H 14.289178 -3.878878 10.937271
H 15.696565 -5.878406 11.250979
H 12.549117 -2.802008 9.267617
H 11.217390 -3.841152 8.764611
H 11.906927 -3.064572 11.630099
H 10.649659 -6.408406 13.843854
H 9.219125 -6.188280 12.815296
H 11.790510 -7.640721 11.967886
H 10.287148 -8.441003 12.455507
H 10.334581 -7.468940 10.977856

H 11.380771 0.384872 10.279096
H 12.382120 1.724021 12.105723
H 11.722976 2.757854 10.831999
H 10.840825 2.553970 12.349394
H 8.943366 0.488676 9.767484
H 9.695475 2.059966 9.543056
H 8.733648 1.742261 10.991903
H 11.392936 -0.456786 12.586939
H 9.837712 0.285378 12.912105
H 9.802795 -2.162501 12.574766
H 8.537829 -2.425656 10.068138
H 10.768441 -1.514185 9.939004

1a-C48, ΔG = 4.0035 kcal/mol, population = 0.04 %
C 13.282524 -5.806506 7.925071
C 13.434246 -4.441643 8.153650
C 13.306990 -3.929966 9.440563
C 13.022360 -4.770572 10.519118
C 12.883067 -6.139201 10.279023
C 13.010060 -6.655222 8.993304
C 12.819582 -4.221407 11.909011
C 11.383396 -3.705870 12.168757
C 10.375968 -4.797313 11.825490
O 10.468121 -5.806476 12.697295
O 9.615846 -4.768140 10.885955
C 9.608892 -6.964082 12.485627
C 8.245268 -6.753871 13.109108
C 11.171990 0.454189 12.136350
C 11.222077 1.590211 13.162287
C 11.022357 1.015126 10.720281
C 10.059615 -0.529546 12.528545
C 9.888329 -1.778624 11.649392
N 11.125157 -2.517161 11.368019
C 9.168952 -1.488180 10.321180
O 9.732075 -2.066278 9.261247
O 8.160691 -0.823456 10.236593
H 13.381946 -6.205356 6.923431
H 13.654763 -3.774754 7.329420
H 13.433769 -2.867644 9.606701
H 12.673438 -6.805940 11.106827
H 12.898934 -7.719553 8.827302
H 13.033628 -4.991304 12.648597
H 13.503836 -3.392050 12.100915
H 11.293135 -3.508394 13.243588

H 9.540596 -7.152214 11.415670
H 10.147025 -7.780535 12.961528
H 7.652664 -7.663593 12.994784
H 8.336003 -6.536464 14.174207
H 7.712977 -5.935505 12.624582
H 12.135429 -0.066580 12.194653
H 12.043184 2.275885 12.944554
H 10.291371 2.164070 13.145589
H 11.361515 1.205207 14.174747
H 11.097050 0.242835 9.953042
H 11.808350 1.745399 10.518337
H 10.058064 1.514279 10.599890
H 10.245645 -0.870652 13.549880
H 9.101783 -0.004441 12.542539
H 9.201084 -2.445406 12.173988
H 10.500021 -2.572162 9.643609
H 11.924020 -1.898603 11.428374

1a-C52, ΔG = 4.0117 kcal/mol, population = 0.04 %
C 15.037665 -6.397105 10.475106
C 15.073898 -5.352669 11.395464
C 14.140977 -4.324423 11.320812
C 13.159022 -4.322750 10.327897
C 13.135348 -5.370738 9.406597
C 14.066742 -6.402021 9.478480
C 12.107086 -3.245611 10.295677
C 10.963794 -3.494627 11.313266
C 10.314993 -4.834126 11.007017
O 10.743496 -5.794074 11.824911
O 9.533154 -4.996676 10.095504
C 10.282130 -7.154915 11.574778
C 8.957219 -7.415447 12.259340
C 9.226351 -0.041958 9.549558
C 9.531392 1.000610 8.470188
C 7.874396 0.241814 10.207518
C 10.392199 -0.088294 10.547757
C 10.299572 -1.120126 11.684075
N 9.912065 -2.478319 11.284627
C 9.377426 -0.660312 12.830116
O 8.539003 -1.605386 13.256347
O 9.429706 0.438067 13.334044
H 15.763580 -7.198349 10.532603
H 15.830586 -5.338244 12.170051
H 14.175026 -3.514188 12.040156

H 12.379458 -5.381004 8.630154
H 14.034733 -7.207505 8.755397
H 12.555581 -2.279176 10.529252
H 11.664596 -3.173075 9.300464
H 11.379196 -3.533725 12.319444
H 10.219108 -7.305288 10.498652
H 11.075858 -7.777511 11.980491
H 8.675344 -8.459954 12.113022
H 9.031605 -7.228523 13.331448
H 8.169593 -6.786892 11.844044
H 9.161248 -1.011789 9.042342
H 9.595358 2.000554 8.908048
H 10.480234 0.789912 7.972133
H 8.746762 1.015694 7.711318
H 7.092156 0.315141 9.449573
H 7.900623 1.185276 10.758041
H 7.575131 -0.544047 10.902667
H 11.311972 -0.284705 9.993179
H 10.507908 0.894180 11.011305
H 11.285480 -1.168173 12.154156
H 8.729017 -2.374294 12.648894
H 9.446856 -2.492044 10.384258

1a-C57, ΔG = 4.0449 kcal/mol, population = 0.04 %
C 14.935941 -6.570269 10.730618
C 14.911885 -5.455812 11.566160
C 13.996930 -4.434460 11.340085
C 13.091041 -4.509679 10.278962
C 13.125455 -5.628473 9.447159
C 14.041836 -6.652198 9.668749
C 12.062539 -3.427583 10.075325
C 10.926814 -3.482243 11.123627
C 10.224393 -4.828203 11.033348
O 10.519488 -5.614689 12.068008
O 9.495398 -5.133220 10.115115
C 9.963398 -6.963797 12.079973
C 10.825227 -7.919390 11.282519
C 8.615344 0.412244 9.955143
C 7.675904 -0.708907 9.500080
C 8.587823 1.566196 8.948344
C 10.070687 -0.035118 10.164164
C 10.348999 -1.074018 11.265614
N 9.911711 -2.439949 10.944327
C 9.698700 -0.639234 12.588523

O 8.780500 -1.491838 13.045108
O 9.979554 0.385677 13.166631
H 15.648298 -7.366508 10.906122
H 15.608218 -5.382133 12.392260
H 13.985513 -3.568994 11.992842
H 12.425649 -5.701208 8.623208
H 14.053284 -7.514645 9.014096
H 12.533389 -2.444835 10.138311
H 11.613304 -3.513500 9.084266
H 11.346183 -3.381421 12.123506
H 9.943130 -7.226381 13.134939
H 8.944767 -6.921167 11.698704
H 11.853761 -7.909238 11.644046
H 10.430360 -8.931464 11.390786
H 10.826616 -7.661959 10.224136
H 8.243372 0.798545 10.910241
H 6.679382 -0.308932 9.303083
H 8.034696 -1.167084 8.573266
H 7.566500 -1.492522 10.249941
H 9.217072 2.395640 9.277601
H 7.571594 1.942788 8.816683
H 8.950913 1.234820 7.971184
H 10.475445 -0.430664 9.227746
H 10.662171 0.847678 10.411938
H 11.425050 -1.061066 11.452713
H 8.794949 -2.232679 12.379303
H 9.516908 -2.503592 10.014571

1a-C27, ΔG = 4.1353 kcal/mol, population = 0.03 %
C 14.269366 -4.978836 9.040877
C 14.375479 -3.765176 9.711288
C 13.649928 -3.548169 10.879638
C 12.804494 -4.534859 11.392643
C 12.715646 -5.754235 10.715185
C 13.440699 -5.975616 9.550377
C 11.963167 -4.264394 12.613593
C 10.526057 -3.810191 12.255273
C 9.660779 -4.941069 11.682711
O 9.730681 -6.042764 12.432381
O 8.957318 -4.827327 10.703237
C 8.887838 -7.173933 12.059782
C 9.560009 -8.045173 11.019900
C 10.763288 0.184907 9.847307
C 11.149400 -0.895747 8.832252

C 11.528815 1.479400 9.555618
C 11.001778 -0.214604 11.312800
C 10.158093 -1.364393 11.894002
N 10.497548 -2.673568 11.353897
C 8.665465 -1.079171 11.657049
O 8.050205 -1.954387 10.861386
O 8.085224 -0.130627 12.136919
H 14.832357 -5.150474 8.132238
H 15.023918 -2.987014 9.328742
H 13.748418 -2.604854 11.404083
H 12.079550 -6.536518 11.107189
H 13.360171 -6.927376 9.040143
H 11.903490 -5.148799 13.246060
H 12.413789 -3.465156 13.203118
H 10.031868 -3.531314 13.190550
H 8.739624 -7.708230 12.995108
H 7.932884 -6.789618 11.706673
H 10.516603 -8.421358 11.384718
H 8.919388 -8.902170 10.803282
H 9.723220 -7.498140 10.091512
H 9.695632 0.398167 9.726035
H 12.193717 -1.196131 8.960293
H 10.526189 -1.785603 8.916004
H 11.039423 -0.514105 7.815272
H 11.231870 2.279707 10.236811
H 11.345746 1.817800 8.533913
H 12.605834 1.323910 9.668222
H 12.055700 -0.468905 11.458563
H 10.802790 0.658023 11.936521
H 10.297740 -1.345291 12.979365
H 8.719260 -2.654526 10.640685
H 11.311643 -2.654583 10.757835

1a-C3, ΔG = 4.2376 kcal/mol, population = 0.03 %
C 13.617067 -3.899116 6.720662
C 12.227014 -3.924920 6.679884
C 11.504005 -4.348112 7.792042
C 12.158623 -4.746982 8.958754
C 13.554914 -4.720181 8.986860
C 14.279789 -4.300654 7.877814
C 11.380466 -5.157930 10.177952
C 11.232233 -4.009388 11.211394
C 10.587558 -4.634116 12.444568
O 11.503904 -5.105858 13.293128

O 9.390578 -4.754665 12.583516
C 11.036497 -5.886306 14.434627
C 10.617196 -4.997365 15.585090
C 9.317481 -1.472767 13.404586
C 8.324374 -0.430824 12.882915
C 9.512265 -1.308279 14.914212
C 10.682873 -1.379755 12.707517
C 10.663101 -1.563293 11.187648
N 10.464059 -2.912154 10.642540
C 11.897766 -0.933655 10.516724
O 12.215599 -1.475898 9.338264
O 12.509228 0.010771 10.959774
H 14.181208 -3.570600 5.857012
H 11.703701 -3.616914 5.783356
H 10.421581 -4.367780 7.756519
H 14.076571 -5.029926 9.885106
H 15.361914 -4.287227 7.915353
H 10.378906 -5.487734 9.895265
H 11.874059 -5.997258 10.671195
H 12.226017 -3.650097 11.477652
H 10.220606 -6.525730 14.101363
H 11.893947 -6.502102 14.695287
H 9.742391 -4.403249 15.325611
H 10.365937 -5.620513 16.445627
H 11.428744 -4.328347 15.874350
H 8.897533 -2.464685 13.222490
H 8.732857 0.579193 12.982534
H 8.066024 -0.587276 11.833910
H 7.393879 -0.472966 13.452772
H 8.569974 -1.444984 15.448890
H 9.887254 -0.307134 15.146402
H 10.229786 -2.031514 15.304599
H 11.110630 -0.395555 12.909584
H 11.372283 -2.102449 13.152941
H 9.840999 -0.967211 10.781475
H 9.482327 -3.153304 10.592030
H 11.612209 -2.247449 9.215911

1a-C47, ΔG = 4.3317 kcal/mol, population = 0.03 %
C 14.263217 -4.865153 8.728439
C 13.469718 -5.896578 9.224959
C 12.786585 -5.738577 10.425131
C 12.882721 -4.547200 11.150701
C 13.691523 -3.525049 10.648379

C 14.375748 -3.679409 9.445734
C 12.086432 -4.340270 12.413978
C 10.624159 -3.915404 12.128887
C 9.774131 -5.048453 11.539924
O 9.938176 -6.185703 12.218193
O 9.018617 -4.915444 10.602813
C 9.136962 -7.338091 11.823129
C 7.774282 -7.304620 12.481788
C 10.730779 0.194383 9.920570
C 11.075530 -0.838559 8.842501
C 11.500374 1.495127 9.669041
C 11.007548 -0.277592 11.357484
C 10.191007 -1.465482 11.896791
N 10.525809 -2.742787 11.280973
C 8.688825 -1.187716 11.717963
O 8.059327 -2.039336 10.907915
O 8.113710 -0.266191 12.253507
H 14.794109 -4.988799 7.793050
H 13.385269 -6.826883 8.677199
H 12.178895 -6.549077 10.807186
H 13.794149 -2.602815 11.207843
H 14.996555 -2.874342 9.073093
H 12.075982 -5.246539 13.017319
H 12.540767 -3.548770 13.011468
H 10.156249 -3.692170 13.092150
H 9.062580 -7.351586 10.737414
H 9.723928 -8.191005 12.155582
H 7.195302 -6.444905 12.144700
H 7.225293 -8.211171 12.219957
H 7.870166 -7.264137 13.567589
H 9.662455 0.423423 9.840504
H 12.116113 -1.164981 8.931624
H 10.435769 -1.719460 8.891290
H 10.952710 -0.401861 7.849389
H 11.287045 1.885704 8.672128
H 12.578792 1.325345 9.737625
H 11.233608 2.262685 10.398490
H 12.067198 -0.527317 11.465856
H 10.815192 0.560183 12.029308
H 10.359755 -1.503550 12.977380
H 8.734271 -2.717562 10.638845
H 11.305425 -2.681414 10.642908

1a-C69, ΔG = 4.4064 kcal/mol, population = 0.02 %

C 14.633905 -7.071004 9.856810
C 14.994253 -5.727609 9.925617
C 14.062561 -4.739307 9.628383
C 12.758349 -5.075725 9.261502
C 12.409766 -6.424328 9.187803
C 13.338758 -7.416892 9.484056
C 11.737684 -4.002718 8.991243
C 11.159836 -3.336616 10.263567
C 10.467993 -4.387612 11.125512
O 11.322417 -5.015251 11.917964
O 9.274855 -4.632121 11.076653
C 10.823796 -6.111340 12.743187
C 10.266402 -5.590962 14.050739
C 10.198661 0.650417 12.329234
C 10.723301 2.080499 12.183088
C 10.890053 -0.057465 13.497940
C 10.386777 -0.112209 11.008795
C 9.682271 -1.466024 10.930106
N 10.181817 -2.344188 9.867769
C 8.159102 -1.351306 10.763207
O 7.523939 -2.519656 10.614157
O 7.540460 -0.311665 10.772124
H 15.358706 -7.841557 10.087453
H 16.002159 -5.450119 10.208290
H 14.349986 -3.695561 9.684047
H 11.400845 -6.700096 8.903613
H 13.051066 -8.459109 9.422720
H 12.188320 -3.202588 8.399280
H 10.903576 -4.405349 8.414847
H 11.990725 -2.926934 10.852169
H 10.078749 -6.662092 12.172713
H 11.698790 -6.738163 12.895553
H 9.970752 -6.435011 14.676676
H 11.018385 -5.012588 14.588886
H 9.389976 -4.965056 13.883581
H 9.127479 0.703421 12.539310
H 11.790874 2.078111 11.943256
H 10.201263 2.612549 11.384683
H 10.590625 2.644899 13.108671
H 10.508138 -1.067491 13.659063
H 10.742241 0.497250 14.426792
H 11.966784 -0.131981 13.319239
H 10.044207 0.505835 10.175097
H 11.456677 -0.289294 10.858962

H 9.821519 -1.991956 11.878554
H 8.173031 -3.263232 10.584667
H 10.508112 -1.804560 9.076405

1a-C17, ΔG = 4.4277 kcal/mol, population = 0.02 %
C 13.790794 -4.989243 8.791860
C 13.368733 -6.053103 9.584197
C 12.838364 -5.815534 10.846985
C 12.716426 -4.514020 11.338847
C 13.157864 -3.456112 10.541116
C 13.688846 -3.688798 9.276274
C 12.077790 -4.254185 12.676090
C 10.534445 -4.070730 12.592634
C 9.896955 -5.390874 12.172784
O 9.368267 -5.350225 10.950595
O 9.907227 -6.370786 12.885385
C 8.736463 -6.560146 10.436960
C 9.746188 -7.479706 9.784308
C 10.833075 0.806644 11.373000
C 10.856987 1.613189 10.071598
C 12.159322 0.949349 12.122724
C 10.499372 -0.654849 11.051659
C 10.383661 -1.617863 12.247804
N 10.079269 -2.963010 11.757116
C 9.239214 -1.173115 13.166849
O 9.331041 -0.297876 13.995566
O 8.092446 -1.825261 12.944517
H 14.204124 -5.173067 7.808244
H 13.453393 -7.068966 9.219174
H 12.516165 -6.648065 11.460171
H 13.101786 -2.442746 10.918623
H 14.026427 -2.855553 8.672696
H 12.272156 -5.078895 13.362178
H 12.499487 -3.356358 13.128639
H 10.179824 -3.892997 13.610122
H 8.213901 -7.049999 11.256440
H 8.011259 -6.187639 9.717322
H 9.219009 -8.319380 9.327213
H 10.301960 -6.956793 9.005673
H 10.451969 -7.874699 10.513718
H 10.039645 1.211086 12.006747
H 11.643453 1.248709 9.404424
H 9.905119 1.539719 9.540791
H 11.051456 2.668442 10.272739

H 12.978879 0.503705 11.551033
H 12.128213 0.470059 13.101743
H 12.397534 2.003257 12.279868
H 9.562757 -0.697915 10.488424
H 11.278304 -1.053476 10.394509
H 11.300057 -1.578305 12.838797
H 8.331682 -2.518505 12.276017
H 10.371373 -3.088489 10.796038

1a-C15, ΔG = 4.4277 kcal/mol, population = 0.02 %
C 14.279685 -4.852967 8.949756
C 14.095559 -3.522756 9.311239
C 13.407420 -3.206533 10.478761
C 12.885366 -4.208179 11.299353
C 13.090310 -5.541551 10.931944
C 13.780182 -5.862477 9.768735
C 12.076046 -3.867136 12.523759
C 10.548259 -3.793503 12.241857
C 10.042158 -5.124345 11.698043
O 10.169957 -6.086540 12.618758
O 9.608541 -5.295034 10.582918
C 9.761454 -7.436014 12.250859
C 8.275980 -7.635164 12.466119
C 11.134502 -0.323612 9.707801
C 12.266884 0.611106 9.273574
C 9.810769 0.097793 9.066380
C 11.078592 -0.365673 11.241814
C 10.104152 -1.366939 11.883808
N 10.144978 -2.726362 11.337120
C 8.645805 -0.865916 11.845665
O 7.784284 -1.761453 11.363512
O 8.300487 0.223162 12.243840
H 14.815685 -5.102339 8.042789
H 14.490591 -2.729217 8.689336
H 13.290373 -2.169354 10.762164
H 12.711822 -6.333759 11.566096
H 13.930108 -6.901276 9.502227
H 12.238204 -4.610010 13.304503
H 12.381618 -2.900455 12.926408
H 10.043817 -3.632418 13.196110
H 10.047252 -7.614186 11.215681
H 10.351118 -8.075852 12.903197
H 8.016254 -8.672886 12.248066
H 8.003079 -7.424503 13.501013

H 7.693071 -6.991142 11.807824
H 11.387780 -1.321802 9.333397
H 12.356703 0.633268 8.185989
H 12.077510 1.631797 9.617143
H 13.226867 0.291330 9.684944
H 9.493467 1.075137 9.438103
H 9.007317 -0.613428 9.260952
H 9.922122 0.169235 7.982731
H 12.076754 -0.585742 11.625020
H 10.818121 0.626121 11.617848
H 10.344898 -1.401075 12.949506
H 8.369698 -2.534582 11.120513
H 10.650551 -2.779372 10.461793

1a-C61, ΔG = 4.4321 kcal/mol, population = 0.02 %
C 14.785147 -3.080359 8.288009
C 15.090976 -3.595972 9.545013
C 14.134921 -4.309616 10.256974
C 12.860184 -4.521971 9.726745
C 12.567414 -4.011201 8.461037
C 13.522238 -3.292735 7.746162
C 11.809615 -5.233118 10.539858
C 11.129911 -4.284022 11.555173
C 10.233245 -5.117297 12.460924
O 8.939053 -5.012812 12.169964
O 10.681676 -5.820065 13.340098
C 8.006127 -5.784537 12.981467
C 6.611684 -5.461044 12.502480
C 11.633742 -0.313654 11.846190
C 12.815646 -1.038588 11.199202
C 11.716868 -0.383938 13.372817
C 10.254960 -0.749108 11.316861
C 9.782891 -2.175566 11.652121
N 10.433676 -3.217734 10.844625
C 8.265890 -2.234858 11.404193
O 7.917372 -2.955037 10.340468
O 7.454456 -1.640253 12.078762
H 15.528374 -2.521238 7.733877
H 16.074183 -3.439991 9.970969
H 14.376507 -4.702955 11.237638
H 11.586495 -4.177358 8.031506
H 13.278205 -2.901097 6.766621
H 11.039863 -5.650052 9.888214
H 12.259524 -6.052505 11.100274

H 11.908361 -3.890221 12.219932
H 8.248404 -6.840696 12.863939
H 8.156123 -5.509267 14.025252
H 6.487116 -5.727755 11.452635
H 5.890432 -6.030737 13.090795
H 6.394085 -4.399619 12.620804
H 11.712149 0.741809 11.564277
H 12.747529 -1.040287 10.109029
H 13.753846 -0.550530 11.470551
H 12.891056 -2.073391 11.531620
H 11.720669 -1.417059 13.729124
H 10.875550 0.130591 13.842425
H 12.638602 0.082100 13.726854
H 9.510049 -0.063219 11.723621
H 10.227375 -0.628044 10.228797
H 9.927765 -2.365246 12.716821
H 8.759725 -3.416112 10.083204
H 11.069607 -2.806590 10.172849

1a-C85, ΔG = 4.5036 kcal/mol, population = 0.02 %
C 15.172069 -6.480861 10.702250
C 14.224828 -6.628469 9.693983
C 13.287867 -5.625381 9.464014
C 13.283351 -4.463423 10.236741
C 14.240812 -4.323558 11.243885
C 15.178990 -5.322878 11.475991
C 12.226746 -3.408364 10.039422
C 11.081745 -3.521159 11.075396
C 10.410028 -4.876195 10.922122
O 10.753180 -5.718092 11.896459
O 9.677198 -5.148361 9.996479
C 10.233666 -7.079519 11.829149
C 8.850123 -7.166267 12.438097
C 8.661090 0.351903 10.065249
C 7.756662 -0.772969 9.552340
C 8.588715 1.558638 9.124478
C 10.132215 -0.056387 10.241184
C 10.457033 -1.130711 11.295009
N 10.048469 -2.492930 10.926658
C 9.816921 -0.764104 12.643338
O 10.086798 0.240748 13.260569
O 8.919532 -1.651388 13.074736
H 15.901832 -7.260099 10.882481
H 14.215436 -7.523429 9.084339

H 12.550921 -5.746780 8.678889
H 14.250888 -3.424456 11.849558
H 15.916633 -5.198051 12.258963
H 12.667918 -2.414569 10.129716
H 11.789720 -3.485873 9.042185
H 11.493781 -3.452071 12.081328
H 10.240776 -7.399891 10.788870
H 10.958400 -7.662979 12.391625
H 8.521701 -8.207380 12.439098
H 8.858086 -6.809392 13.468861
H 8.130795 -6.580769 11.865934
H 8.282007 0.672356 11.041806
H 7.671477 -1.596322 10.261648
H 6.748402 -0.394276 9.374293
H 8.129790 -1.172682 8.604487
H 8.958918 1.295377 8.129413
H 9.189896 2.390372 9.497687
H 7.559390 1.906104 9.016460
H 10.539646 -0.396817 9.284419
H 10.697519 0.833701 10.521723
H 11.535335 -1.094538 11.464979
H 8.938130 -2.363447 12.378736
H 9.655237 -2.534421 9.995042

1a-C98, ΔG = 4.5827 kcal/mol, population = 0.02 %
C 13.089462 -5.348105 7.777127
C 13.310080 -4.036841 8.189498
C 13.247384 -3.708965 9.539557
C 12.959012 -4.682068 10.499336
C 12.750991 -5.996063 10.074615
C 12.813918 -6.328429 8.725046
C 12.822383 -4.324407 11.957517
C 11.409752 -3.813944 12.334786
C 10.362251 -4.832763 11.907803
O 10.476648 -5.949361 12.633975
O 9.551626 -4.667785 11.026315
C 9.575020 -7.051678 12.325348
C 8.249597 -6.891691 13.039662
C 10.566732 0.709704 12.015479
C 11.936439 0.534370 11.351300
C 10.579343 1.929459 12.941313
C 10.087462 -0.516346 12.809965
C 9.906969 -1.842456 12.046744
N 11.163406 -2.524578 11.699373

C 9.081937 -1.622811 10.770235
O 7.959828 -1.166910 10.772894
O 9.717611 -1.946373 9.645825
H 13.138185 -5.604128 6.726267
H 13.533841 -3.268710 7.459701
H 13.427903 -2.687442 9.849583
H 12.537431 -6.765405 10.806856
H 12.649964 -7.353086 8.415348
H 13.038991 -5.191391 12.579642
H 13.535386 -3.543801 12.231419
H 11.362307 -3.745704 13.428606
H 9.449235 -7.102936 11.245304
H 10.112266 -7.932966 12.667809
H 7.628116 -7.768095 12.845901
H 8.398030 -6.809247 14.117205
H 7.716412 -6.009316 12.686485
H 9.833674 0.901743 11.225257
H 11.926555 -0.224749 10.568314
H 12.250871 1.469120 10.883158
H 12.700181 0.265346 12.087745
H 9.599225 2.094055 13.393789
H 10.854842 2.831824 12.391982
H 11.304704 1.794192 13.748754
H 10.772540 -0.708307 13.641936
H 9.121338 -0.269896 13.253283
H 9.304765 -2.496968 12.678337
H 10.556123 -2.378483 9.962376
H 11.953716 -1.923292 11.886894

1a-C31, ΔG = 4.5927 kcal/mol, population = 0.02 %
C 14.838120 -6.536268 10.987189
C 14.096949 -6.385603 9.820365
C 13.215741 -5.317012 9.686379
C 13.065975 -4.382711 10.710877
C 13.819377 -4.539762 11.877077
C 14.697516 -5.607964 12.016416
C 12.078330 -3.252150 10.580894
C 10.817044 -3.442641 11.457600
C 10.161570 -4.772070 11.116935
O 10.372012 -5.687630 12.061233
O 9.536427 -4.957983 10.095630
C 9.870995 -7.038189 11.827555
C 10.850886 -7.847786 11.005600
C 9.514480 -0.020339 9.357763

C 10.039873 0.953960 8.299643
C 8.087703 0.348675 9.769896
C 10.497486 -0.062330 10.536233
C 10.174217 -1.040233 11.678125
N 9.803190 -2.401944 11.273839
C 9.093931 -0.498813 12.634905
O 8.174775 -1.403974 12.972641
O 9.094135 0.625053 13.081857
H 15.522580 -7.368245 11.094648
H 14.199896 -7.102067 9.015004
H 12.634595 -5.209232 8.778385
H 13.718207 -3.819602 12.680832
H 15.274752 -5.715375 12.926354
H 12.547624 -2.310991 10.872008
H 11.757751 -3.153230 9.542255
H 11.101991 -3.460569 12.508542
H 9.751393 -7.446807 12.828068
H 8.896810 -6.968157 11.347044
H 11.836509 -7.853117 11.471713
H 10.494679 -8.877372 10.934945
H 10.947502 -7.446473 9.997704
H 9.488311 -1.011341 8.889382
H 10.079459 1.971444 8.698426
H 11.045947 0.680923 7.973951
H 9.390051 0.962371 7.422469
H 7.643684 -0.386419 10.443043
H 7.443214 0.410203 8.890830
H 8.067692 1.318653 10.272356
H 11.486380 -0.314855 10.149376
H 10.576599 0.934663 10.975421
H 11.063767 -1.104445 12.310523
H 8.438044 -2.209378 12.445380
H 9.452221 -2.432312 10.323180

1a-C6, ΔG = 4.6116 kcal/mol, population = 0.02 %
C 12.881744 -6.130006 7.885250
C 13.125627 -6.504293 9.203317
C 13.180372 -5.539061 10.202900
C 12.990167 -4.187431 9.906004
C 12.757075 -3.822372 8.578013
C 12.700357 -4.785295 7.575332
C 13.000773 -3.159009 11.006772
C 11.589575 -2.823745 11.565837
C 10.968062 -4.104985 12.112088

O 11.532045 -4.460853 13.268981
O 10.122269 -4.747042 11.532384
C 11.132805 -5.736142 13.854809
C 9.854820 -5.603632 14.654784
C 7.769123 -2.451041 11.982876
C 6.717271 -1.861327 11.039762
C 7.129149 -2.841132 13.317331
C 8.927906 -1.476015 12.235717
C 9.791112 -1.168809 11.009654
N 10.755640 -2.183730 10.554974
C 10.539516 0.171135 11.139589
O 10.149054 1.121669 11.774775
O 11.665455 0.219773 10.417505
H 12.840074 -6.879297 7.104789
H 13.277172 -7.547091 9.452945
H 13.376827 -5.837565 11.226090
H 12.619920 -2.778008 8.327289
H 12.519545 -4.484537 6.550810
H 13.613232 -3.510269 11.837412
H 13.435915 -2.223919 10.652670
H 11.734798 -2.133407 12.396510
H 11.032990 -6.465698 13.052788
H 11.976018 -6.006618 14.485778
H 9.641500 -6.553038 15.149741
H 9.954662 -4.833832 15.421257
H 9.011957 -5.356398 14.011009
H 8.173159 -3.357056 11.525931
H 7.122492 -1.629180 10.052796
H 5.896338 -2.566333 10.892964
H 6.298308 -0.940311 11.455579
H 7.863446 -3.281085 13.994599
H 6.326946 -3.567429 13.170689
H 6.700745 -1.964313 13.811835
H 8.520683 -0.530272 12.599449
H 9.561405 -1.857269 13.040814
H 9.129331 -0.998478 10.155396
H 10.312804 -2.879359 9.968100
H 11.744730 -0.689028 10.030833

1a-C2, ΔG = 4.6172 kcal/mol, population = 0.02 %
C 12.446934 -6.317482 7.918123
C 12.711174 -6.684534 9.234221
C 12.892885 -5.707629 10.207154
C 12.811555 -4.351193 9.885676

C 12.560773 -3.994514 8.558355
C 12.375408 -4.968567 7.582593
C 12.967582 -3.302019 10.955796
C 11.625709 -2.786256 11.542687
C 10.894216 -3.946177 12.210659
O 11.368234 -4.177458 13.438509
O 10.023155 -4.593522 11.676350
C 10.806749 -5.300458 14.181308
C 11.476446 -6.604924 13.804600
C 7.830797 -2.193208 11.994873
C 6.756303 -1.484318 11.166365
C 7.263504 -2.636965 13.344613
C 9.059718 -1.297804 12.214669
C 9.904957 -1.060659 10.960488
N 10.819683 -2.128172 10.520552
C 10.707903 0.252397 11.030209
O 10.358880 1.242317 11.628552
O 11.829107 0.226589 10.300882
H 12.305997 -7.076073 7.158552
H 12.779008 -7.731249 9.503479
H 13.104584 -6.001891 11.227741
H 12.512275 -2.947692 8.286691
H 12.181782 -4.673690 6.558711
H 13.562251 -3.696360 11.780468
H 13.495061 -2.432779 10.560760
H 11.878313 -2.058368 12.313265
H 10.988866 -5.041164 15.221355
H 9.733826 -5.330439 13.999347
H 11.093629 -7.402144 14.444604
H 11.269702 -6.870393 12.768406
H 12.556116 -6.541797 13.947274
H 8.148325 -3.088517 11.455419
H 6.403285 -0.585556 11.680214
H 7.120539 -1.184138 10.181484
H 5.897588 -2.139992 11.007865
H 6.954798 -1.771681 13.938541
H 8.004993 -3.192230 13.923038
H 6.390768 -3.279554 13.210853
H 8.728300 -0.325210 12.583555
H 9.690637 -1.715283 13.003263
H 9.229752 -0.893651 10.116153
H 10.343083 -2.812896 9.947458
H 11.867950 -0.699862 9.950306

1a-C91, ΔG = 4.7320 kcal/mol, population = 0.01 %
C 15.203027 -6.085283 9.768413
C 13.981962 -6.352632 9.157512
C 12.935412 -5.441069 9.260372
C 13.093667 -4.250537 9.971610
C 14.324177 -3.991134 10.579428
C 15.371562 -4.900071 10.479931
C 11.943598 -3.290877 10.132288
C 11.247196 -3.433605 11.505865
C 10.824045 -4.876332 11.745249
O 9.861281 -5.233873 10.887615
O 11.301284 -5.602106 12.585859
C 9.321556 -6.583422 10.988639
C 8.225291 -6.654322 12.030445
C 8.534944 -0.657519 9.844555
C 8.361345 0.154909 8.558185
C 7.377233 -0.392097 10.809323
C 9.914025 -0.355046 10.447912
C 10.302957 -1.131294 11.716425
N 10.066239 -2.580687 11.673113
C 9.645301 -0.560040 12.987497
O 9.621719 0.618227 13.259184
O 9.141455 -1.491782 13.798010
H 16.018426 -6.793132 9.689076
H 13.843387 -7.270094 8.599108
H 11.985546 -5.655331 8.785132
H 14.463303 -3.068897 11.131925
H 16.320375 -4.682458 10.954387
H 12.298834 -2.263321 10.041401
H 11.200452 -3.454908 9.350665
H 11.954288 -3.191702 12.298312
H 8.942680 -6.789959 9.990569
H 10.139966 -7.265106 11.212805
H 7.430371 -5.942119 11.805187
H 7.795166 -7.657674 12.033497
H 8.614906 -6.446508 13.026942
H 8.503180 -1.715027 9.556740
H 7.411640 -0.080573 8.074135
H 8.371487 1.226571 8.775461
H 9.164373 -0.051798 7.847441
H 7.387293 0.645607 11.150980
H 7.408918 -1.035153 11.690340
H 6.422019 -0.574785 10.313346
H 10.672058 -0.555393 9.688506

H 9.978152 0.709384 10.684370
H 11.370415 -0.964870 11.884593
H 9.304066 -2.343473 13.305957
H 9.368451 -2.823281 10.979480

1a-C65, ΔG = 4.7402 kcal/mol, population = 0.01 %
C 14.777442 -3.569203 8.586248
C 14.780349 -3.762189 9.966335
C 13.687700 -4.350279 10.590578
C 12.573961 -4.755219 9.849726
C 12.583983 -4.563899 8.467972
C 13.677988 -3.974401 7.838294
C 11.363204 -5.322521 10.546863
C 10.619334 -4.223929 11.335939
C 9.450479 -4.824135 12.113039
O 9.858362 -5.698279 13.022905
O 8.282282 -4.533820 11.931166
C 8.839521 -6.355056 13.835442
C 9.550662 -7.295263 14.777419
C 11.716621 -0.140438 11.909669
C 12.396371 -1.088157 12.901691
C 10.676970 0.740420 12.606610
C 11.141824 -0.893447 10.696602
C 9.974589 -1.852045 11.003885
N 10.127710 -3.196620 10.444065
C 8.604877 -1.295538 10.578490
O 8.401214 -0.147850 10.253565
O 7.601912 -2.177108 10.628501
H 15.627553 -3.107978 8.099812
H 15.635146 -3.453892 10.555231
H 13.696991 -4.494518 11.664663
H 11.730528 -4.879142 7.878883
H 13.667885 -3.831654 6.765015
H 10.678003 -5.761785 9.819189
H 11.653472 -6.106696 11.244990
H 11.316392 -3.829670 12.086710
H 8.280319 -5.584438 14.365496
H 8.158668 -6.880123 13.165971
H 10.233755 -6.751671 15.431148
H 8.813980 -7.805933 15.399624
H 10.114710 -8.048387 14.225906
H 12.492405 0.520327 11.508875
H 11.677147 -1.762943 13.374381
H 13.154879 -1.699460 12.407882

H 12.881719 -0.526238 13.702422
H 11.142038 1.322412 13.405233
H 9.883274 0.141191 13.061407
H 10.208449 1.431218 11.904507
H 10.817002 -0.169996 9.948092
H 11.947069 -1.481361 10.250823
H 9.880249 -1.966846 12.087959
H 7.940337 -3.066425 10.898069
H 10.674708 -3.163723 9.594969

1a-C99, ΔG = 4.7440 kcal/mol, population = 0.01 %
C 14.690135 -6.481502 10.714605
C 13.764026 -6.589840 9.683163
C 12.835085 -5.575677 9.470274
C 12.819768 -4.440375 10.280200
C 13.758467 -4.338214 11.310182
C 14.685791 -5.350234 11.527669
C 11.776012 -3.370567 10.086061
C 10.697675 -3.394502 11.193869
C 10.000216 -4.746874 11.179761
O 10.330489 -5.490104 12.235327
O 9.244128 -5.090213 10.297819
C 9.794290 -6.845305 12.309536
C 10.639979 -7.809324 11.504939
C 9.684675 0.432536 9.208176
C 8.825551 1.590805 8.693283
C 9.666925 -0.727582 8.210786
C 9.216281 0.048459 10.620192
C 10.078025 -0.993715 11.358868
N 9.660869 -2.365671 11.062188
C 9.921379 -0.762078 12.869666
O 10.459104 0.138397 13.470795
O 9.095762 -1.625627 13.467619
H 15.412114 -7.270326 10.884147
H 13.759725 -7.465437 9.046134
H 12.110030 -5.668891 8.670641
H 13.763149 -3.460243 11.946053
H 15.407500 -5.256150 12.329552
H 12.243337 -2.384524 10.084541
H 11.281128 -3.505594 9.123587
H 11.177711 -3.269992 12.163957
H 9.820697 -7.076476 13.371616
H 8.760616 -6.828351 11.968759
H 11.683586 -7.761313 11.816922

H 10.275896 -8.825907 11.665581
H 10.586035 -7.588125 10.439788
H 10.717123 0.788934 9.294791
H 7.781159 1.281901 8.592647
H 8.859607 2.444600 9.373354
H 9.171073 1.924414 7.712866
H 10.379298 -1.508684 8.472460
H 9.931842 -0.373289 7.212668
H 8.670611 -1.175687 8.147175
H 9.208090 0.957442 11.223623
H 8.183490 -0.314919 10.588573
H 11.130960 -0.808945 11.126833
H 8.838946 -2.258510 12.748869
H 9.185397 -2.444454 10.173106

1a-C16, ΔG = 4.7578 kcal/mol, population = 0.01 %
C 14.297795 -5.023346 9.119258
C 13.737466 -5.963601 9.980332
C 13.134414 -5.556492 11.164603
C 13.078380 -4.202737 11.510090
C 13.655983 -3.270782 10.644625
C 14.259608 -3.674863 9.456996
C 12.351965 -3.748407 12.750065
C 10.822597 -3.633313 12.524695
C 10.199695 -5.028679 12.412465
O 9.386886 -5.161444 11.362293
O 10.416935 -5.903999 13.221217
C 8.747317 -6.455671 11.159065
C 9.656528 -7.392414 10.391620
C 10.127848 -0.345528 9.291963
C 10.380935 -1.629230 8.493596
C 10.697747 0.862241 8.541402
C 10.706830 -0.362952 10.716337
C 10.130100 -1.378669 11.718226
N 10.464114 -2.767041 11.412933
C 8.599159 -1.238671 11.774835
O 7.940467 -2.308006 11.323778
O 8.029888 -0.247741 12.173000
H 14.766213 -5.340901 8.196306
H 13.771746 -7.016533 9.729783
H 12.706976 -6.295322 11.830322
H 13.641208 -2.219390 10.906285
H 14.700764 -2.936275 8.799506
H 12.523993 -4.438511 13.575349

H 12.714109 -2.766296 13.055810
H 10.387132 -3.217862 13.437273
H 8.469677 -6.862956 12.129359
H 7.848658 -6.216639 10.595523
H 10.546437 -7.640604 10.969144
H 9.119774 -8.317838 10.174460
H 9.964021 -6.943676 9.446270
H 9.044207 -0.203984 9.369989
H 11.449205 -1.864105 8.457607
H 9.857567 -2.489153 8.910730
H 10.036984 -1.506680 7.464703
H 11.782747 0.773213 8.434687
H 10.487047 1.794328 9.069802
H 10.268737 0.934850 7.540207
H 11.788810 -0.516640 10.671825
H 10.551392 0.624021 11.154738
H 10.498318 -1.097053 12.709034
H 8.651655 -2.967219 11.098693
H 11.129105 -2.848644 10.656946

1a-C87, ΔG = 4.7584 kcal/mol, population = 0.01 %
C 12.668520 -5.780468 7.646257
C 12.826571 -4.397548 7.646412
C 13.028768 -3.718517 8.843412
C 13.073152 -4.406246 10.057756
C 12.926923 -5.795079 10.043776
C 12.724275 -6.478100 8.848970
C 13.218192 -3.666293 11.362882
C 11.863352 -3.235884 11.977292
C 11.044623 -4.484112 12.282342
O 10.034901 -4.670400 11.436149
O 11.328371 -5.229777 13.193826
C 9.181064 -5.835140 11.625931
C 8.085690 -5.539997 12.628655
C 10.591569 0.410538 12.815202
C 12.120849 0.355706 12.736909
C 10.086647 -0.150142 14.146862
C 9.869413 -0.216454 11.607667
C 9.896392 -1.749482 11.495279
N 11.197162 -2.293896 11.086622
C 8.838507 -2.181565 10.463760
O 7.649430 -2.005898 10.614172
O 9.343509 -2.732096 9.361949
H 12.510696 -6.310717 6.715586

H 12.793832 -3.846988 6.714505
H 13.156036 -2.642948 8.833051
H 12.969520 -6.346074 10.975806
H 12.611942 -7.555144 8.857642
H 13.731384 -4.284856 12.098720
H 13.816580 -2.763269 11.226420
H 12.076217 -2.795009 12.958290
H 8.779283 -6.026113 10.633956
H 9.802890 -6.673571 11.933284
H 7.419028 -6.401466 12.698912
H 8.500416 -5.346655 13.617992
H 7.498403 -4.676134 12.314991
H 10.314160 1.469297 12.781306
H 12.560442 0.990818 13.508682
H 12.506451 -0.652156 12.900647
H 12.485345 0.709441 11.769505
H 10.390471 -1.190481 14.286634
H 8.996796 -0.108668 14.207914
H 10.494296 0.421420 14.982826
H 8.819276 0.076579 11.653849
H 10.269911 0.207874 10.680794
H 9.583108 -2.180158 12.446740
H 10.309319 -2.846555 9.574607
H 11.838885 -1.548897 10.850591

1a-C64, ΔG = 4.8707 kcal/mol, population = 0.01 %
C 12.957741 -5.423013 7.773588
C 12.778859 -6.378917 8.768833
C 12.852107 -6.013092 10.108911
C 13.100406 -4.688986 10.476932
C 13.290836 -3.741084 9.469505
C 13.218689 -4.102553 8.128294
C 13.107460 -4.291099 11.930642
C 11.718927 -3.847961 12.455156
C 10.744009 -5.007146 12.290593
O 9.902610 -4.842058 11.272292
O 10.777816 -5.983648 13.006767
C 8.930606 -5.892490 11.002880
C 7.682763 -5.694373 11.837174
C 10.433033 0.555772 11.946743
C 11.746078 0.455777 11.163822
C 10.413170 1.836639 12.785990
C 10.149977 -0.647074 12.861417
C 10.022369 -2.034286 12.202938

N 11.297297 -2.628599 11.772372
C 9.063575 -1.973605 11.004727
O 7.903781 -1.637440 11.096761
O 9.622567 -2.293142 9.838792
H 12.901341 -5.705694 6.729948
H 12.584621 -7.410500 8.502713
H 12.711116 -6.763196 10.878005
H 13.499916 -2.712157 9.734678
H 13.367925 -3.353442 7.360670
H 13.442699 -5.122434 12.550499
H 13.799944 -3.463901 12.099011
H 11.808030 -3.696652 13.537164
H 8.725078 -5.790846 9.940094
H 9.400448 -6.855706 11.191232
H 7.249446 -4.710187 11.655270
H 6.943417 -6.450599 11.566886
H 7.901370 -5.794113 12.900463
H 9.614727 0.622700 11.222215
H 11.924286 1.377737 10.607101
H 12.596514 0.311680 11.837664
H 11.736867 -0.357603 10.437459
H 11.219922 1.826836 13.524485
H 9.467868 1.945269 13.321736
H 10.548398 2.716978 12.154656
H 10.927016 -0.720431 13.628746
H 9.211899 -0.455305 13.384680
H 9.544106 -2.690147 12.932262
H 10.525806 -2.626808 10.088914
H 12.044332 -1.951094 11.844103

1a-C20, ΔG = 4.8726 kcal/mol, population = 0.01 %
C 14.723230 -3.585708 8.500745
C 14.788755 -3.736115 9.884573
C 13.732335 -4.317625 10.573919
C 12.592595 -4.757637 9.895275
C 12.540374 -4.609899 8.509135
C 13.598130 -4.027355 7.814271
C 11.421159 -5.317536 10.662046
C 10.700588 -4.204181 11.452536
C 9.585856 -4.802472 12.306612
O 10.069358 -5.593242 13.257523
O 8.401606 -4.576467 12.143156
C 9.123054 -6.279051 14.132703
C 8.639155 -7.566773 13.501358

C 11.774666 -0.107336 11.801675
C 12.542954 -1.034588 12.747068
C 10.784530 0.772656 12.567759
C 11.109384 -0.881087 10.649667
C 9.989958 -1.856808 11.066741
N 10.144438 -3.218199 10.552441
C 8.583041 -1.346117 10.709106
O 8.327416 -0.204054 10.401191
O 7.612180 -2.258837 10.804606
H 15.545275 -3.129784 7.963696
H 15.664355 -3.399801 10.425639
H 13.790565 -4.428773 11.650410
H 11.666331 -4.953373 7.968021
H 13.539693 -3.918735 6.738538
H 10.709107 -5.784456 9.978825
H 11.752044 -6.078186 11.368153
H 11.428397 -3.772426 12.150583
H 9.694142 -6.463691 15.039147
H 8.301931 -5.599650 14.352564
H 7.990585 -8.090831 14.205844
H 8.069148 -7.370551 12.593230
H 9.479440 -8.218625 13.259137
H 12.507895 0.555187 11.329845
H 13.090176 -0.456854 13.494855
H 11.870664 -1.705257 13.289691
H 13.260471 -1.650250 12.200372
H 11.304776 1.371122 13.318689
H 10.036480 0.171703 13.092202
H 10.253314 1.447868 11.895644
H 10.706693 -0.167868 9.929858
H 11.881776 -1.458146 10.136801
H 9.964142 -1.928551 12.158921
H 7.993143 -3.135463 11.056140
H 10.640843 -3.209343 9.672319

1a-C51, ΔG = 4.8852 kcal/mol, population = 0.01 %
C 12.822982 -6.561364 8.490307
C 13.478430 -6.417063 9.709705
C 13.535070 -5.172528 10.326788
C 12.935737 -4.054357 9.742598
C 12.293306 -4.207608 8.512231
C 12.234456 -5.451506 7.891235
C 12.944806 -2.728753 10.458401
C 11.668181 -2.458082 11.306225

C 11.585191 -3.551934 12.367733
O 10.806008 -4.564804 11.981150
O 12.230504 -3.533386 13.392234
C 10.825098 -5.788477 12.773664
C 9.793700 -5.756674 13.880196
C 8.179178 -2.488213 12.813331
C 6.780872 -2.428022 12.193914
C 8.088390 -2.507138 14.340361
C 9.057937 -1.314267 12.359824
C 9.455135 -1.349333 10.882296
N 10.486632 -2.311089 10.459424
C 9.852367 0.042409 10.353755
O 10.721724 -0.005581 9.338209
O 9.399762 1.087279 10.757745
H 12.777946 -7.529138 8.006909
H 13.947676 -7.272751 10.179050
H 14.046999 -5.066954 11.276217
H 11.835320 -3.348744 8.037969
H 11.731959 -5.552733 6.937256
H 13.804169 -2.673422 11.127662
H 13.032801 -1.907412 9.746409
H 11.838639 -1.521891 11.835432
H 10.613850 -6.569805 12.047498
H 11.831273 -5.923728 13.165393
H 9.811560 -6.711418 14.409337
H 10.006895 -4.964530 14.596881
H 8.791199 -5.608752 13.478443
H 8.652226 -3.417851 12.489904
H 6.267558 -1.509372 12.491865
H 6.807167 -2.457749 11.102783
H 6.176227 -3.274091 12.526844
H 7.621930 -1.589204 14.709457
H 9.077200 -2.586913 14.796884
H 7.488213 -3.350472 14.687735
H 8.523827 -0.379217 12.538781
H 9.958932 -1.267615 12.976518
H 8.568663 -1.596332 10.291192
H 10.090466 -3.210930 10.225435
H 10.927963 -0.970734 9.244579

1a-C60, ΔG = 5.0872 kcal/mol, population = 0.01 %
C 14.960702 -3.272555 8.682917
C 13.763110 -3.221745 7.977555
C 12.626064 -3.842823 8.487111

C 12.667775 -4.512078 9.711677
C 13.878703 -4.565616 10.405870
C 15.016898 -3.952672 9.896853
C 11.426994 -5.125070 10.304246
C 10.704149 -4.169251 11.288288
C 9.571164 -4.958268 11.931135
O 10.016442 -5.761390 12.886797
O 8.404079 -4.890327 11.588639
C 9.050953 -6.618296 13.572302
C 8.373881 -5.872970 14.702338
C 11.676645 -0.835448 12.388424
C 11.952535 0.457679 13.161561
C 12.907503 -1.224989 11.567468
C 10.414564 -0.674418 11.518021
C 9.566839 -1.934782 11.342940
N 10.167951 -3.029710 10.576759
C 8.199544 -1.593865 10.710154
O 7.438199 -2.643646 10.394292
O 7.800946 -0.467183 10.514542
H 15.846138 -2.791267 8.287461
H 13.712648 -2.702623 7.028688
H 11.699392 -3.810355 7.925916
H 13.926124 -5.086013 11.355385
H 15.947625 -4.002748 10.447926
H 10.720867 -5.394074 9.516763
H 11.687051 -6.032803 10.848059
H 11.407701 -3.904570 12.087484
H 8.335217 -6.986078 12.839806
H 9.653831 -7.445651 13.937836
H 7.713768 -6.557908 15.237742
H 9.110545 -5.486282 15.407598
H 7.774000 -5.044787 14.325513
H 11.486846 -1.625404 13.123828
H 11.117018 0.715364 13.816021
H 12.850185 0.363169 13.776999
H 12.107916 1.291658 12.470781
H 13.775767 -1.376719 12.212298
H 13.153092 -0.431042 10.856542
H 12.756453 -2.140306 11.000961
H 9.763467 0.076972 11.965714
H 10.688270 -0.283343 10.532602
H 9.325434 -2.318960 12.340246
H 7.894405 -3.486265 10.639295
H 10.815851 -2.685493 9.882200

1a-C68, ΔG = 5.1349 kcal/mol, population = 0.01 %

C 12.588173 -6.017137 7.853600
C 12.617768 -4.625875 7.804457
C 12.811461 -3.889670 8.968146
C 12.972363 -4.529037 10.199997
C 12.952858 -5.924243 10.234332
C 12.761757 -6.664486 9.072186
C 13.117888 -3.741856 11.477713
C 11.768914 -3.294306 12.088855
C 10.917269 -4.528149 12.367303
O 9.873100 -4.642815 11.551020
O 11.195529 -5.313272 13.246767
C 8.981012 -5.782952 11.721895
C 9.482006 -6.990651 10.960054
C 10.703588 0.364221 13.057629
C 10.345815 1.839820 13.263182
C 12.223825 0.208044 12.958872
C 9.938928 -0.172924 11.836132
C 9.876972 -1.701105 11.684028
N 11.128398 -2.314883 11.218675
C 8.757879 -2.050118 10.684766
O 7.588887 -1.799009 10.878346
O 9.186649 -2.617240 9.559196
H 12.437705 -6.590873 6.947817
H 12.493715 -4.113472 6.858550
H 12.842402 -2.808683 8.916653
H 13.083011 -6.434296 11.181060
H 12.745564 -7.746114 9.120270
H 13.635615 -4.338141 12.228151
H 13.716703 -2.843657 11.311518
H 11.982275 -2.878054 13.080931
H 8.881944 -5.986303 12.786561
H 8.030877 -5.427343 11.330448
H 8.739303 -7.788023 11.027656
H 9.634601 -6.748991 9.907957
H 10.420253 -7.357759 11.373284
H 10.357229 -0.192390 13.935691
H 9.270242 1.973391 13.395774
H 10.849530 2.243205 14.143912
H 10.655055 2.435797 12.399575
H 12.541478 -0.833426 12.963473
H 12.710255 0.693076 13.807513
H 12.603678 0.678127 12.047081

H 8.907169 0.175036 11.901560
H 10.354579 0.260079 10.918664
H 9.576970 -2.131465 12.641785
H 10.149388 -2.796768 9.733928
H 11.802509 -1.602399 10.971236

1a-C81, ΔG = 5.1650 kcal/mol, population = 0.01 %
C 13.117252 -5.547751 8.024358
C 13.276782 -4.203483 8.350545
C 13.173713 -3.787642 9.673431
C 12.903463 -4.705186 10.692103
C 12.762796 -6.051955 10.354194
C 12.865322 -6.472812 9.032088
C 12.720457 -4.261849 12.121401
C 11.305931 -3.711321 12.426493
C 10.244145 -4.734099 12.042008
O 10.276543 -5.779247 12.875296
O 9.473069 -4.618060 11.118094
C 9.304928 -6.845258 12.660001
C 9.792147 -7.849175 11.637074
C 10.560367 0.801272 11.851524
C 11.931161 0.563998 11.209663
C 10.593732 2.062356 12.719639
C 10.046147 -0.374958 12.697510
C 9.848431 -1.732980 11.997108
N 11.094303 -2.460070 11.707624
C 9.048585 -1.561308 10.697393
O 9.687595 -1.970721 9.603299
O 7.941650 -1.071415 10.656763
H 13.196301 -5.871229 6.994135
H 13.484498 -3.477805 7.574031
H 13.309006 -2.741233 9.914707
H 12.570647 -6.777257 11.134362
H 12.750089 -7.522278 8.791167
H 12.905380 -5.095015 12.797959
H 13.434944 -3.476091 12.377114
H 11.234393 -3.573662 13.512278
H 9.192141 -7.297274 13.642575
H 8.361293 -6.392823 12.360567
H 10.739017 -8.293357 11.946178
H 9.055299 -8.649464 11.544580
H 9.921335 -7.386460 10.659310
H 9.838788 0.972663 11.046218
H 12.682572 0.314345 11.965328

H 11.910611 -0.231565 10.463851
H 12.270451 1.467729 10.699855
H 9.614400 2.269830 13.155901
H 10.893628 2.931619 12.131113
H 11.310103 1.948900 13.538401
H 10.716854 -0.540360 13.546687
H 9.080150 -0.087837 13.115925
H 9.221331 -2.341729 12.650045
H 10.508537 -2.408188 9.955966
H 11.894390 -1.863638 11.867314

1a-C28, ΔG = 5.5321 kcal/mol, population = 0.00 %
C 12.415287 -6.734856 8.518593
C 13.123554 -6.633549 9.711882
C 13.316831 -5.390538 10.304655
C 12.805865 -4.230371 9.720276
C 12.109073 -4.342110 8.514074
C 11.911146 -5.583798 7.919379
C 12.982834 -2.894275 10.393823
C 11.765876 -2.445271 11.246511
C 11.613228 -3.407362 12.424023
O 10.660587 -4.317517 12.219572
O 12.317836 -3.359045 13.408521
C 10.414260 -5.305418 13.262923
C 11.290049 -6.525939 13.084587
C 8.134810 -2.336132 12.682498
C 6.748052 -2.007498 12.123940
C 8.075066 -2.514801 14.200374
C 9.161395 -1.252859 12.317953
C 9.586936 -1.263277 10.847202
N 10.581099 -2.258474 10.414566
C 10.055244 0.120416 10.356729
O 9.653928 1.175603 10.786772
O 10.923781 0.056506 9.341067
H 12.263851 -7.701494 8.054978
H 13.526028 -7.522016 10.182134
H 13.870609 -5.319265 11.233054
H 11.722615 -3.451013 8.035789
H 11.368823 -5.651774 6.984472
H 13.854842 -2.919481 11.048122
H 13.154925 -2.114194 9.651323
H 12.033392 -1.483814 11.685721
H 10.574299 -4.831019 14.228931
H 9.359862 -5.546955 13.150268

H 11.014434 -7.273270 13.831199
H 11.155350 -6.959898 12.093967
H 12.342765 -6.279377 13.218379
H 8.462924 -3.281469 12.244067
H 6.038756 -2.804391 12.356955
H 6.367611 -1.079795 12.560634
H 6.756071 -1.885795 11.038602
H 9.051593 -2.784942 14.607793
H 7.365788 -3.297780 14.476442
H 7.756883 -1.587737 14.685949
H 8.737640 -0.271686 12.539992
H 10.046550 -1.344818 12.951824
H 8.701346 -1.456225 10.234041
H 10.150549 -3.143063 10.182965
H 11.084653 -0.913773 9.220385

1a-C90, ΔG = 5.5478 kcal/mol, population = 0.00 %
C 15.066722 -3.378955 9.069032
C 13.944178 -3.417802 8.249919
C 12.773828 -4.023927 8.699190
C 12.710292 -4.599044 9.969326
C 13.845426 -4.556699 10.782644
C 15.014390 -3.952412 10.337391
C 11.432174 -5.221327 10.469577
C 10.707107 -4.329748 11.502592
C 9.446487 -5.057858 11.953989
O 9.739384 -5.965151 12.889789
O 8.340702 -4.866478 11.502213
C 8.650410 -6.790097 13.398502
C 7.911043 -6.084180 14.515692
C 11.668979 -0.178133 11.441173
C 10.933863 0.359292 10.210696
C 12.964675 -0.893436 11.040498
C 10.775015 -1.001064 12.396469
C 9.802659 -2.039329 11.815013
N 10.355218 -3.050683 10.903171
C 8.562872 -1.406383 11.156429
O 7.954305 -0.471511 11.626528
O 8.177792 -2.016007 10.036655
H 15.977322 -2.906621 8.723138
H 13.975709 -2.975243 7.262293
H 11.901012 -4.051353 8.057663
H 13.812692 -5.001178 11.770511
H 15.885947 -3.928002 10.979457

H 10.752811 -5.400182 9.634561
H 11.640087 -6.181314 10.943145
H 11.355053 -4.213053 12.378412
H 7.989372 -7.040898 12.570823
H 9.146116 -7.691507 13.750850
H 7.150059 -6.752495 14.923180
H 8.594769 -5.812766 15.321158
H 7.416294 -5.183518 14.152445
H 11.974395 0.689626 12.034382
H 10.048011 0.931731 10.487566
H 11.592126 1.012402 9.634341
H 10.618216 -0.445639 9.541079
H 12.808434 -1.699875 10.322933
H 13.466554 -1.320798 11.910157
H 13.652006 -0.188403 10.568049
H 11.418817 -1.515759 13.113577
H 10.157922 -0.312751 12.976418
H 9.378536 -2.567557 12.673348
H 8.838321 -2.754348 9.929246
H 11.154882 -2.688101 10.401193

1a-C80, ΔG = 5.6476 kcal/mol, population = 0.00 %
C 12.395192 -6.022629 7.808697
C 12.438522 -4.635246 7.698665
C 12.680402 -3.852537 8.822351
C 12.876170 -4.440685 10.074383
C 12.842885 -5.832738 10.169393
C 12.603545 -6.619490 9.047488
C 13.074399 -3.601685 11.311172
C 11.751695 -3.117181 11.951332
C 10.903960 -4.329827 12.319945
O 9.828000 -4.473743 11.550840
O 11.210938 -5.075080 13.224168
C 8.936791 -5.598296 11.807481
C 9.398998 -6.840795 11.077780
C 10.778647 0.606993 12.736736
C 12.290822 0.438231 12.560292
C 10.329282 0.128210 14.119173
C 9.932562 -0.001587 11.602189
C 9.849983 -1.535366 11.530794
N 11.084287 -2.176890 11.059393
C 8.700883 -1.918818 10.581096
O 9.092286 -2.546278 9.473979
O 7.540209 -1.645676 10.794333

H 12.207450 -6.632664 6.934134
H 12.287593 -4.162155 6.736332
H 12.721598 -2.775030 8.723818
H 13.001541 -6.303689 11.131905
H 12.577282 -7.697796 9.142710
H 13.616016 -4.169069 12.067201
H 13.671930 -2.715916 11.084300
H 12.007625 -2.656654 12.913245
H 8.880529 -5.755038 12.883102
H 7.973458 -5.252331 11.440345
H 10.350602 -7.198281 11.468476
H 8.654313 -7.628578 11.207303
H 9.511521 -6.643458 10.011503
H 10.574616 1.681527 12.682878
H 12.614826 0.733773 11.559541
H 12.825547 1.062544 13.278961
H 12.612049 -0.589785 12.735421
H 9.251338 0.246359 14.250800
H 10.829834 0.699984 14.902974
H 10.573871 -0.924486 14.280876
H 8.910339 0.363586 11.711946
H 10.294494 0.371325 10.638257
H 9.570685 -1.922695 12.511308
H 10.058378 -2.725215 9.630042
H 11.751217 -1.480232 10.754949

1a-C45, ΔG = 5.7072 kcal/mol, population = 0.00 %
C 14.838516 -2.854774 8.431538
C 15.103958 -3.152944 9.766098
C 14.132368 -3.757836 10.554159
C 12.877870 -4.073197 10.026619
C 12.626389 -3.776829 8.687432
C 13.597161 -3.172838 7.893018
C 11.811183 -4.713484 10.883453
C 11.496582 -3.908702 12.151517
C 10.398392 -4.520405 13.030287
O 10.273409 -5.836568 12.864107
O 9.764986 -3.866192 13.829584
C 9.310986 -6.542703 13.704425
C 7.913585 -6.459153 13.127978
C 8.005244 -3.097728 10.988747
C 7.735320 -3.358295 9.505197
C 6.695835 -2.948272 11.762855
C 8.887307 -1.855606 11.187710

C 10.360808 -2.067783 10.812136
N 11.255377 -2.486117 11.901941
C 10.985574 -0.823471 10.151215
O 12.244469 -0.594580 10.520600
O 10.413711 -0.134385 9.336986
H 15.594030 -2.379723 7.818721
H 16.069619 -2.913228 10.193532
H 14.354222 -3.983977 11.590331
H 11.660848 -4.019643 8.259027
H 13.379530 -2.947900 6.856423
H 10.907135 -4.866930 10.293589
H 12.131799 -5.705652 11.201257
H 12.383563 -3.949173 12.793152
H 9.679952 -7.565372 13.716138
H 9.360008 -6.127696 14.709543
H 7.896785 -6.820622 12.098863
H 7.243754 -7.084612 13.721134
H 7.535965 -5.438145 13.150817
H 8.528187 -3.969139 11.391017
H 8.657302 -3.459526 8.928392
H 7.161469 -4.277365 9.371082
H 7.162334 -2.534421 9.071202
H 6.050006 -3.816505 11.615902
H 6.148269 -2.063435 11.425794
H 6.883257 -2.840743 12.833267
H 8.481695 -1.043491 10.581342
H 8.842821 -1.529456 12.229601
H 10.415086 -2.817094 10.023317
H 12.425612 -1.312636 11.189785
H 10.959093 -2.055372 12.771775

1a-C63, ΔG = 5.7988 kcal/mol, population = 0.00 %
C 13.783070 -5.167346 8.067736
C 13.949296 -3.844963 8.464374
C 13.556776 -3.445344 9.738526
C 12.986922 -4.354548 10.632132
C 12.838246 -5.683353 10.226435
C 13.230963 -6.087250 8.955787
C 12.517956 -3.900928 11.990940
C 10.999381 -3.589315 12.056964
C 10.203289 -4.890425 11.921334
O 10.377370 -5.672299 12.987570
O 9.521350 -5.182747 10.963569
C 9.688296 -6.957523 13.013007

C 8.267994 -6.803225 13.514027
C 11.031079 0.573192 12.049919
C 12.290107 -0.186992 12.466884
C 10.201820 0.967798 13.274688
C 10.180907 -0.160777 10.999126
C 9.663603 -1.558916 11.395806
N 10.591438 -2.629347 11.052829
C 8.286930 -1.791753 10.742330
O 8.180778 -2.885259 9.992696
O 7.352210 -1.035135 10.894691
H 14.089457 -5.482310 7.078314
H 14.389084 -3.124478 7.786199
H 13.711074 -2.417139 10.045156
H 12.420069 -6.410753 10.911767
H 13.110055 -7.121730 8.659455
H 12.733412 -4.660953 12.741014
H 13.048999 -2.995582 12.281302
H 10.795799 -3.208382 13.062776
H 9.720887 -7.388037 12.013755
H 10.289448 -7.563128 13.686900
H 7.673060 -6.197829 12.830433
H 7.804498 -7.788598 13.591107
H 8.254488 -6.340383 14.501656
H 11.353616 1.499082 11.561869
H 12.915700 0.434250 13.110813
H 12.040288 -1.087548 13.031200
H 12.888842 -0.479881 11.602018
H 9.315345 1.536596 12.986465
H 10.792745 1.582384 13.956796
H 9.869518 0.089854 13.834960
H 9.320437 0.467767 10.770219
H 10.747551 -0.264455 10.068396
H 9.463568 -1.576800 12.471604
H 9.026664 -3.391556 10.081174
H 11.354556 -2.316110 10.473763

1a-C93, ΔG = 5.8816 kcal/mol, population = 0.00 %
C 14.973751 -3.519251 9.066543
C 14.963750 -3.980649 10.380709
C 13.805554 -4.526705 10.919700
C 12.639825 -4.622808 10.155775
C 12.661233 -4.160981 8.838789
C 13.820191 -3.612099 8.296097
C 11.375189 -5.178767 10.757608

C 10.676855 -4.172135 11.701255
C 9.425668 -4.844457 12.252193
O 9.740885 -5.658473 13.263993
O 8.308817 -4.690074 11.813810
C 8.680229 -6.487469 13.822708
C 8.498589 -7.753435 13.012000
C 11.687108 -0.086586 11.252238
C 10.994350 0.335957 9.954122
C 12.972668 -0.870248 10.960478
C 10.751059 -0.785736 12.263765
C 9.770020 -1.859690 11.766253
N 10.316874 -2.966622 10.969897
C 8.555446 -1.274563 11.022340
O 8.164974 -2.001893 9.976672
O 7.966517 -0.276603 11.371966
H 15.875615 -3.091750 8.647233
H 15.859422 -3.913788 10.985463
H 13.804258 -4.883149 11.943223
H 11.764906 -4.232258 8.234168
H 13.819132 -3.257038 7.273266
H 10.673239 -5.450682 9.967472
H 11.596282 -6.078018 11.332958
H 11.344541 -3.963821 12.544567
H 9.019145 -6.701840 14.833376
H 7.765853 -5.898551 13.865184
H 7.745493 -8.383091 13.489548
H 8.162846 -7.527781 11.999712
H 9.431677 -8.315865 12.958563
H 12.000547 0.831226 11.759749
H 11.680761 0.912861 9.331266
H 10.676473 -0.526663 9.361788
H 10.114983 0.951062 10.147620
H 13.688207 -0.235780 10.433110
H 12.809695 -1.744065 10.329107
H 13.444159 -1.215438 11.882220
H 11.364157 -1.233732 13.049322
H 10.135091 -0.027874 12.750834
H 9.317979 -2.293778 12.662024
H 8.810344 -2.761164 9.961936
H 11.111625 -2.660727 10.424146

1a-C54, ΔG = 6.0228 kcal/mol, population = 0.00 %
C 13.692493 -5.402040 8.207146
C 13.923727 -4.070900 8.536721

C 13.524708 -3.579685 9.776453
C 12.882773 -4.405230 10.702155
C 12.670396 -5.744031 10.364580
C 13.068837 -6.239346 9.128597
C 12.407865 -3.855475 12.023258
C 10.912049 -3.446180 12.033606
C 10.026860 -4.694135 11.965565
O 10.108669 -5.402562 13.092314
O 9.342057 -5.000393 11.013971
C 9.326269 -6.630042 13.189333
C 10.044518 -7.798888 12.549262
C 11.010250 0.638635 12.103482
C 12.214305 -0.140658 12.632891
C 10.040561 0.983230 13.237036
C 10.293831 -0.036625 10.921952
C 9.673343 -1.422539 11.187231
N 10.589265 -2.533375 10.958689
C 8.395568 -1.588906 10.336638
O 8.298236 -2.731775 9.663534
O 7.521837 -0.750187 10.287689
H 14.003781 -5.787330 7.244506
H 14.419778 -3.414493 7.832780
H 13.729904 -2.545639 10.029221
H 12.197650 -6.407990 11.076828
H 12.896801 -7.280664 8.886359
H 12.556884 -4.588161 12.815664
H 12.987202 -2.970705 12.284184
H 10.715634 -2.989422 13.008991
H 9.202097 -6.774106 14.259832
H 8.353837 -6.454908 12.732681
H 11.036140 -7.931365 12.984363
H 9.471015 -8.710791 12.725732
H 10.141723 -7.662391 11.472523
H 11.388209 1.584861 11.701484
H 12.912732 -0.395471 11.833326
H 12.756143 0.453132 13.371988
H 11.907303 -1.064292 13.126911
H 9.191703 1.563846 12.869697
H 10.544710 1.569529 14.007933
H 9.648182 0.084375 13.720013
H 9.496181 0.629770 10.594041
H 10.985371 -0.134259 10.078747
H 9.316024 -1.466284 12.220379
H 9.078492 -3.289174 9.909680

H 11.390595 -2.272800 10.405210

1a-C74, ΔG = 6.1352 kcal/mol, population = 0.00 %
C 12.922929 -5.983742 7.850493
C 12.941961 -6.627210 9.083223
C 12.908976 -5.882128 10.257834
C 12.850260 -4.488088 10.223526
C 12.844138 -3.852590 8.979417
C 12.877866 -4.592718 7.803024
C 12.768953 -3.708364 11.512404
C 11.345397 -3.222588 11.884118
C 10.377734 -4.401298 11.832258
O 10.573073 -5.239467 12.841805
O 9.528831 -4.556896 10.974512
C 9.707746 -6.414047 12.938639
C 10.232090 -7.562158 12.104482
C 10.744519 0.073380 13.262064
C 10.378963 1.377896 13.978214
C 12.268629 -0.045134 13.160104
C 10.034872 0.016356 11.895446
C 9.735841 -1.383390 11.356124
N 10.900767 -2.185650 10.979092
C 8.768392 -1.319487 10.154356
O 8.155953 -0.327691 9.825437
O 8.616319 -2.462842 9.484264
H 12.949936 -6.559421 6.933911
H 12.986271 -7.708056 9.133040
H 12.930762 -6.388577 11.214469
H 12.814344 -2.772215 8.926197
H 12.872990 -4.082828 6.847667
H 13.131540 -4.322616 12.335283
H 13.410340 -2.825205 11.468097
H 11.379691 -2.895588 12.930562
H 9.714371 -6.651136 13.999564
H 8.703463 -6.121862 12.638612
H 11.239914 -7.841049 12.413393
H 9.581070 -8.427199 12.244536
H 10.244443 -7.308929 11.045128
H 10.373264 -0.756148 13.874738
H 10.692380 2.242300 13.385466
H 9.301258 1.453309 14.137011
H 10.871891 1.443618 14.950604
H 12.671770 0.767145 12.549057
H 12.592253 -0.985067 12.716502

H 12.726007 0.022737 14.149447
H 9.077715 0.532806 11.972920
H 10.616477 0.572379 11.152225
H 9.184592 -1.933087 12.127521
H 9.154735 -3.178717 9.904172
H 11.664140 -1.596432 10.681655

1a-C89, ΔG = 6.2431 kcal/mol, population = 0.00 %
C 13.987724 -3.122184 8.211672
C 12.765640 -3.423504 7.621909
C 11.748320 -4.004403 8.375073
C 11.933938 -4.288300 9.726623
C 13.173177 -3.995277 10.305322
C 14.190294 -3.414690 9.558815
C 10.843916 -4.916414 10.560646
C 10.340794 -4.017628 11.715732
C 9.685691 -4.850058 12.816823
O 10.503595 -5.804121 13.256623
O 8.575988 -4.639556 13.256246
C 10.018012 -6.686598 14.311823
C 9.221859 -7.836702 13.733166
C 11.215836 -0.929783 12.903276
C 10.316021 0.227614 13.342129
C 12.662935 -0.689563 13.339081
C 11.165058 -1.157407 11.385632
C 9.822319 -1.664737 10.866114
N 9.374322 -2.999236 11.302500
C 9.711767 -1.577173 9.334068
O 10.205892 -0.704415 8.658325
O 8.932892 -2.527692 8.811172
H 14.778059 -2.667127 7.628163
H 12.598939 -3.204041 6.574712
H 10.797673 -4.227443 7.908973
H 13.343971 -4.224094 11.351164
H 15.141261 -3.191790 10.026329
H 9.992378 -5.191748 9.937299
H 11.232084 -5.834251 11.002066
H 11.218052 -3.571184 12.191840
H 10.925800 -7.030858 14.801191
H 9.429564 -6.097305 15.012522
H 9.819675 -8.398969 13.014727
H 8.929075 -8.512655 14.538812
H 8.317191 -7.480719 13.240241
H 10.861298 -1.834120 13.408854

H 10.622385 1.158384 12.856399
H 9.266567 0.051203 13.098386
H 10.378470 0.375600 14.422119
H 13.067851 0.203378 12.854217
H 13.301914 -1.534319 13.071722
H 12.728723 -0.543238 14.419155
H 11.377353 -0.215952 10.875436
H 11.955404 -1.845461 11.083408
H 9.046581 -0.970670 11.202732
H 8.657593 -2.930558 12.013648
H 8.725256 -3.113470 9.588923

1a-C100, ΔG = 6.2914 kcal/mol, population = 0.00 %
C 14.500903 -4.128218 9.494514
C 13.573298 -5.159615 9.612228
C 12.599021 -5.114306 10.602509
C 12.525544 -4.035009 11.487850
C 13.470443 -3.015195 11.366340
C 14.450806 -3.058033 10.380369
C 11.466966 -3.965781 12.558719
C 10.006431 -3.779852 12.051904
C 9.477353 -5.054235 11.408623
O 9.344567 -6.027590 12.312558
O 9.220391 -5.169695 10.231518
C 8.872507 -7.328041 11.850318
C 9.999761 -8.157127 11.272209
C 11.580520 -0.589250 9.383010
C 11.713731 0.643451 8.482552
C 11.565656 -1.859998 8.528138
C 10.341227 -0.416605 10.278427
C 10.220605 -1.354924 11.494430
N 9.763166 -2.686218 11.116693
C 9.178371 -0.742846 12.441841
O 9.405316 0.167598 13.203673
O 7.959098 -1.279431 12.317390
H 15.258532 -4.162296 8.721975
H 13.610670 -6.003645 8.934954
H 11.898467 -5.933416 10.688395
H 13.442095 -2.177822 12.053115
H 15.170398 -2.252773 10.302571
H 11.477568 -4.871671 13.167297
H 11.692490 -3.142010 13.235518
H 9.400245 -3.616503 12.946922
H 8.459428 -7.784946 12.746153

H 8.075466 -7.168322 11.126254
H 10.819242 -8.249604 11.986163
H 9.628130 -9.158660 11.047624
H 10.379040 -7.723195 10.347384
H 12.458784 -0.627419 10.034592
H 10.850502 0.732272 7.816445
H 11.779791 1.561176 9.070806
H 12.608368 0.573791 7.860600
H 10.641283 -1.926807 7.946927
H 11.674221 -2.766674 9.121395
H 12.399830 -1.850337 7.824360
H 10.357083 0.600768 10.673252
H 9.431762 -0.505550 9.674654
H 11.171563 -1.352736 12.031528
H 8.077206 -2.014355 11.663386
H 10.030950 -2.948006 10.179240

1a-C29, ΔG = 6.4031 kcal/mol, population = 0.00 %
C 13.802580 -6.840044 8.929738
C 12.557280 -7.277938 9.366228
C 11.507304 -6.374201 9.500172
C 11.684510 -5.024872 9.196551
C 12.938188 -4.595975 8.753619
C 13.990254 -5.494253 8.621929
C 10.559348 -4.038343 9.377835
C 10.750136 -3.104160 10.593737
C 10.973281 -3.962758 11.835385
O 12.247856 -3.966532 12.226006
O 10.080276 -4.574389 12.379592
C 12.609995 -4.798944 13.368593
C 12.860587 -6.230433 12.945677
C 9.863853 -0.622442 13.895498
C 8.926412 -1.802317 14.162928
C 9.111942 0.708924 13.977080
C 10.640761 -0.754600 12.573079
C 9.763334 -0.902699 11.316073
N 9.577217 -2.238913 10.727173
C 10.183225 0.055035 10.183633
O 9.864197 -0.392851 8.964805
O 10.694487 1.136363 10.353725
H 14.621488 -7.540851 8.827151
H 12.402622 -8.322040 9.607975
H 10.540965 -6.718604 9.848705
H 13.090218 -3.550972 8.508861

H 14.955806 -5.146334 8.276431
H 10.464407 -3.403163 8.495388
H 9.613677 -4.567899 9.504230
H 11.631566 -2.485050 10.434491
H 13.511455 -4.329467 13.754817
H 11.818087 -4.726222 14.112000
H 13.608943 -6.275323 12.154139
H 13.227478 -6.798105 13.803043
H 11.947250 -6.701383 12.585275
H 10.621742 -0.618471 14.685727
H 8.113047 -1.841441 13.432291
H 9.454597 -2.755512 14.124495
H 8.465625 -1.708156 15.148499
H 8.634339 0.822792 14.952421
H 8.323743 0.775710 13.221730
H 9.787726 1.553722 13.828352
H 11.259156 0.134827 12.451309
H 11.330054 -1.595785 12.640287
H 8.753235 -0.557752 11.555202
H 8.817666 -2.742773 11.167888
H 9.488443 -1.295952 9.129704

1a-C83, ΔG = 6.5023 kcal/mol, population = 0.00 %
C 14.991972 -6.451841 10.958836
C 14.150359 -6.545755 9.855977
C 13.233117 -5.533527 9.589805
C 13.146489 -4.413719 10.416561
C 14.001070 -4.325801 11.518594
C 14.915759 -5.336307 11.789874
C 12.114884 -3.345649 10.160129
C 10.947846 -3.386100 11.174926
C 10.279421 -4.751335 11.114939
O 10.562244 -5.494432 12.184463
O 9.585096 -5.105658 10.187470
C 10.051678 -6.861225 12.218443
C 10.973392 -7.803998 11.474654
C 8.637304 0.207672 9.449112
C 7.693529 0.868052 10.457649
C 7.978736 -1.028002 8.825069
C 10.063220 -0.026280 9.989445
C 10.303578 -0.986162 11.169773
N 9.912607 -2.380200 10.921619
C 9.638977 -0.504767 12.469888
O 8.771105 -1.375440 12.989345

O 9.889327 0.555197 12.995772
H 15.704026 -7.239694 11.169855
H 14.202571 -7.409214 9.204618
H 12.572738 -5.615888 8.734837
H 13.949687 -3.459663 12.168331
H 15.571376 -5.253276 12.647787
H 12.575065 -2.357378 10.214087
H 11.696856 -3.458793 9.158352
H 11.333860 -3.236977 12.182304
H 10.006536 -7.095388 13.279261
H 9.045240 -6.865533 11.803985
H 11.988284 -7.745122 11.868481
H 10.613097 -8.827516 11.594868
H 11.000866 -7.570572 10.411203
H 8.785606 0.926084 8.635682
H 7.421013 0.186419 11.265479
H 8.141408 1.757887 10.902551
H 6.766831 1.165484 9.962418
H 7.089613 -0.734662 8.263592
H 7.652222 -1.742888 9.583922
H 8.650596 -1.538728 8.130070
H 10.690867 -0.381597 9.168130
H 10.469798 0.939972 10.294279
H 11.374872 -0.933934 11.381391
H 8.796081 -2.141055 12.352935
H 9.531326 -2.510466 9.994581

1a-C95, ΔG = 6.7369 kcal/mol, population = 0.00 %
C 14.347570 -4.773578 8.954875
C 13.624215 -5.820371 9.520683
C 12.864949 -5.606646 10.665154
C 12.809337 -4.344867 11.263227
C 13.553137 -3.308628 10.696049
C 14.315095 -3.517161 9.550183
C 11.929320 -4.093698 12.459919
C 10.448100 -3.824285 12.075033
C 9.795282 -5.063520 11.468107
O 9.746664 -6.056958 12.363506
O 9.370155 -5.139168 10.339250
C 9.111859 -7.305683 11.958961
C 10.076256 -8.215193 11.227411
C 10.727927 0.049826 9.553866
C 9.346047 0.690695 9.402630
C 10.910592 -1.092785 8.548035

C 11.094689 -0.308322 11.008835
C 10.290479 -1.364791 11.791463
N 10.258491 -2.693641 11.175360
C 8.842201 -0.911028 12.045262
O 8.549376 0.062754 12.700046
O 7.923574 -1.701050 11.484727
H 14.938150 -4.939171 8.062785
H 13.653557 -6.805442 9.071928
H 12.316185 -6.430041 11.102520
H 13.546769 -2.331777 11.162864
H 14.883282 -2.698867 9.125962
H 11.954382 -4.941982 13.143921
H 12.289058 -3.223228 13.010185
H 9.903854 -3.617226 12.998928
H 8.782779 -7.745269 12.897478
H 8.242953 -7.067497 11.347983
H 10.955770 -8.423466 11.838330
H 9.579614 -9.163693 11.014117
H 10.393719 -7.777569 10.281405
H 11.463352 0.817265 9.289053
H 9.204863 1.509601 10.110193
H 9.228416 1.092138 8.393861
H 8.543259 -0.032332 9.555671
H 10.870770 -0.703197 7.528883
H 10.120504 -1.841744 8.634011
H 11.875405 -1.591064 8.671477
H 12.137841 -0.629521 11.028544
H 11.045318 0.606219 11.602984
H 10.748966 -1.408671 12.784158
H 8.463580 -2.403906 11.028680
H 10.869027 -2.765321 10.373934

1a-C92, ΔG = 6.8537 kcal/mol, population = 0.00 %
C 14.172802 -2.943214 8.541573
C 12.996141 -3.108676 7.820632
C 11.908105 -3.762129 8.394605
C 11.978126 -4.254802 9.695962
C 13.173076 -4.097824 10.406015
C 14.260036 -3.446197 9.838123
C 10.805000 -4.942614 10.350138
C 10.205437 -4.146745 11.535597
C 9.490826 -5.060315 12.530944
O 10.265036 -6.075467 12.907241
O 8.371972 -4.857619 12.950914

C 9.746311 -6.988764 13.919946
C 9.999966 -6.453975 15.313267
C 11.053720 -1.119273 12.901179
C 10.111847 -0.015069 13.386200
C 12.487289 -0.857035 13.367733
C 11.031886 -1.269531 11.373318
C 9.701982 -1.748708 10.796724
N 9.251242 -3.109791 11.139476
C 9.628161 -1.569503 9.269871
O 10.105684 -0.637769 8.664414
O 8.906931 -2.517431 8.666425
H 15.017659 -2.431915 8.097798
H 12.919462 -2.726058 6.810554
H 10.993779 -3.879626 7.828272
H 13.251895 -4.487304 11.414677
H 15.174950 -3.330128 10.405594
H 10.015161 -5.137565 9.624100
H 11.138326 -5.906549 10.733134
H 11.041969 -3.730387 12.105025
H 8.686097 -7.146988 13.732381
H 10.288003 -7.913777 13.738429
H 9.455769 -5.525209 15.484388
H 9.662055 -7.188320 16.046929
H 11.064352 -6.276431 15.472290
H 10.722009 -2.058889 13.355371
H 10.166352 0.086956 14.471917
H 10.384946 0.947102 12.943515
H 9.070039 -0.218758 13.131154
H 12.537381 -0.771053 14.455081
H 12.866396 0.075179 12.939341
H 13.156365 -1.663574 13.059596
H 11.252717 -0.302305 10.917661
H 11.829065 -1.941141 11.053244
H 8.916395 -1.078211 11.156805
H 8.506014 -3.085510 11.823924
H 8.690237 -3.148319 9.407498

Table S6. Geometry data of conformers of structure **1b**

1b-C11, ΔG = 0.0000 kcal/mol, population = 60.41 %

C 8.094132 0.469146 -3.160704
C 7.450392 -0.442290 -3.990005
C 6.186957 -0.153051 -4.496770
C 5.551615 1.049056 -4.186708
C 6.209340 1.960573 -3.357087

C 7.469739 1.674157 -2.846365
C 4.161861 1.338310 -4.695188
C 3.092096 1.241231 -3.592653
C 3.201329 -0.118449 -2.902624
O 3.674315 -0.012737 -1.660066
O 2.888067 -1.158895 -3.438333
C 3.903237 -1.243492 -0.909997
C 5.257621 -1.834490 -1.238668
C -0.544863 -0.593824 -3.481787
C -1.793308 -1.214253 -4.113780
C -0.396605 -1.040633 -2.026326
C -0.608710 0.934685 -3.612405
C 0.676941 1.669620 -3.210059
N 1.765531 1.443305 -4.170725
C 0.391401 3.177041 -3.139350
O 0.972586 3.882532 -4.111512
O -0.313555 3.687203 -2.299140
H 9.075885 0.245210 -2.762869
H 7.928131 -1.381439 -4.239614
H 5.685376 -0.870974 -5.134470
H 5.730507 2.900760 -3.108791
H 7.966503 2.391329 -2.204806
H 4.102900 2.343296 -5.116652
H 3.903084 0.632706 -5.486986
H 3.267035 2.018823 -2.850104
H 3.841234 -0.930375 0.129381
H 3.092667 -1.936549 -1.127997
H 5.437325 -2.699059 -0.596707
H 5.305161 -2.159540 -2.276996
H 6.050454 -1.105679 -1.067537
H 0.326365 -0.958681 -4.034113
H -1.884546 -0.936815 -5.166231
H -1.759305 -2.303860 -4.052948
H -2.696974 -0.878044 -3.597421
H -1.245950 -0.697125 -1.428889
H 0.513850 -0.657981 -1.563014
H -0.358464 -2.129764 -1.962860
H -0.846809 1.205969 -4.645764
H -1.422660 1.313846 -2.990769
H 0.962871 1.368004 -2.199639
H 1.546352 0.685406 -4.806893
H 1.494692 3.204320 -4.619229

1b-C20, ΔG = 0.6752 kcal/mol, population = 19.30 %

C 8.178882 0.921809 -3.030815
C 7.490051 2.113431 -2.817945
C 6.215298 2.286181 -3.345082
C 5.609248 1.273746 -4.091412
C 6.310150 0.086333 -4.304357
C 7.585997 -0.091089 -3.777634
C 4.201737 1.438461 -4.603580
C 3.132580 1.175905 -3.521918
C 3.361118 -0.213646 -2.928775
O 3.988107 -0.155297 -1.753650
O 3.038292 -1.236524 -3.491972
C 4.391676 -1.412051 -1.133606
C 3.271630 -1.996005 -0.299162
C -0.430694 -0.907457 -3.795737
C -1.593729 -1.477903 -4.610977
C -0.382960 -1.539706 -2.404034
C -0.552009 0.621809 -3.723687
C 0.683743 1.337770 -3.160067
N 1.803716 1.311524 -4.109169
C 0.322003 2.801410 -2.871266
O -0.404796 3.148416 -1.969098
O 0.859988 3.669885 -3.731045
H 9.171807 0.786672 -2.620810
H 7.947457 2.908949 -2.242907
H 5.684141 3.215624 -3.174631
H 5.851211 -0.707072 -4.882190
H 8.116243 -1.018982 -3.952393
H 4.041425 2.451612 -4.974627
H 4.022500 0.750128 -5.432170
H 3.242179 1.912063 -2.725686
H 4.711626 -2.095179 -1.918345
H 5.248378 -1.137601 -0.522835
H 3.633761 -2.887452 0.216552
H 2.934433 -1.280030 0.451564
H 2.424561 -2.281414 -0.921960
H 0.499618 -1.163824 -4.311650
H -1.517225 -2.564251 -4.689948
H -2.551099 -1.243604 -4.136634
H -1.609712 -1.065464 -5.622245
H -1.300526 -1.321168 -1.850365
H 0.459011 -1.176141 -1.814328
H -0.283595 -2.624377 -2.477654
H -0.750886 1.024268 -4.722027
H -1.410222 0.882490 -3.100650

H 0.951623 0.892340 -2.198534
H 1.666468 0.603156 -4.820514
H 1.414537 3.102928 -4.330625

1b-C17, ΔG = 1.7614 kcal/mol, population = 3.08 %
C 8.403202 0.395490 -3.214747
C 7.756263 1.575782 -2.855055
C 6.489083 1.855672 -3.351859
C 5.846027 0.961696 -4.211639
C 6.503894 -0.214813 -4.567693
C 7.774802 -0.497239 -4.075499
C 4.452185 1.246146 -4.710249
C 3.373512 1.104981 -3.619976
C 3.460351 -0.283639 -2.983886
O 4.051275 -0.245910 -1.789383
O 3.037738 -1.286147 -3.517319
C 4.272707 -1.508967 -1.093808
C 5.557499 -2.165301 -1.551913
C -1.586316 0.765152 -3.041555
C -1.388874 -0.115202 -1.805515
C -2.757906 0.257546 -3.886575
C -0.326449 0.830112 -3.912794
C 0.946236 1.380957 -3.246801
N 2.057813 1.341967 -4.206029
C 0.741675 2.834882 -2.807785
O 1.258654 3.730840 -3.656350
O 0.148049 3.166049 -1.808103
H 9.390585 0.176668 -2.828165
H 8.241140 2.278999 -2.189405
H 5.993397 2.777230 -3.069265
H 6.015727 -0.918512 -5.231306
H 8.269822 -1.417129 -4.360581
H 4.204926 0.562925 -5.525432
H 4.381125 2.262564 -5.101346
H 3.549137 1.846695 -2.840645
H 4.318498 -1.224108 -0.045414
H 3.407929 -2.149607 -1.256928
H 5.740829 -3.055602 -0.947123
H 5.496991 -2.465402 -2.597259
H 6.402751 -1.486716 -1.435044
H -1.826113 1.776717 -2.704318
H -2.321159 -0.200426 -1.243585
H -1.078341 -1.123643 -2.094727
H -0.634858 0.291335 -1.130384

H -2.923368 0.893370 -4.759246
H -3.679328 0.240826 -3.301266
H -2.568765 -0.759475 -4.242274
H -0.086022 -0.182140 -4.254211
H -0.532053 1.426382 -4.806612
H 1.159134 0.803247 -2.344186
H 1.878381 0.655280 -4.930205
H 1.736384 3.184455 -4.332126

1b-C28, ΔG = 1.8587 kcal/mol, population = 2.61 %
C 5.367759 3.670700 -2.042716
C 5.153381 2.536757 -1.262059
C 4.954465 1.302103 -1.867462
C 4.961229 1.177735 -3.260119
C 5.194121 2.315658 -4.030359
C 5.393610 3.555130 -3.427974
C 4.667601 -0.154907 -3.901282
C 3.184744 -0.557410 -3.751818
C 2.939065 -1.983322 -4.232970
O 3.777470 -2.846161 -3.666785
O 2.065894 -2.291496 -5.015650
C 3.626271 -4.252623 -4.019807
C 4.672784 -5.025329 -3.254691
C 0.072927 0.255575 -2.251033
C -1.427869 0.047055 -2.033344
C 0.768046 0.584885 -0.927982
C 0.296687 1.344266 -3.308106
C 1.751767 1.503349 -3.765201
N 2.279567 0.332657 -4.478848
C 1.888547 2.738832 -4.668478
O 1.525625 3.848609 -4.349475
O 2.444555 2.483998 -5.853327
H 5.517593 4.634480 -1.572926
H 5.141375 2.615313 -0.182134
H 4.787204 0.425096 -1.252556
H 5.208280 2.235587 -5.109907
H 5.564165 4.429874 -4.042889
H 4.913447 -0.128528 -4.964078
H 5.275776 -0.930983 -3.436898
H 2.950287 -0.571110 -2.681313
H 2.614091 -4.562102 -3.759674
H 3.751690 -4.347440 -5.098218
H 4.587015 -6.085438 -3.498589
H 5.676832 -4.692492 -3.520478

H 4.536216 -4.908985 -2.178882
H 0.481847 -0.686340 -2.632367
H -1.610276 -0.744331 -1.303562
H -1.894259 0.962674 -1.658790
H -1.927973 -0.228617 -2.964294
H 1.847857 0.699816 -1.037065
H 0.596318 -0.206294 -0.195591
H 0.378104 1.518422 -0.512765
H -0.315471 1.124893 -4.189173
H -0.047459 2.304760 -2.918113
H 2.385395 1.709931 -2.903218
H 1.526914 -0.223921 -4.872003
H 2.665607 1.513268 -5.804242

1b-C2, ΔG = 1.9967 kcal/mol, population = 2.07 %
C 5.706171 0.975310 -6.150660
C 6.161048 -0.016355 -5.286225
C 5.796511 0.006323 -3.944453
C 4.970499 1.015264 -3.444153
C 4.531261 2.012443 -4.318245
C 4.892502 1.992694 -5.661547
C 4.524945 1.003008 -2.003851
C 3.201964 0.231405 -1.783122
C 3.387344 -1.258603 -2.082539
O 4.137384 -1.849366 -1.148004
O 2.919662 -1.821267 -3.046366
C 4.420348 -3.272306 -1.302146
C 5.595633 -3.508839 -2.226699
C -0.549706 1.153791 -4.385892
C -1.553655 0.605837 -5.405141
C 0.587675 1.883711 -5.108179
C -0.080099 0.001825 -3.482720
C 0.773521 0.351782 -2.250883
N 2.130420 0.812229 -2.577908
C 0.072653 1.432342 -1.415811
O 0.726005 2.596189 -1.383221
O -0.984711 1.266068 -0.852005
H 5.989385 0.958939 -7.195499
H 6.802518 -0.806630 -5.655880
H 6.161185 -0.764959 -3.277818
H 3.902116 2.809834 -3.942513
H 4.540513 2.773351 -6.324296
H 5.290565 0.552381 -1.372759
H 4.361584 2.021697 -1.651320

H 2.943596 0.322030 -0.726760
H 4.633802 -3.605867 -0.289548
H 3.518712 -3.764867 -1.662081
H 6.480216 -2.976267 -1.874574
H 5.826400 -4.575648 -2.246526
H 5.369197 -3.190415 -3.243775
H -1.078755 1.882835 -3.762099
H -2.397963 0.121175 -4.910490
H -1.943967 1.407284 -6.035446
H -1.077089 -0.131201 -6.057927
H 1.199117 1.180826 -5.682049
H 1.241755 2.420605 -4.422078
H 0.181823 2.615403 -5.809490
H -0.964584 -0.516215 -3.108339
H 0.485879 -0.725265 -4.071658
H 0.809545 -0.542373 -1.624161
H 2.327662 0.675628 -3.560817
H 1.555929 2.415450 -1.902937

1b-C12, ΔG = 2.0243 kcal/mol, population = 1.97 %
C 5.407522 4.040193 -3.801763
C 6.059015 3.154064 -2.948230
C 5.873864 1.783293 -3.091656
C 5.038542 1.275960 -4.089013
C 4.394633 2.172994 -4.943214
C 4.575790 3.545674 -4.801588
C 4.788789 -0.205474 -4.190514
C 3.704223 -0.660988 -3.187124
C 3.571077 -2.183216 -3.226773
O 4.582824 -2.773567 -2.586641
O 2.676135 -2.768544 -3.794716
C 4.618782 -4.231670 -2.566834
C 3.776343 -4.780333 -1.434600
C -0.602593 1.096110 -3.581386
C -2.105469 0.872426 -3.777728
C 0.055086 1.460577 -4.916132
C 0.002191 -0.144321 -2.903772
C 1.451290 -0.051482 -2.392426
N 2.444580 0.007100 -3.466436
C 1.603861 1.191074 -1.505219
O 1.079391 1.307907 -0.421568
O 2.346733 2.163094 -2.041726
H 5.548414 5.107679 -3.689075
H 6.710710 3.530548 -2.169705

H 6.381217 1.099234 -2.420988
H 3.744673 1.793948 -5.722404
H 4.065833 4.227099 -5.470977
H 4.463337 -0.468448 -5.199454
H 5.703462 -0.761659 -3.984207
H 4.032750 -0.382840 -2.184150
H 4.284484 -4.598950 -3.535489
H 5.672219 -4.466813 -2.435028
H 3.878071 -5.866853 -1.403400
H 4.106924 -4.378165 -0.476031
H 2.722732 -4.539387 -1.575315
H -0.482858 1.946245 -2.900644
H -2.574518 1.752531 -4.221967
H -2.285303 0.024728 -4.445237
H -2.603836 0.664643 -2.828519
H 1.100701 1.743058 -4.801326
H -0.466318 2.301898 -5.377136
H 0.006081 0.618858 -5.613684
H -0.613425 -0.391284 -2.037307
H -0.053679 -1.002236 -3.580545
H 1.616540 -0.915774 -1.742110
H 2.058042 -0.334663 -4.337445
H 2.674120 1.797881 -2.903176

1b-C6, ΔG = 2.1561 kcal/mol, population = 1.58 %
C 8.146240 0.488818 -3.280851
C 7.540358 1.676970 -2.878675
C 6.252753 1.981998 -3.303787
C 5.549439 1.106615 -4.135079
C 6.166832 -0.078672 -4.533883
C 7.456812 -0.386791 -4.112272
C 4.133266 1.415988 -4.550182
C 3.120691 1.225099 -3.405854
C 3.258813 -0.190385 -2.845370
O 3.789990 -0.194603 -1.621928
O 2.919625 -1.180108 -3.456731
C 4.040954 -1.486689 -0.992261
C 5.374228 -2.054547 -1.429890
C -0.543159 -0.525312 -3.722852
C -1.446024 0.240184 -4.693280
C -1.254951 -1.772372 -3.191777
C -0.063767 0.326599 -2.539892
C 0.713887 1.606603 -2.885686
N 1.771335 1.482861 -3.900707

C -0.201403 2.781194 -3.276680
O 0.279899 3.528354 -4.272774
O -1.234766 3.049414 -2.709323
H 9.149122 0.250357 -2.949740
H 8.072846 2.366650 -2.235580
H 5.788787 2.909382 -2.987858
H 5.630230 -0.768437 -5.174231
H 7.919848 -1.312627 -4.429982
H 4.043601 2.449236 -4.889251
H 3.840734 0.768473 -5.378832
H 3.334652 1.936583 -2.609880
H 4.027045 -1.265224 0.072196
H 3.215815 -2.154287 -1.233677
H 6.181573 -1.350496 -1.226130
H 5.570692 -2.975643 -0.877755
H 5.375205 -2.283159 -2.494649
H 0.338149 -0.872899 -4.272095
H -0.937157 1.085192 -5.161684
H -1.781982 -0.417091 -5.497699
H -2.329759 0.626908 -4.180494
H -0.606943 -2.341208 -2.521399
H -1.553349 -2.428960 -4.011492
H -2.156009 -1.496906 -2.636589
H -0.921635 0.623172 -1.932145
H 0.569259 -0.293375 -1.901903
H 1.190466 1.956588 -1.965472
H 1.527213 0.779864 -4.591240
H 1.122211 3.065912 -4.533352

1b-C15, ΔG = 2.1969 kcal/mol, population = 1.48 %
C 7.081803 2.141682 -4.813467
C 6.144588 3.074468 -4.381797
C 5.199743 2.722019 -3.422456
C 5.170781 1.435600 -2.881562
C 6.128846 0.512779 -3.311436
C 7.074314 0.859816 -4.269993
C 4.117714 1.043082 -1.878661
C 2.920326 0.273694 -2.494921
C 3.365689 -1.049032 -3.107383
O 3.896426 -1.855345 -2.190021
O 3.250146 -1.322751 -4.281994
C 4.357808 -3.169357 -2.627474
C 3.213429 -4.159151 -2.677298
C -0.373268 -0.712910 -3.736238

C -1.374870 -1.313262 -4.726269
C -0.774171 -1.044628 -2.297434
C -0.256523 0.797982 -3.976515
C 0.839036 1.498392 -3.162532
N 2.197641 1.054570 -3.496969
C 0.744392 3.020361 -3.358897
O -0.254753 3.667350 -3.139378
O 1.875101 3.574937 -3.796165
H 7.818427 2.413411 -5.558948
H 6.149993 4.078436 -4.787648
H 4.490464 3.463611 -3.075755
H 6.143827 -0.482303 -2.884220
H 7.809174 0.130944 -4.588525
H 4.550140 0.417931 -1.096786
H 3.709144 1.932220 -1.397174
H 2.255697 0.016727 -1.666713
H 4.842470 -3.059537 -3.595898
H 5.098689 -3.446049 -1.881518
H 2.476924 -3.872552 -3.427623
H 3.602765 -5.145022 -2.938312
H 2.721574 -4.231449 -1.706454
H 0.600211 -1.169889 -3.936324
H -1.079394 -1.112505 -5.758400
H -1.447477 -2.395286 -4.599538
H -2.371589 -0.889830 -4.573047
H -0.875789 -2.123798 -2.167166
H -1.734156 -0.583668 -2.048190
H -0.040044 -0.698652 -1.567912
H -0.060472 0.980168 -5.038371
H -1.210459 1.278119 -3.747434
H 0.660456 1.336265 -2.097579
H 2.214099 0.553367 -4.379186
H 2.502787 2.801283 -3.861697

1b-C4, ΔG = 2.3425 kcal/mol, population = 1.15 %
C 5.282331 3.771759 -2.038686
C 5.347375 3.668875 -3.423558
C 5.210538 2.427591 -4.039656
C 5.002123 1.275028 -3.284330
C 4.955834 1.387589 -1.891275
C 5.091079 2.623990 -1.272288
C 4.777839 -0.061936 -3.944095
C 3.317313 -0.539894 -3.806145
C 3.136801 -1.968074 -4.308740

O 4.031483 -2.793949 -3.771342
O 2.263664 -2.304271 -5.079561
C 3.930274 -4.212645 -4.097447
C 2.941858 -4.907850 -3.185580
C 0.218674 0.080528 -2.245608
C -1.260040 -0.237648 -2.010758
C 0.905338 0.448601 -0.928372
C 0.350803 1.190874 -3.295967
C 1.784196 1.445818 -3.776354
N 2.370471 0.319678 -4.516936
C 1.836945 2.700466 -4.661058
O 1.396995 3.777828 -4.327924
O 2.417791 2.503017 -5.844957
H 5.383367 4.736573 -1.558110
H 5.499974 4.554398 -4.027669
H 5.254126 2.357108 -5.119163
H 4.805952 0.500359 -1.286511
H 5.047751 2.693270 -0.192553
H 5.025667 -0.007171 -5.005339
H 5.422843 -0.813321 -3.488886
H 3.080302 -0.585925 -2.736976
H 3.653135 -4.307758 -5.145500
H 4.941535 -4.585646 -3.955132
H 2.935434 -5.976105 -3.410249
H 3.223503 -4.779765 -2.139641
H 1.932781 -4.522087 -3.330802
H 0.689339 -0.826381 -2.640133
H -1.787270 0.639637 -1.625300
H -1.750114 -0.544645 -2.937162
H -1.375131 -1.043579 -1.283132
H 1.972292 0.643123 -1.049762
H 0.453580 1.347629 -0.499993
H 0.801736 -0.359180 -0.201304
H -0.261373 0.940085 -4.168658
H -0.047191 2.123592 -2.890243
H 2.420183 1.677081 -2.922365
H 1.644450 -0.265812 -4.918742
H 2.699047 1.546976 -5.811193

1b-C3, ΔG = 2.5584 kcal/mol, population = 0.80 %
C 5.823489 1.341764 -6.087806
C 4.952391 2.284266 -5.550394
C 4.554266 2.187849 -4.220830
C 5.014114 1.148044 -3.409570

C 5.896955 0.214025 -3.957317
C 6.298379 0.308020 -5.285358
C 4.526511 1.006964 -1.990080
C 3.251863 0.136778 -1.874791
C 3.548104 -1.301886 -2.303910
O 4.347343 -1.912338 -1.424395
O 3.132670 -1.806203 -3.322334
C 4.750186 -3.284206 -1.711406
C 3.703267 -4.270626 -1.238937
C -0.473395 1.052662 -4.508344
C -1.425000 0.542240 -5.595137
C 0.637646 1.899471 -5.138623
C 0.038286 -0.146498 -3.693506
C 0.833115 0.140378 -2.407964
N 2.163491 0.717833 -2.646570
C 0.038654 1.088940 -1.499421
O -1.020869 0.802542 -0.990495
O 0.612544 2.282969 -1.334736
H 6.135217 1.415180 -7.122027
H 4.583623 3.096176 -6.164690
H 3.879133 2.926613 -3.806703
H 6.275535 -0.590986 -3.338587
H 6.983731 -0.424501 -5.693218
H 4.284329 1.985246 -1.574537
H 5.299113 0.560729 -1.364619
H 2.964041 0.113976 -0.822321
H 4.936621 -3.372363 -2.780308
H 5.686333 -3.401235 -1.170691
H 4.065522 -5.287200 -1.403854
H 3.507045 -4.146095 -0.173164
H 2.769826 -4.146646 -1.787662
H -1.053662 1.697795 -3.839199
H -0.896670 -0.112619 -6.294090
H -2.253253 -0.024122 -5.164270
H -1.843040 1.373055 -6.166916
H 1.301882 1.279510 -5.748323
H 1.243519 2.415728 -4.394713
H 0.207540 2.661307 -5.791735
H -0.824022 -0.745091 -3.395317
H 0.662857 -0.785123 -4.323957
H 0.913532 -0.805089 -1.866436
H 2.395054 0.688348 -3.631062
H 1.466282 2.205765 -1.841967

1b-C7, ΔG = 2.5935 kcal/mol, population = 0.75 %
C 5.367414 3.762736 -2.181086
C 5.147893 2.688547 -1.321307
C 4.956299 1.412337 -1.835923
C 4.974707 1.186460 -3.215797
C 5.212013 2.265637 -4.065214
C 5.404947 3.546382 -3.553891
C 4.687418 -0.189000 -3.761558
C 3.203921 -0.582933 -3.592333
C 2.968759 -2.035861 -3.989907
O 3.787208 -2.858686 -3.337879
O 2.122041 -2.389655 -4.781848
C 3.693791 -4.285457 -3.628279
C 4.519768 -4.645043 -4.844921
C 0.105609 0.288131 -2.135807
C -1.391004 0.069176 -1.900477
C 0.798622 0.710579 -0.838142
C 0.308883 1.313389 -3.258531
C 1.759687 1.463459 -3.731630
N 2.302154 0.260228 -4.377284
C 1.880735 2.646635 -4.704158
O 1.495151 3.766084 -4.452487
O 2.451778 2.334704 -5.868158
H 5.511787 4.758575 -1.781715
H 5.125894 2.846394 -0.250254
H 4.784899 0.582673 -1.159511
H 5.234481 2.106930 -5.135833
H 5.579260 4.374021 -4.229871
H 4.938736 -0.237276 -4.822319
H 5.294102 -0.929199 -3.240198
H 2.964324 -0.530338 -2.524609
H 4.075502 -4.759864 -2.727581
H 2.644871 -4.545073 -3.757850
H 4.486223 -5.725639 -4.996072
H 4.131168 -4.162418 -5.741699
H 5.560844 -4.350610 -4.706515
H 0.529254 -0.668554 -2.459852
H -1.559146 -0.673151 -1.117621
H -1.872753 1.000362 -1.589123
H -1.887131 -0.278342 -2.809261
H 1.875353 0.841026 -0.959558
H 0.645955 -0.038213 -0.058535
H 0.390598 1.659371 -0.478743
H -0.304715 1.033718 -4.121299

H -0.045751 2.290982 -2.924764
H 2.394444 1.726281 -2.885678
H 1.557238 -0.321355 -4.748284
H 2.688004 1.372162 -5.763494

1b-C21, ΔG = 2.7209 kcal/mol, population = 0.61 %
C 5.730139 0.616577 -6.069309
C 6.280749 -0.057869 -4.983296
C 5.900522 0.276195 -3.688403
C 4.964035 1.285551 -3.455868
C 4.429543 1.966252 -4.552702
C 4.805607 1.633267 -5.850075
C 4.498220 1.593801 -2.055563
C 3.263686 0.761683 -1.635108
C 3.656027 -0.715681 -1.518555
O 3.128179 -1.464307 -2.488228
O 4.379652 -1.134130 -0.641992
C 3.419862 -2.892284 -2.491581
C 4.712537 -3.189724 -3.220604
C -0.517326 0.668919 -4.359226
C -1.401004 -0.197773 -5.261873
C 0.530844 1.405974 -5.199313
C 0.075021 -0.214638 -3.249465
C 0.844862 0.476629 -2.110851
N 2.140967 1.034119 -2.522139
C -0.008267 1.598181 -1.502103
O -1.055458 1.408911 -0.926660
O 0.499104 2.819557 -1.682537
H 6.024278 0.356223 -7.078197
H 7.006208 -0.845400 -5.144332
H 6.334368 -0.251836 -2.847990
H 3.713042 2.761690 -4.388096
H 4.377951 2.169267 -6.688122
H 5.295327 1.400025 -1.337995
H 4.220962 2.644800 -1.970006
H 2.991104 1.072227 -0.624801
H 3.447057 -3.240293 -1.460612
H 2.563552 -3.330804 -2.998584
H 4.848007 -4.271408 -3.279391
H 4.688022 -2.789698 -4.234440
H 5.568327 -2.765404 -2.697014
H -1.162617 1.418937 -3.888471
H -0.805236 -0.967936 -5.760322
H -2.184970 -0.697430 -4.689016

H -1.878650 0.407376 -6.034819
H 1.076584 2.153110 -4.624014
H 0.051061 1.924664 -6.031592
H 1.256782 0.705105 -5.622522
H -0.744489 -0.762342 -2.781364
H 0.743851 -0.961108 -3.686205
H 0.978206 -0.265310 -1.320004
H 2.373085 0.752571 -3.465415
H 1.359889 2.649567 -2.153677

1b-C29, ΔG = 2.7472 kcal/mol, population = 0.58 %
C 5.106826 4.031754 -2.089140
C 5.042799 2.899971 -1.279034
C 5.031291 1.633632 -1.850644
C 5.074714 1.474189 -3.239137
C 5.155901 2.613127 -4.038641
C 5.170033 3.884113 -3.470096
C 4.990125 0.098009 -3.848516
C 3.598709 -0.542168 -3.685458
C 3.661414 -2.025698 -4.045148
O 2.565007 -2.444754 -4.684182
O 4.586986 -2.741354 -3.739023
C 2.443744 -3.867330 -4.983382
C 1.863427 -4.615305 -3.802362
C 0.439498 -0.082668 -2.144570
C -1.007692 -0.506818 -1.882683
C 1.089507 0.436710 -0.859901
C 0.480838 0.956664 -3.272127
C 1.889666 1.290687 -3.776928
N 2.556682 0.159639 -4.442833
C 1.842071 2.479159 -4.748147
O 2.414600 2.236231 -5.927849
O 1.334970 3.545453 -4.482068
H 5.112094 5.019219 -1.645384
H 5.002624 3.004952 -0.202045
H 4.983248 0.758898 -1.212184
H 5.195860 2.506577 -5.115353
H 5.225132 4.757288 -4.108024
H 5.227408 0.140247 -4.912633
H 5.713598 -0.564556 -3.373286
H 3.356571 -0.549920 -2.614726
H 1.782775 -3.897670 -5.845984
H 3.424931 -4.244996 -5.263627
H 1.717888 -5.662037 -4.075657

H 2.533904 -4.576509 -2.943819
H 0.897041 -4.197647 -3.517157
H 0.985407 -0.972898 -2.474159
H -1.056694 -1.263738 -1.097247
H -1.609126 0.348583 -1.562229
H -1.466408 -0.922357 -2.782513
H 2.133683 0.720466 -1.001523
H 1.060824 -0.325341 -0.078722
H 0.556379 1.317407 -0.491159
H -0.109037 0.595598 -4.121342
H 0.008015 1.880711 -2.932239
H 2.506486 1.629417 -2.945352
H 1.866865 -0.512218 -4.757272
H 2.759740 1.305556 -5.833327

1b-C46, ΔG = 2.7692 kcal/mol, population = 0.56 %
C 5.087732 4.130553 -2.207104
C 5.078630 3.033329 -1.348521
C 5.081887 1.743786 -1.865847
C 5.087225 1.525917 -3.247141
C 5.115292 2.631299 -4.095942
C 5.113123 3.925385 -3.581879
C 5.020321 0.122962 -3.794842
C 3.659754 -0.553893 -3.545830
C 3.763420 -2.051171 -3.835184
O 2.648971 -2.546511 -4.377744
O 4.733567 -2.718026 -3.557578
C 2.604367 -3.979133 -4.641045
C 1.263213 -4.280419 -5.263955
C 0.495628 -0.050024 -1.969116
C -0.944089 -0.464592 -1.655593
C 1.168237 0.543283 -0.728982
C 0.508600 0.925712 -3.153049
C 1.905348 1.235969 -3.704158
N 2.567357 0.066116 -4.303732
C 1.825367 2.353491 -4.754376
O 2.364847 2.029103 -5.930140
O 1.319640 3.433981 -4.550113
H 5.080890 5.135981 -1.805705
H 5.069019 3.183263 -0.276146
H 5.075507 0.896475 -1.189676
H 5.124870 2.480061 -5.168043
H 5.125413 4.771496 -4.257429
H 5.212076 0.126209 -4.869027

H 5.784808 -0.495451 -3.324163
H 3.454832 -0.510971 -2.467955
H 3.433051 -4.227878 -5.303355
H 2.746698 -4.501189 -3.695081
H 1.193380 -5.349954 -5.468259
H 0.449797 -4.005709 -4.591289
H 1.139465 -3.742852 -6.204830
H 1.040411 -0.954801 -2.259768
H -0.974406 -1.174013 -0.826158
H -1.544339 0.405507 -1.374896
H -1.416872 -0.934186 -2.520871
H 0.642353 1.445455 -0.404442
H 2.210139 0.815513 -0.905564
H 1.152539 -0.170749 0.096717
H -0.097244 0.516622 -3.968502
H 0.039596 1.865867 -2.854821
H 2.532236 1.636541 -2.908464
H 1.879495 -0.640931 -4.533240
H 2.713384 1.106661 -5.780911

1b-C16, ΔG = 2.9298 kcal/mol, population = 0.43 %
C 5.124737 3.600309 -1.991892
C 5.224406 3.440460 -3.369630
C 5.151920 2.169099 -3.932375
C 4.972004 1.043065 -3.130308
C 4.891168 1.213457 -1.745068
C 4.964248 2.480324 -1.179094
C 4.794630 -0.324094 -3.737996
C 3.321335 -0.789593 -3.696943
C 3.181157 -2.212231 -4.224011
O 3.980785 -3.054697 -3.572531
O 2.418692 -2.531478 -5.110465
C 3.971707 -4.455346 -3.980745
C 4.901458 -4.684321 -5.153289
C -0.127655 1.683112 -2.018561
C 0.843165 1.909986 -0.856740
C -1.450692 1.103180 -1.511871
C 0.459738 0.750233 -3.087152
C 1.793756 1.172040 -3.718230
N 2.441109 0.085424 -4.471499
C 1.679596 2.393139 -4.639099
O 1.088919 3.410616 -4.355280
O 2.323454 2.252975 -5.801175
H 5.175096 4.588560 -1.552787

H 5.354282 4.304690 -4.008880
H 5.221334 2.052950 -5.006678
H 4.756147 0.347839 -1.106760
H 4.892821 2.595397 -0.104821
H 5.124505 -0.320095 -4.778179
H 5.395336 -1.055817 -3.198238
H 3.006693 -0.831970 -2.646349
H 4.304528 -4.987307 -3.092905
H 2.947650 -4.742203 -4.212247
H 4.923930 -5.749263 -5.392198
H 4.561954 -4.143046 -6.036350
H 5.916100 -4.366137 -4.910490
H -0.331814 2.647644 -2.486572
H 1.751700 2.426970 -1.169818
H 0.374491 2.516857 -0.079235
H 1.138964 0.958248 -0.404529
H -1.290861 0.134627 -1.028449
H -2.158621 0.956018 -2.330881
H -1.914230 1.768941 -0.780843
H 0.627349 -0.230422 -2.628568
H -0.273410 0.598999 -3.885554
H 2.480821 1.481941 -2.932293
H 1.741307 -0.492399 -4.929492
H 2.723469 1.342561 -5.743899

1b-C45, ΔG = 3.0321 kcal/mol, population = 0.36 %
C 7.132190 1.725754 -4.762676
C 7.101882 0.637958 -3.893898
C 6.132436 0.564832 -2.900169
C 5.172794 1.571443 -2.759079
C 5.224500 2.664391 -3.625623
C 6.193649 2.742789 -4.621538
C 4.086556 1.458757 -1.722828
C 2.880889 0.590051 -2.177167
C 3.344869 -0.853802 -2.339574
O 3.401367 -1.237507 -3.617566
O 3.665711 -1.547121 -1.400872
C 3.866665 -2.587878 -3.911733
C 2.723382 -3.578711 -3.856561
C -0.179983 -0.905064 -3.518399
C -0.957080 -1.789767 -4.495618
C -0.782464 -0.990387 -2.114820
C -0.132652 0.534387 -4.045468
C 0.790551 1.473740 -3.260905

N 2.216489 1.137640 -3.361541
C 0.588480 2.927505 -3.724302
O 1.719935 3.536932 -4.079143
O -0.487437 3.480495 -3.760133
H 7.887474 1.784579 -5.536178
H 7.837162 -0.151605 -3.986968
H 6.124187 -0.280768 -2.222881
H 4.515004 3.474593 -3.508198
H 6.217569 3.600201 -5.282537
H 4.480149 1.026278 -0.801699
H 3.690669 2.445485 -1.480783
H 2.177593 0.575109 -1.343926
H 4.284826 -2.507858 -4.912002
H 4.656743 -2.838704 -3.206892
H 3.094134 -4.568840 -4.127970
H 2.299635 -3.635664 -2.853892
H 1.935576 -3.304748 -4.558875
H 0.842236 -1.287408 -3.476509
H -0.974442 -2.826425 -4.153188
H -1.992330 -1.449395 -4.587863
H -0.506532 -1.769907 -5.490437
H -0.806696 -2.026209 -1.770759
H -0.212993 -0.418076 -1.380268
H -1.807892 -0.610234 -2.111285
H 0.196626 0.528832 -5.089919
H -1.138932 0.958800 -4.037444
H 0.500737 1.471454 -2.208358
H 2.399322 0.553490 -4.167885
H 2.413106 2.833280 -3.931025

1b-C31, ΔG = 3.0961 kcal/mol, population = 0.32 %
C 5.683505 3.821248 -2.581428
C 5.410253 3.579902 -3.923314
C 5.163871 2.282433 -4.363557
C 5.179436 1.210883 -3.471926
C 5.469665 1.462703 -2.127784
C 5.718383 2.756020 -1.684143
C 4.830774 -0.182062 -3.927038
C 3.400044 -0.583529 -3.503201
C 3.092481 -2.021308 -3.902365
O 3.992354 -2.870503 -3.415224
O 2.134180 -2.347805 -4.569959
C 3.803837 -4.285470 -3.712572
C 4.933908 -5.040010 -3.055796

C -0.406809 0.187768 -2.825771
C -1.123492 0.916830 -3.965245
C -1.392148 -0.157399 -1.705788
C 0.770068 0.980771 -2.240825
C 1.890312 1.370986 -3.217183
N 2.384240 0.294527 -4.084895
C 1.547771 2.589389 -4.085418
O 1.956190 2.483455 -5.353121
O 1.001087 3.584123 -3.668298
H 5.872706 4.829872 -2.236504
H 5.386416 4.400724 -4.629108
H 4.950495 2.101917 -5.409715
H 5.493707 0.639707 -1.422620
H 5.939408 2.933902 -0.639085
H 4.897513 -0.249957 -5.014097
H 5.527486 -0.904126 -3.501277
H 3.351832 -0.564982 -2.408311
H 2.829815 -4.587082 -3.327597
H 3.802031 -4.408006 -4.795498
H 4.925311 -4.894196 -1.974953
H 4.820302 -6.106132 -3.258746
H 5.899469 -4.717541 -3.447231
H -0.021463 -0.759879 -3.218511
H -0.475383 1.089810 -4.826601
H -1.972889 0.327744 -4.316520
H -1.498376 1.887132 -3.631412
H -0.900183 -0.705431 -0.899186
H -2.210505 -0.773709 -2.083494
H -1.825213 0.751877 -1.279666
H 0.400224 1.898684 -1.778086
H 1.220093 0.388128 -1.440275
H 2.735683 1.721334 -2.621052
H 1.618849 -0.286170 -4.413976
H 2.386899 1.588891 -5.394341

1b-C18, ΔG = 3.2392 kcal/mol, population = 0.25 %
C 5.072959 3.640188 -1.903035
C 5.155386 3.583666 -3.290054
C 5.137138 2.353247 -3.941419
C 5.030654 1.165577 -3.219336
C 4.965429 1.233162 -1.824472
C 4.984480 2.458812 -1.170145
C 4.911934 -0.163294 -3.919686
C 3.463530 -0.700471 -3.895430

C 3.375670 -2.091956 -4.510293
O 4.245787 -2.929987 -3.950489
O 2.595168 -2.393374 -5.387358
C 4.241140 -4.317707 -4.401711
C 3.186274 -5.123074 -3.673396
C -0.066988 1.451887 -1.996026
C 0.904750 1.574375 -0.819351
C -1.378332 0.801436 -1.548310
C 0.532627 0.642800 -3.155146
C 1.836887 1.175211 -3.764304
N 2.532170 0.181732 -4.598831
C 1.646362 2.450494 -4.595070
O 2.295429 2.433850 -5.762591
O 0.993115 3.405957 -4.241396
H 5.081220 4.596122 -1.394959
H 5.229223 4.496615 -3.867476
H 5.192553 2.316755 -5.022177
H 4.885636 0.318598 -1.248081
H 4.926301 2.493697 -0.089459
H 5.226820 -0.071199 -4.960378
H 5.554408 -0.900205 -3.438190
H 3.165983 -0.826365 -2.846366
H 4.089867 -4.330697 -5.479424
H 5.244549 -4.668555 -4.173337
H 3.257929 -6.168641 -3.979026
H 3.336221 -5.071101 -2.594244
H 2.184044 -4.766060 -3.910020
H -0.288501 2.455439 -2.363487
H 1.805001 2.133455 -1.079556
H 0.429877 2.092465 0.016353
H 1.215222 0.585910 -0.466759
H -1.202014 -0.211051 -1.172608
H -2.089177 0.732703 -2.375064
H -1.846766 1.377964 -0.747863
H 0.744588 -0.368217 -2.790696
H -0.209810 0.537405 -3.951887
H 2.515824 1.464078 -2.963190
H 1.858103 -0.397095 -5.092836
H 2.745811 1.545489 -5.772378

1b-C53, ΔG = 3.3170 kcal/mol, population = 0.22 %
C 7.190180 2.325770 -4.020751
C 6.086111 3.064564 -3.606815
C 5.029444 2.434987 -2.956414

C 5.052443 1.060605 -2.712697
C 6.174140 0.332317 -3.118807
C 7.232973 0.957461 -3.768997
C 3.885617 0.377591 -2.047818
C 2.879403 -0.250586 -3.040844
C 3.519051 -1.321421 -3.913401
O 4.118621 -2.257040 -3.179735
O 3.465659 -1.332636 -5.123546
C 4.771502 -3.345651 -3.895752
C 5.386633 -4.260199 -2.864592
C -1.452502 0.851140 -2.772573
C -1.194671 0.465092 -1.313901
C -2.675781 0.108617 -3.315734
C -0.236344 0.556669 -3.663171
C 1.038067 1.363358 -3.371123
N 2.258854 0.747976 -3.906789
C 0.962707 2.807691 -3.887245
O -0.008560 3.521092 -3.784319
O 2.095229 3.236026 -4.452102
H 8.014222 2.813238 -4.526301
H 6.047599 4.131770 -3.786566
H 4.181973 3.021664 -2.624080
H 6.223506 -0.729851 -2.916080
H 8.094222 0.376116 -4.074221
H 4.232308 -0.411541 -1.381011
H 3.335653 1.094360 -1.436046
H 2.123785 -0.776759 -2.443053
H 4.021688 -3.853718 -4.501841
H 5.519622 -2.914301 -4.561040
H 5.884293 -5.089264 -3.370179
H 6.127192 -3.730899 -2.263808
H 4.624180 -4.670843 -2.201650
H -1.653496 1.923146 -2.818851
H -0.371317 1.030225 -0.872771
H -2.080333 0.653340 -0.703672
H -0.951858 -0.598757 -1.233555
H -3.559810 0.315405 -2.708871
H -2.509412 -0.972828 -3.306249
H -2.894779 0.405283 -4.343974
H 0.019343 -0.502056 -3.551602
H -0.505539 0.703091 -4.713173
H 1.169428 1.461278 -2.290769
H 2.080828 0.327237 -4.814491
H 2.705118 2.450776 -4.411367

1b-C10, ΔG = 3.4488 kcal/mol, population = 0.18 %
C 8.244265 1.032163 -3.058269
C 7.649130 0.064740 -3.861669
C 6.354747 0.252150 -4.337579
C 5.636696 1.404883 -4.016508
C 6.244666 2.370939 -3.211826
C 7.538288 2.188554 -2.736332
C 4.211083 1.581290 -4.473045
C 3.194184 1.182384 -3.386625
C 3.443939 -0.270909 -2.985328
O 3.974517 -0.374399 -1.766552
O 3.195419 -1.207969 -3.712626
C 4.306031 -1.711266 -1.284682
C 3.090753 -2.401088 -0.701304
C -0.325003 -0.810380 -3.920255
C -1.293795 -0.038624 -4.819420
C -0.926614 -2.156912 -3.509093
C 0.083248 -0.033504 -2.661611
C 0.760379 1.328309 -2.881984
N 1.835608 1.378159 -3.884894
C -0.240262 2.458467 -3.183383
O -1.294996 2.596484 -2.608739
O 0.188457 3.323168 -4.105562
H 9.251814 0.888959 -2.688667
H 8.192431 -0.835488 -4.120764
H 5.895509 -0.505351 -4.961403
H 5.700337 3.273130 -2.957132
H 7.996659 2.949167 -2.116547
H 4.012640 2.622016 -4.731900
H 4.021629 0.974755 -5.360793
H 3.334276 1.816702 -2.512668
H 4.736977 -2.277346 -2.108596
H 5.068685 -1.533799 -0.530485
H 3.392901 -3.355546 -0.266190
H 2.641497 -1.793569 0.085451
H 2.342561 -2.597966 -1.468520
H 0.580545 -1.029482 -4.495820
H -0.860769 0.885324 -5.208013
H -1.577259 -0.646430 -5.680915
H -2.204187 0.223609 -4.275471
H -1.172484 -2.757668 -4.386989
H -1.845208 -2.010669 -2.933931
H -0.230749 -2.729398 -2.892016

H -0.797055 0.139747 -2.038561
H 0.760544 -0.656396 -2.074609
H 1.197984 1.630917 -1.926268
H 1.658181 0.720231 -4.637487
H 1.066759 2.951049 -4.391558

1b-C30, ΔG = 3.5348 kcal/mol, population = 0.15 %
C 5.149797 4.074323 -2.249835
C 5.048216 3.019915 -1.344731
C 5.004451 1.709399 -1.803998
C 5.054111 1.427805 -3.172720
C 5.173314 2.489767 -4.067862
C 5.218342 3.804703 -3.612018
C 4.933850 0.006250 -3.659479
C 3.525618 -0.580371 -3.449094
C 3.553030 -2.090497 -3.684070
O 2.438546 -2.537665 -4.270840
O 4.466723 -2.798900 -3.329656
C 2.324446 -3.968994 -4.529785
C 2.983176 -4.334256 -5.842534
C 0.374322 0.127882 -1.977403
C -1.087346 -0.218274 -1.682714
C 1.038011 0.750997 -0.747015
C 0.452548 1.048545 -3.202391
C 1.871795 1.287756 -3.729409
N 2.507255 0.077257 -4.275247
C 1.864154 2.374111 -4.815487
O 1.378322 3.472490 -4.665283
O 2.447024 2.001796 -5.955472
H 5.178856 5.096323 -1.893943
H 5.003624 3.219587 -0.281394
H 4.924936 0.895456 -1.092409
H 5.217518 2.289287 -5.130955
H 5.301688 4.616968 -4.323135
H 5.176320 -0.052005 -4.721724
H 5.637909 -0.630639 -3.123731
H 3.277391 -0.486924 -2.383995
H 2.762930 -4.505652 -3.690831
H 1.251816 -4.144616 -4.552841
H 2.824840 -5.396008 -6.040656
H 2.549862 -3.763981 -6.665147
H 4.056687 -4.148856 -5.809048
H 0.891175 -0.808404 -2.213538
H -1.164065 -0.892526 -0.827318

H -1.658493 0.684869 -1.449866
H -1.559753 -0.703118 -2.539769
H 2.094842 0.971858 -0.905824
H 0.970578 0.077444 0.109498
H 0.541641 1.687797 -0.479180
H -0.146661 0.623807 -4.014795
H 0.008563 2.015893 -2.957578
H 2.495567 1.690124 -2.932204
H 1.799606 -0.600321 -4.532184
H 2.763307 1.075350 -5.764151

1b-C9, ΔG = 3.6797 kcal/mol, population = 0.12 %
C 5.657586 3.888689 -2.697388
C 5.404711 3.561494 -4.025046
C 5.177176 2.236527 -4.387010
C 5.192329 1.222833 -3.430184
C 5.462102 1.560818 -2.100719
C 5.691627 2.881668 -1.735105
C 4.860751 -0.199280 -3.799237
C 3.433425 -0.588886 -3.352753
C 3.146857 -2.053932 -3.657866
O 4.054214 -2.854643 -3.103539
O 2.198199 -2.433545 -4.310496
C 3.924960 -4.290440 -3.329617
C 4.561249 -4.692701 -4.643071
C -0.351823 0.184396 -2.673798
C -1.103815 0.785773 -3.863464
C -1.309452 -0.074918 -1.507637
C 0.814088 1.057328 -2.189255
C 1.910861 1.374044 -3.217446
N 2.409318 0.234018 -3.996939
C 1.536347 2.504934 -4.185019
O 1.950455 2.301434 -5.439077
O 0.965021 3.518030 -3.854087
H 5.831243 4.918848 -2.413368
H 5.381239 4.336813 -4.780524
H 4.977467 1.989347 -5.422239
H 5.483261 0.783323 -1.345399
H 5.896105 3.126588 -0.700305
H 4.930652 -0.334422 -4.879710
H 5.564600 -0.885263 -3.328148
H 3.380654 -0.496536 -2.261794
H 4.438692 -4.734513 -2.480595
H 2.869421 -4.553149 -3.292120

H 5.609905 -4.394134 -4.672620
H 4.510789 -5.777998 -4.749475
H 4.040334 -4.242185 -5.487881
H 0.051349 -0.786796 -2.981540
H -0.471691 0.897534 -4.746519
H -1.940929 0.145023 -4.147676
H -1.501169 1.772142 -3.612996
H -0.789382 -0.529838 -0.661679
H -2.117620 -0.744712 -1.808259
H -1.758791 0.860536 -1.162698
H 0.429199 2.008315 -1.813731
H 1.288848 0.557945 -1.340706
H 2.760364 1.788963 -2.670777
H 1.647397 -0.375996 -4.277021
H 2.396336 1.413630 -5.404169

1b-C14, ΔG = 3.6860 kcal/mol, population = 0.12 %
C 6.906929 2.369318 -4.589516
C 5.898315 3.150262 -4.033967
C 5.018891 2.597889 -3.107839
C 5.126491 1.260282 -2.723503
C 6.154669 0.491057 -3.275373
C 7.035421 1.038402 -4.202319
C 4.137312 0.656295 -1.760850
C 2.990079 -0.116765 -2.453746
C 3.493445 -1.308182 -3.256723
O 4.227449 -2.126483 -2.504305
O 3.236237 -1.492156 -4.426253
C 4.753955 -3.335834 -3.128371
C 3.731056 -4.451490 -3.103323
C -0.632254 -0.205565 -3.957668
C -0.846765 0.734082 -5.146667
C -1.885292 -1.048498 -3.705323
C -0.243289 0.527116 -2.666277
C 1.034464 1.379157 -2.722128
N 2.201842 0.740999 -3.334317
C 0.824363 2.754401 -3.374228
O 1.849021 3.152266 -4.133920
O -0.145709 3.451356 -3.188124
H 7.592427 2.795957 -5.310845
H 5.795842 4.189901 -4.319252
H 4.244702 3.216943 -2.671621
H 6.272307 -0.541286 -2.971509
H 7.825507 0.425957 -4.618897

H 4.635699 -0.026941 -1.073382
H 3.679538 1.442480 -1.158721
H 2.366323 -0.547838 -1.660940
H 5.065687 -3.093420 -4.142628
H 5.628579 -3.577793 -2.529458
H 2.860300 -4.202128 -3.709452
H 4.180861 -5.360134 -3.508010
H 3.406398 -4.655990 -2.082299
H 0.175239 -0.900679 -4.214365
H -1.624292 1.468915 -4.925489
H 0.057906 1.280961 -5.420008
H -1.156108 0.167676 -6.027248
H -2.149182 -1.628351 -4.591974
H -2.737006 -0.409556 -3.455989
H -1.733846 -1.744978 -2.877735
H -1.063000 1.176889 -2.351244
H -0.113540 -0.215864 -1.875499
H 1.303601 1.632426 -1.691976
H 1.943397 0.208199 -4.158179
H 2.495468 2.397714 -4.094076

1b-C49, ΔG = 3.7475 kcal/mol, population = 0.11 %
C 5.803839 3.543731 -2.173804
C 5.690972 3.494134 -3.558173
C 5.299387 2.314546 -4.185999
C 5.014039 1.170313 -3.443304
C 5.144520 1.227617 -2.052195
C 5.532077 2.403051 -1.421283
C 4.578614 -0.108483 -4.117187
C 3.159863 -0.564832 -3.715060
C 2.887550 -1.988125 -4.190853
O 3.808713 -2.837598 -3.744236
O 1.934693 -2.306358 -4.868616
C 3.652601 -4.241580 -4.104042
C 4.785764 -5.001433 -3.458788
C -0.113447 0.692382 -1.790903
C 0.063258 -0.817784 -1.968746
C -1.559186 1.008795 -1.395316
C 0.257356 1.519488 -3.030382
C 1.733022 1.487966 -3.462205
N 2.103162 0.297012 -4.238568
C 2.021742 2.738515 -4.314316
O 1.920321 3.871476 -3.901063
O 2.365110 2.463113 -5.572061

H 6.103274 4.461116 -1.682996
H 5.901420 4.374289 -4.152707
H 5.203239 2.287462 -5.263930
H 4.942113 0.345360 -1.455957
H 5.625103 2.429440 -0.342718
H 4.610344 0.006975 -5.201732
H 5.270697 -0.908234 -3.852025
H 3.122251 -0.609599 -2.619713
H 2.677876 -4.577613 -3.750757
H 3.672771 -4.317778 -5.191079
H 4.699599 -6.059433 -3.711507
H 5.751479 -4.641207 -3.815412
H 4.752830 -4.904674 -2.372906
H 0.540056 1.018176 -0.973686
H 1.107763 -1.107018 -2.072185
H -0.330244 -1.348901 -1.099687
H -0.477619 -1.175725 -2.849183
H -1.699792 2.077937 -1.223352
H -1.838132 0.478353 -0.482685
H -2.250707 0.701151 -2.185063
H -0.362893 1.212191 -3.880625
H 0.005708 2.560911 -2.824963
H 2.366225 1.573982 -2.576997
H 1.295190 -0.268977 -4.468467
H 2.405966 1.465884 -5.585735

1b-C43, ΔG = 3.8667 kcal/mol, population = 0.09 %
C 7.225548 1.796712 -4.330543
C 7.148355 0.666642 -3.521344
C 6.047720 0.472201 -2.694448
C 4.999598 1.397019 -2.665736
C 5.099564 2.536523 -3.465771
C 6.199744 2.735711 -4.294566
C 3.784916 1.157579 -1.806225
C 2.734906 0.210022 -2.437906
C 3.337783 -1.173017 -2.653788
O 3.288812 -1.572292 -3.925770
O 3.796344 -1.829528 -1.746302
C 3.834985 -2.883569 -4.260062
C 5.326851 -2.805309 -4.502507
C -0.688246 -0.462880 -3.093472
C -1.806147 -1.251928 -3.779671
C -1.075896 -0.122239 -1.652992
C -0.360957 0.790409 -3.915572

C 0.904572 1.540656 -3.478911
N 2.137331 0.763128 -3.656490
C 1.019126 2.859123 -4.259725
O 0.145487 3.695580 -4.298803
O 2.178309 3.008257 -4.901298
H 8.082369 1.948961 -4.974710
H 7.947970 -0.063643 -3.530791
H 6.003122 -0.404243 -2.060971
H 4.315996 3.282594 -3.433352
H 6.255935 3.626395 -4.907995
H 4.077204 0.725606 -0.848473
H 3.281999 2.102146 -1.593840
H 1.966494 0.047327 -1.675409
H 3.588530 -3.573560 -3.455431
H 3.294578 -3.167203 -5.159755
H 5.682819 -3.775874 -4.853154
H 5.556707 -2.058716 -5.263399
H 5.863800 -2.552360 -3.589460
H 0.198819 -1.105219 -3.070365
H -2.719004 -0.652907 -3.842402
H -1.521942 -1.539109 -4.794306
H -2.040150 -2.161585 -3.223020
H -0.285609 0.409765 -1.120315
H -1.295412 -1.030322 -1.088254
H -1.968680 0.508949 -1.636444
H -0.236873 0.511175 -4.967334
H -1.204041 1.483251 -3.873507
H 0.817468 1.833913 -2.430399
H 2.001482 0.026513 -4.337842
H 2.694679 2.190944 -4.660472

1b-C8, ΔG = 3.8868 kcal/mol, population = 0.08 %
C 5.590219 3.943367 -2.573850
C 5.689836 2.872409 -1.688237
C 5.513929 1.571708 -2.144963
C 5.233220 1.318144 -3.490810
C 5.152720 2.396449 -4.370644
C 5.326114 3.701134 -3.917448
C 4.954995 -0.085953 -3.959843
C 3.543301 -0.558819 -3.544771
C 3.299242 -2.001312 -3.969907
O 4.243246 -2.815765 -3.504141
O 2.353419 -2.354667 -4.641247
C 4.120719 -4.239224 -3.802378

C 3.210210 -4.927793 -2.808116
C -0.286914 0.007269 -2.844537
C -1.038434 0.715873 -3.973720
C -1.251655 -0.404498 -1.729436
C 0.848897 0.850607 -2.248432
C 1.943850 1.316142 -3.220318
N 2.489157 0.283732 -4.110704
C 1.538262 2.532863 -4.063460
O 1.956091 2.476478 -5.331313
O 0.937849 3.487755 -3.627513
H 5.722075 4.957418 -2.218321
H 5.903686 3.051694 -0.641929
H 5.586408 0.743897 -1.448815
H 4.944531 2.214931 -5.417613
H 5.251873 4.526754 -4.614024
H 5.028887 -0.140382 -5.047295
H 5.684076 -0.777659 -3.538012
H 3.494480 -0.561950 -2.449751
H 3.762462 -4.348034 -4.824300
H 5.141454 -4.607553 -3.735825
H 3.197335 -5.999478 -3.015705
H 3.568801 -4.781202 -1.788481
H 2.189404 -4.553253 -2.882557
H 0.146535 -0.913532 -3.250483
H -0.399358 0.930066 -4.832615
H -1.859179 0.092183 -4.333253
H -1.458194 1.663219 -3.627619
H -0.730468 -0.938795 -0.931966
H -2.038023 -1.056494 -2.114984
H -1.729962 0.474559 -1.288287
H 0.432852 1.739588 -1.768493
H 1.331795 0.268394 -1.459596
H 2.772514 1.696522 -2.619249
H 1.751324 -0.323828 -4.453872
H 2.433585 1.606914 -5.390253

1b-C32, ΔG = 3.9188 kcal/mol, population = 0.08 %
C 7.131524 2.299829 -4.017945
C 7.181904 0.935081 -3.748327
C 6.136085 0.317968 -3.070456
C 5.020222 1.048390 -2.655312
C 4.989756 2.419263 -2.916291
C 6.033547 3.041941 -3.593716
C 3.866683 0.369120 -1.964354

C 2.859717 -0.296373 -2.930926
C 3.469020 -1.439225 -3.733496
O 4.025704 -2.353258 -2.938368
O 3.406939 -1.524595 -4.940150
C 4.566538 -3.556954 -3.563184
C 5.991716 -3.353159 -4.030769
C -1.448152 0.921203 -2.754067
C -1.195681 0.680314 -1.263696
C -2.673908 0.134416 -3.224607
C -0.232925 0.536722 -3.610865
C 1.056752 1.335842 -3.370306
N 2.266337 0.662473 -3.859463
C 1.012276 2.744211 -3.980932
O 2.157931 3.115585 -4.559325
O 0.053221 3.479859 -3.935231
H 7.944829 2.782049 -4.545419
H 8.037968 0.350381 -4.061684
H 6.190130 -0.740145 -2.853960
H 4.145809 3.008345 -2.579525
H 5.989138 4.106409 -3.787764
H 4.226751 -0.397444 -1.278352
H 3.313537 1.095986 -1.367640
H 2.088266 -0.770602 -2.309849
H 4.506197 -4.305808 -2.777230
H 3.910820 -3.840481 -4.384187
H 6.641446 -3.075826 -3.199836
H 6.363490 -4.288849 -4.452731
H 6.050562 -2.584436 -4.800878
H -1.644480 1.984375 -2.905222
H -0.373406 1.285521 -0.877239
H -2.083535 0.929960 -0.679184
H -0.955857 -0.370542 -1.075918
H -2.884481 0.322862 -4.279783
H -3.559949 0.410294 -2.648939
H -2.515983 -0.941175 -3.100261
H 0.000043 -0.516163 -3.419848
H -0.492626 0.610046 -4.670968
H 1.188835 1.502645 -2.298566
H 2.083081 0.190620 -4.740470
H 2.754097 2.325041 -4.461419

1b-C41, ΔG = 4.0487 kcal/mol, population = 0.06 %
C 4.971870 3.911446 -2.138492
C 5.060356 3.672869 -3.505602

C 5.113380 2.366848 -3.985009
C 5.071448 1.282955 -3.109092
C 5.001212 1.534432 -1.735729
C 4.949158 2.836247 -1.252827
C 5.036033 -0.131646 -3.625757
C 3.641310 -0.778305 -3.496156
C 3.740811 -2.263763 -3.836522
O 2.726775 -2.691300 -4.594178
O 4.631192 -2.973230 -3.427313
C 2.702758 -4.098590 -4.978413
C 3.530881 -4.332183 -6.223671
C -0.013181 1.521468 -1.940885
C 0.951803 1.910509 -0.817865
C -1.286164 0.893726 -1.366907
C 0.628380 0.545662 -2.937757
C 1.915705 1.011386 -3.631823
N 2.629465 -0.089855 -4.304252
C 1.688852 2.131506 -4.655097
O 2.296183 1.921690 -5.825982
O 1.042274 3.131971 -4.440726
H 4.924143 4.926065 -1.764022
H 5.083214 4.502067 -4.201532
H 5.172424 2.188322 -5.051401
H 4.972966 0.703159 -1.040599
H 4.886990 3.012998 -0.186369
H 5.325855 -0.154676 -4.677530
H 5.740094 -0.752460 -3.071660
H 3.356540 -0.767448 -2.434975
H 3.057128 -4.690433 -4.136834
H 1.649009 -4.304451 -5.149265
H 3.441089 -5.377892 -6.523847
H 3.178212 -3.708353 -7.045952
H 4.583954 -4.117745 -6.042035
H -0.291071 2.427231 -2.482578
H 1.814162 2.468539 -1.186026
H 0.447765 2.538602 -0.080426
H 1.324661 1.020997 -0.300834
H -1.053135 -0.017872 -0.808374
H -1.990538 0.630221 -2.159410
H -1.788211 1.583708 -0.685441
H 0.879506 -0.374396 -2.398676
H -0.104058 0.268855 -3.702321
H 2.585663 1.441234 -2.889533
H 1.959365 -0.772006 -4.641506

H 2.756702 1.046625 -5.700321

1b-C13, ΔG = 4.0625 kcal/mol, population = 0.06 %
C 6.889640 2.105181 -4.637999
C 6.967945 0.792652 -4.181202
C 6.069673 0.330938 -3.225062
C 5.074446 1.166824 -2.713494
C 5.017880 2.486009 -3.166418
C 5.914727 2.953598 -4.121987
C 4.066188 0.649795 -1.721021
C 2.875359 -0.092449 -2.372710
C 3.292939 -1.367338 -3.095734
O 3.957807 -2.193662 -2.289203
O 3.009967 -1.611305 -4.248411
C 4.339828 -3.499377 -2.819973
C 5.654863 -3.437782 -3.566888
C -0.735564 -0.091964 -3.902495
C -0.893946 0.789031 -5.144112
C -2.032234 -0.852885 -3.613048
C -0.316778 0.691837 -2.650522
C 0.999109 1.480308 -2.745674
N 2.140301 0.754964 -3.308337
C 0.854527 2.822175 -3.479593
O 1.900614 3.130027 -4.252004
O -0.085183 3.570349 -3.343626
H 7.588179 2.466248 -5.382256
H 7.731554 0.128038 -4.566013
H 6.146664 -0.686493 -2.866594
H 4.270873 3.157118 -2.760915
H 5.852371 3.980094 -4.461318
H 4.537196 -0.028863 -1.010009
H 3.653523 1.480843 -1.146718
H 2.225409 -0.431422 -1.556615
H 4.410751 -4.126813 -1.934904
H 3.532640 -3.859532 -3.454984
H 5.916670 -4.441556 -3.906940
H 5.584979 -2.788996 -4.439496
H 6.456953 -3.080354 -2.920145
H 0.036700 -0.840433 -4.112654
H 0.040286 1.270388 -5.439854
H -1.228314 0.190662 -5.993891
H -1.631952 1.575567 -4.970908
H -1.921619 -1.507363 -2.745658
H -2.321690 -1.468443 -4.467058

H -2.850146 -0.156774 -3.407349
H -1.106334 1.395567 -2.377118
H -0.224464 -0.011811 -1.819571
H 1.273613 1.781165 -1.729835
H 1.862953 0.189448 -4.104007
H 2.514313 2.353225 -4.162060

1b-C50, ΔG = 4.1315 kcal/mol, population = 0.06 %
C 7.179104 2.125392 -4.024688
C 6.069225 2.931080 -3.790520
C 5.002320 2.447855 -3.039264
C 5.021105 1.154705 -2.514208
C 6.150260 0.362593 -2.739273
C 7.218155 0.840461 -3.490918
C 3.840551 0.617750 -1.746890
C 2.884150 -0.251839 -2.598849
C 3.601076 -1.513549 -3.063441
O 3.598987 -1.658674 -4.389136
O 4.101565 -2.297763 -2.288799
C 4.255277 -2.832945 -4.954307
C 5.740938 -2.598321 -5.124297
C -1.440713 0.995167 -2.761913
C -1.240993 1.059220 -1.245717
C -2.656064 0.131378 -3.108515
C -0.200722 0.443390 -3.479785
C 1.090969 1.265966 -3.360259
N 2.298116 0.502243 -3.708216
C 1.070544 2.544594 -4.211014
O 2.217480 2.780573 -4.853737
O 0.127507 3.298003 -4.291072
H 8.010370 2.498525 -4.609531
H 6.033327 3.936973 -4.190055
H 4.148956 3.087817 -2.853171
H 6.194693 -0.634304 -2.320013
H 8.082721 0.209658 -3.656366
H 4.173309 0.007920 -0.906623
H 3.259284 1.442822 -1.333102
H 2.102117 -0.621730 -1.923203
H 4.049331 -3.685951 -4.310625
H 3.757695 -2.972023 -5.910911
H 6.179820 -3.452156 -5.643960
H 5.926208 -1.702117 -5.717305
H 6.237656 -2.491192 -4.160976
H -1.622475 2.006390 -3.130259

H -0.429624 1.731785 -0.961128
H -2.147080 1.419080 -0.754027
H -1.012510 0.068042 -0.842559
H -3.559050 0.518404 -2.631802
H -2.512304 -0.897961 -2.766375
H -2.828184 0.105151 -4.187000
H 0.014473 -0.550340 -3.072879
H -0.423725 0.303385 -4.541605
H 1.207494 1.615110 -2.331885
H 2.100105 -0.127773 -4.477705
H 2.799712 2.010236 -4.612098

1b-C48, ΔG = 4.2614 kcal/mol, population = 0.05 %
C 5.463267 3.583139 -1.993076
C 5.162097 2.445025 -1.247850
C 4.886467 1.244412 -1.890063
C 4.899631 1.159071 -3.285771
C 5.216068 2.300405 -4.020216
C 5.494966 3.505415 -3.380484
C 4.557944 -0.141409 -3.970978
C 3.092885 -0.576590 -3.749948
C 2.868063 -2.005653 -4.232058
O 3.709295 -2.855805 -3.649448
O 2.011056 -2.327290 -5.026072
C 3.582717 -4.265130 -3.999987
C 4.605596 -5.024926 -3.191215
C -0.240790 0.695478 -2.150826
C 0.494196 1.102896 -0.871557
C -0.174044 -0.818828 -2.368221
C 0.197167 1.533572 -3.364847
C 1.688627 1.509034 -3.737698
N 2.125428 0.285468 -4.424411
C 1.994363 2.715335 -4.645827
O 1.797986 3.865822 -4.325103
O 2.484117 2.379257 -5.838584
H 5.674343 4.520401 -1.494018
H 5.143508 2.493461 -0.166247
H 4.655771 0.364789 -1.300089
H 5.232144 2.251076 -5.101494
H 5.730488 4.383395 -3.968749
H 4.738289 -0.063224 -5.044222
H 5.199662 -0.934073 -3.585692
H 2.915012 -0.601882 -2.668151
H 2.563930 -4.581521 -3.776851

H 3.749851 -4.364767 -5.072391
H 4.538472 -6.086969 -3.432484
H 5.616288 -4.684532 -3.419623
H 4.426401 -4.904374 -2.122126
H -1.297480 0.947603 -2.011626
H 0.439819 2.181509 -0.708355
H 0.055894 0.606798 -0.003344
H 1.550231 0.823515 -0.903934
H -0.699437 -1.338472 -1.564412
H 0.852459 -1.186214 -2.364279
H -0.636288 -1.111201 -3.313449
H -0.382475 1.234280 -4.244416
H -0.060498 2.574451 -3.163454
H 2.290484 1.663160 -2.842189
H 1.338583 -0.273792 -4.732241
H 2.563117 1.385257 -5.781733

1b-C44, ΔG = 4.2984 kcal/mol, population = 0.04 %
C 4.916048 3.912071 -1.982031
C 5.003968 3.787460 -3.364368
C 5.097543 2.526763 -3.947479
C 5.097262 1.374983 -3.161430
C 5.026404 1.512710 -1.772256
C 4.934307 2.769153 -1.185834
C 5.109370 0.005936 -3.789960
C 3.732335 -0.687044 -3.735823
C 3.875065 -2.138489 -4.188098
O 2.870763 -2.536747 -4.974347
O 4.789422 -2.849524 -3.838986
C 2.851252 -3.928897 -5.410494
C 2.200645 -4.812996 -4.368352
C 0.073171 1.300676 -1.939216
C 1.050999 1.517849 -0.781524
C -1.205655 0.620565 -1.443959
C 0.695713 0.460766 -3.064256
C 1.945542 1.041933 -3.740028
N 2.712137 0.038124 -4.500225
C 1.640236 2.219405 -4.674199
O 0.908961 3.142337 -4.394924
O 2.281892 2.157706 -5.844382
H 4.838113 4.891169 -1.526770
H 4.995796 4.670400 -3.991054
H 5.157419 2.436698 -5.024978
H 5.030583 0.627588 -1.146608

H 4.873310 2.857540 -0.108389
H 5.414861 0.074751 -4.835343
H 5.823181 -0.637311 -3.275608
H 3.431966 -0.769658 -2.681816
H 2.278578 -3.904483 -6.334276
H 3.873202 -4.235682 -5.624127
H 2.135096 -5.832539 -4.752885
H 2.783844 -4.831978 -3.447774
H 1.191340 -4.466688 -4.142400
H -0.194040 2.275150 -2.352164
H 1.932140 2.087325 -1.081125
H 0.568127 2.067388 0.029132
H 1.393462 0.559666 -0.378651
H -0.985151 -0.367307 -1.028160
H -1.684402 1.212687 -0.661127
H -1.925060 0.489464 -2.255557
H 0.983356 -0.508840 -2.643742
H -0.057278 0.257114 -3.831512
H 2.606292 1.455198 -2.979771
H 2.073298 -0.632466 -4.913376
H 2.801102 1.309629 -5.787380

1b-C34, ΔG = 4.3706 kcal/mol, population = 0.04 %
C 6.958875 1.975484 -4.570049
C 7.057017 0.805859 -3.821647
C 6.111718 0.520303 -2.842403
C 5.047731 1.392965 -2.598205
C 4.970865 2.570887 -3.343159
C 5.914933 2.861624 -4.323051
C 3.988973 1.058317 -1.579807
C 2.897247 0.099204 -2.112932
C 3.502744 -1.267468 -2.410839
O 3.275956 -1.679054 -3.659578
O 4.107280 -1.905626 -1.578772
C 3.802591 -2.978478 -4.063008
C 5.236666 -2.859653 -4.532256
C -0.685825 -0.303987 -3.652768
C -0.804044 0.198164 -5.093703
C -1.999822 -0.942641 -3.194791
C -0.279834 0.792442 -2.657958
C 1.045357 1.517821 -2.941104
N 2.188398 0.659287 -3.264712
C 0.925736 2.607956 -4.017537
O -0.010625 3.366745 -4.110901

O 1.988179 2.690819 -4.823648
H 7.694702 2.198331 -5.332406
H 7.872695 0.115563 -3.997647
H 6.202830 -0.386821 -2.258918
H 4.168575 3.272156 -3.150289
H 5.835229 3.780204 -4.891016
H 4.432760 0.591752 -0.699987
H 3.494432 1.970685 -1.243953
H 2.206544 -0.092066 -1.282347
H 3.704033 -3.664759 -3.224267
H 3.139749 -3.289554 -4.866648
H 5.896147 -2.564437 -3.717200
H 5.570498 -3.827600 -4.910760
H 5.321697 -2.128024 -5.336429
H 0.077497 -1.090338 -3.628438
H 0.143858 0.570389 -5.487673
H -1.128724 -0.610202 -5.751718
H -1.533882 1.007793 -5.164137
H -1.918257 -1.329739 -2.176739
H -2.279942 -1.769754 -3.850108
H -2.811432 -0.210015 -3.212269
H -1.066015 1.549028 -2.604998
H -0.211167 0.346042 -1.663038
H 1.304988 2.083854 -2.041648
H 1.907581 -0.091495 -3.883228
H 2.596303 1.968956 -4.513251

1b-C22, ΔG = 4.4252 kcal/mol, population = 0.03 %
C 5.386048 3.691797 -1.989791
C 5.126656 2.532823 -1.261019
C 4.911546 1.329139 -1.920155
C 4.942493 1.260998 -3.316462
C 5.215154 2.423936 -4.034341
C 5.435219 3.632153 -3.377566
C 4.665713 -0.045133 -4.019545
C 3.228882 -0.560700 -3.792322
C 3.066488 -1.993139 -4.290088
O 3.980189 -2.801288 -3.756369
O 2.194293 -2.347413 -5.052865
C 3.901137 -4.222057 -4.078895
C 2.922615 -4.929735 -3.165884
C -0.127881 0.525736 -2.117173
C 0.632643 0.943130 -0.856199
C -0.012263 -0.982639 -2.356069

C 0.240486 1.394039 -3.333505
C 1.723462 1.452525 -3.734020
N 2.210547 0.260563 -4.442397
C 1.951737 2.682554 -4.632648
O 2.445290 2.385679 -5.834192
O 1.697815 3.817512 -4.297018
H 5.550673 4.631417 -1.477879
H 5.094059 2.567364 -0.179233
H 4.713749 0.433159 -1.343006
H 5.243706 2.388601 -5.116035
H 5.637968 4.526597 -3.953093
H 5.352899 -0.808023 -3.652993
H 4.831698 0.060424 -5.092678
H 3.062883 -0.612609 -2.709086
H 3.625958 -4.324127 -5.126859
H 4.917915 -4.579182 -3.935333
H 2.930810 -5.998296 -3.389057
H 3.202632 -4.796176 -2.120194
H 1.908465 -4.557733 -3.311450
H -1.188201 0.736117 -1.941409
H 1.695080 0.696594 -0.925205
H 0.550413 2.017612 -0.678038
H 0.237464 0.423624 0.018939
H -0.500052 -1.281271 -3.286242
H -0.485873 -1.530816 -1.539063
H 1.026336 -1.311427 -2.397082
H -0.339625 1.069450 -4.203858
H -0.068056 2.418366 -3.118890
H 2.331647 1.627811 -2.847022
H 1.446913 -0.333396 -4.743912
H 2.575782 1.396439 -5.789752

1b-C27, ΔG = 4.4879 kcal/mol, population = 0.03 %
C 5.605432 3.892962 -2.602319
C 5.659125 2.880992 -1.646129
C 5.447668 1.558873 -2.018110
C 5.176888 1.224466 -3.348285
C 5.142981 2.243259 -4.299094
C 5.351676 3.569614 -3.930628
C 4.869400 -0.201741 -3.725008
C 3.453675 -0.629323 -3.277776
C 3.213814 -2.105047 -3.572954
O 4.145349 -2.872696 -3.010877
O 2.278638 -2.519412 -4.223503

C 4.065522 -4.312642 -3.231197
C 4.732735 -4.698333 -4.534332
C -0.692756 0.930153 -2.911590
C -1.741273 0.695334 -1.820495
C -0.791658 -0.158942 -3.984356
C 0.693078 1.048475 -2.257728
C 1.900446 1.312900 -3.174243
N 2.400928 0.152342 -3.927265
C 1.650129 2.470378 -4.148636
O 2.013322 2.209804 -5.406303
O 1.181305 3.536473 -3.820366
H 5.764573 4.924012 -2.312988
H 5.865147 3.123002 -0.610915
H 5.485819 0.777798 -1.267271
H 4.942204 1.999199 -5.334617
H 5.312650 4.348615 -4.681665
H 4.939634 -0.329063 -4.806564
H 5.589164 -0.875709 -3.260804
H 3.396355 -0.532402 -2.186784
H 4.581825 -4.736651 -2.373489
H 3.018656 -4.609208 -3.207187
H 4.716817 -5.784817 -4.639076
H 4.210754 -4.265255 -5.387677
H 5.772102 -4.367896 -4.549633
H -0.916949 1.889873 -3.386684
H -2.747453 0.679400 -2.244178
H -1.572967 -0.263601 -1.321620
H -1.706251 1.479324 -1.060914
H -0.190793 0.067903 -4.867608
H -1.823526 -0.257878 -4.327125
H -0.479215 -1.131218 -3.593011
H 0.651720 1.859218 -1.528091
H 0.910655 0.135885 -1.693052
H 2.717138 1.666744 -2.543752
H 1.646408 -0.480106 -4.167407
H 2.395794 1.291982 -5.358022

1b-C26, ΔG = 4.5582 kcal/mol, population = 0.03 %
C 5.709513 3.684228 -2.294007
C 5.619366 3.517883 -3.670892
C 5.279437 2.277391 -4.204297
C 5.024058 1.188065 -3.373582
C 5.132250 1.362822 -1.990308
C 5.468194 2.599361 -1.453603

C 4.636334 -0.153617 -3.945789
C 3.215366 -0.605098 -3.545281
C 2.974368 -2.059497 -3.938312
O 3.907336 -2.862711 -3.431091
O 2.033132 -2.431313 -4.604696
C 3.818430 -4.286597 -3.736320
C 4.465102 -4.595981 -5.069762
C -0.040452 0.735665 -1.705423
C 0.116091 -0.784610 -1.789486
C -1.467706 1.092192 -1.277493
C 0.290171 1.473641 -3.011404
C 1.758676 1.444448 -3.466445
N 2.157044 0.203609 -4.145863
C 2.002519 2.626640 -4.424499
O 2.367921 2.256598 -5.651155
O 1.854782 3.786521 -4.111888
H 5.968454 4.649012 -1.876522
H 5.807194 4.353782 -4.332991
H 5.199687 2.159023 -5.277377
H 4.951094 0.525280 -1.326401
H 5.544403 2.717218 -0.379837
H 4.700513 -0.131991 -5.034812
H 5.334540 -0.912382 -3.591798
H 3.156005 -0.581718 -2.450161
H 4.345172 -4.762630 -2.912982
H 2.770711 -4.580443 -3.714623
H 5.505188 -4.267703 -5.080850
H 4.444131 -5.674344 -5.238180
H 3.932025 -4.112062 -5.888162
H 0.645802 1.108365 -0.936377
H -0.237600 -1.252926 -0.868674
H -0.470789 -1.193229 -2.616756
H 1.151556 -1.090662 -1.925500
H -2.191338 0.745162 -2.020842
H -1.592326 2.171346 -1.165830
H -1.717127 0.621251 -0.324536
H -0.337750 1.089829 -3.824190
H 0.019230 2.522117 -2.880841
H 2.402787 1.622136 -2.603660
H 1.359447 -0.388497 -4.345127
H 2.444158 1.263625 -5.577131

1b-C25, ΔG = 4.5796 kcal/mol, population = 0.03 %
C 5.654688 3.690066 -2.161754

C 5.556916 3.653710 -3.547631
C 5.252517 2.459585 -4.196210
C 5.039497 1.287724 -3.472632
C 5.155063 1.332610 -2.079831
C 5.455962 2.522039 -1.428336
C 4.689598 -0.005027 -4.168239
C 3.294351 -0.547174 -3.791394
C 3.096710 -1.969572 -4.305544
O 4.074463 -2.776142 -3.897883
O 2.150368 -2.318749 -4.976920
C 3.980207 -4.189056 -4.249712
C 3.115310 -4.936746 -3.257369
C 0.055948 0.494064 -1.771875
C 0.214332 -1.007563 -2.025753
C -1.352642 0.787129 -1.245050
C 0.322815 1.371481 -3.004190
C 1.775148 1.433875 -3.503497
N 2.196641 0.272617 -4.299530
C 1.951116 2.702818 -4.360224
O 2.304807 2.454319 -5.620552
O 1.765355 3.824841 -3.946140
H 5.886136 4.618389 -1.655049
H 5.711233 4.555101 -4.127349
H 5.167824 2.442328 -5.275382
H 5.006209 0.429814 -1.498699
H 5.538150 2.538385 -0.348705
H 4.729969 0.126764 -5.250601
H 5.422233 -0.767715 -3.903046
H 3.251988 -0.624945 -2.697683
H 3.595337 -4.267963 -5.264607
H 5.011241 -4.533092 -4.226120
H 3.113128 -5.998587 -3.510623
H 3.504890 -4.827125 -2.244497
H 2.086695 -4.577080 -3.282395
H 0.773255 0.788543 -0.997401
H -0.095156 -1.574702 -1.145493
H -0.410081 -1.328729 -2.864036
H 1.242150 -1.292182 -2.242037
H -1.557606 0.210708 -0.340702
H -2.107160 0.518571 -1.990181
H -1.476516 1.845834 -1.008103
H -0.322966 1.055103 -3.831817
H 0.031114 2.393391 -2.757992
H 2.438808 1.558992 -2.646401

H 1.413921 -0.327113 -4.532354
H 2.418338 1.462141 -5.634545

1b-C37, ΔG = 4.6781 kcal/mol, population = 0.02 %
C 5.465610 4.219353 -2.654140
C 5.242559 3.894363 -3.987607
C 5.182762 2.560057 -4.380743
C 5.338638 1.533946 -3.450019
C 5.575853 1.871881 -2.114393
C 5.637820 3.202240 -1.717576
C 5.191534 0.090414 -3.853896
C 3.830831 -0.496216 -3.418272
C 3.817341 -1.997542 -3.688364
O 2.747213 -2.391315 -4.385469
O 4.686589 -2.740404 -3.293306
C 2.569450 -3.817657 -4.632525
C 1.853291 -4.474395 -3.471985
C -0.001617 -0.281332 -2.721667
C -0.831839 0.240794 -3.896733
C -0.911522 -0.716174 -1.569768
C 1.024826 0.736519 -2.205482
C 2.062277 1.239188 -3.222074
N 2.704554 0.204378 -4.044000
C 1.530990 2.342390 -4.146878
O 0.822815 3.251095 -3.779827
O 1.968046 2.248798 -5.406382
H 5.508930 5.256167 -2.345567
H 5.112304 4.678003 -4.723390
H 5.006255 2.314272 -5.420511
H 5.705061 1.085861 -1.379078
H 5.819795 3.446659 -0.678461
H 5.274761 -0.007888 -4.937478
H 5.977985 -0.512622 -3.399313
H 3.766110 -0.423484 -2.326273
H 1.979700 -3.853944 -5.545099
H 3.546906 -4.260259 -4.812799
H 1.669323 -5.523834 -3.709185
H 2.453661 -4.429538 -2.563228
H 0.893129 -3.991247 -3.286643
H 0.538892 -1.173099 -3.056963
H -0.221997 0.468537 -4.773367
H -1.567699 -0.505065 -4.203473
H -1.366901 1.151902 -3.619605
H -1.496270 0.130096 -1.198717

H -1.610007 -1.489605 -1.895531
H -0.330263 -1.115102 -0.735510
H 0.506598 1.609671 -1.802487
H 1.567492 0.285062 -1.371136
H 2.851145 1.744701 -2.661231
H 2.019512 -0.479848 -4.340578
H 2.534075 1.432215 -5.406696

1b-C24, ΔG = 4.7170 kcal/mol, population = 0.02 %
C 5.413484 3.686650 -2.114322
C 5.121693 2.604588 -1.286423
C 4.870602 1.354028 -1.836706
C 4.898646 1.162243 -3.221760
C 5.206654 2.248352 -4.038843
C 5.461551 3.503052 -3.491283
C 4.574333 -0.189432 -3.808177
C 3.107357 -0.612682 -3.573689
C 2.893573 -2.070677 -3.966830
O 3.732967 -2.876622 -3.319377
O 2.046967 -2.442205 -4.749853
C 3.666548 -4.304511 -3.610912
C 4.494061 -4.645712 -4.831867
C -0.190568 0.760135 -2.046471
C 0.601760 1.225351 -0.822205
C -0.169272 -0.765953 -2.171918
C 0.219232 1.511255 -3.325839
C 1.703065 1.469358 -3.723220
N 2.146316 0.199722 -4.315372
C 1.984855 2.601537 -4.729758
O 2.485649 2.177211 -5.889138
O 1.765293 3.770798 -4.506299
H 5.605085 4.662663 -1.686818
H 5.090568 2.735981 -0.212023
H 4.646448 0.518745 -1.183111
H 5.234295 2.116665 -5.112932
H 5.690827 4.336692 -4.143035
H 5.212990 -0.946379 -3.352767
H 4.770405 -0.194390 -4.881538
H 2.918353 -0.566358 -2.494544
H 4.061465 -4.772574 -2.712631
H 2.622241 -4.584064 -3.736305
H 4.478192 -5.726108 -4.987216
H 4.094097 -4.166202 -5.725364
H 5.530528 -4.334260 -4.696143

H -1.235150 1.046429 -1.884247
H 1.647629 0.912473 -0.873469
H 0.584338 2.313348 -0.726348
H 0.181790 0.798565 0.090668
H -0.675845 -1.102035 -3.079056
H -0.676030 -1.220963 -1.318405
H 0.846287 -1.162499 -2.183833
H -0.376608 1.148563 -4.170156
H -0.038660 2.563057 -3.193078
H 2.316433 1.700166 -2.852923
H 1.362300 -0.379660 -4.592114
H 2.580253 1.192479 -5.750239

1b-C40, ΔG = 5.0615 kcal/mol, population = 0.01 %
C 7.102734 2.485668 -4.155546
C 6.025118 3.288787 -3.797518
C 4.964858 2.752180 -3.072899
C 4.957641 1.407978 -2.698420
C 6.055728 0.615850 -3.047908
C 7.116696 1.147146 -3.772582
C 3.792593 0.827181 -1.937730
C 2.809839 -0.004486 -2.797022
C 3.488074 -1.200254 -3.449185
O 4.045656 -1.995232 -2.536282
O 3.493538 -1.411112 -4.641779
C 4.683662 -3.221352 -3.003700
C 3.666112 -4.329413 -3.173130
C -0.748166 -0.421822 -3.062702
C -0.191326 -1.672490 -3.749379
C -2.268767 -0.545403 -2.922374
C -0.396725 0.880708 -3.807296
C 0.948812 1.522618 -3.441625
N 2.154993 0.786427 -3.837084
C 1.024840 2.943587 -4.034370
O 2.165950 3.202957 -4.671270
O 0.142771 3.765690 -3.929696
H 7.929153 2.900454 -4.718753
H 6.009099 4.334514 -4.078402
H 4.137191 3.389537 -2.788948
H 6.088686 -0.421074 -2.739374
H 7.958156 0.516895 -4.032435
H 4.147013 0.182748 -1.132908
H 3.219432 1.630476 -1.472616
H 2.075270 -0.430952 -2.104393

H 5.208683 -3.006295 -3.932559
H 5.406369 -3.452311 -2.224943
H 3.132016 -4.510157 -2.239491
H 2.944424 -4.084757 -3.952316
H 4.179991 -5.249328 -3.458219
H -0.326276 -0.361602 -2.053137
H -0.429679 -2.568160 -3.172316
H -0.634343 -1.786038 -4.742569
H 0.888717 -1.642910 -3.877734
H -2.540538 -1.468433 -2.405725
H -2.744951 -0.561091 -3.907067
H -2.684314 0.294074 -2.361305
H -0.430848 0.710172 -4.888819
H -1.157825 1.631080 -3.590859
H 0.975674 1.667446 -2.358108
H 1.989120 0.207435 -4.652933
H 2.695897 2.364593 -4.569507

1b-C54, ΔG = 5.0853 kcal/mol, population = 0.01 %
C 5.576722 3.997044 -2.226353
C 5.460851 3.919523 -3.609180
C 5.243808 2.689634 -4.224761
C 5.139582 1.521463 -3.471624
C 5.271499 1.609289 -2.082287
C 5.483381 2.834848 -1.463373
C 4.904072 0.187566 -4.137278
C 3.597551 -0.509391 -3.709706
C 3.650839 -1.980124 -4.121649
O 2.526028 -2.396386 -4.708003
O 4.608028 -2.686306 -3.901182
C 2.410011 -3.810362 -5.047372
C 1.900717 -4.605016 -3.863987
C 0.234301 0.316759 -1.692461
C 0.604648 -1.161869 -1.833166
C -1.221197 0.451834 -1.234051
C 0.434007 1.139459 -2.973583
C 1.881235 1.307294 -3.464533
N 2.392700 0.150812 -4.216351
C 1.953304 2.551788 -4.369079
O 1.691326 3.671821 -3.993349
O 2.303379 2.281560 -5.626433
H 5.740257 4.952854 -1.744863
H 5.532913 4.816166 -4.211918
H 5.143436 2.639217 -5.301541

H 5.208166 0.711841 -1.477904
H 5.579587 2.883809 -0.385875
H 4.895035 0.304966 -5.222120
H 5.723092 -0.488954 -3.889366
H 3.584213 -0.545750 -2.612081
H 1.707213 -3.823750 -5.876704
H 3.381255 -4.163373 -5.387751
H 1.768568 -5.647048 -4.160984
H 2.607927 -4.572925 -3.035119
H 0.938694 -4.220462 -3.523582
H 0.874103 0.756652 -0.919040
H 0.339051 -1.706905 -0.925092
H 0.066446 -1.623800 -2.665587
H 1.670443 -1.313024 -1.994951
H -1.384455 -0.079848 -0.294539
H -1.495475 1.498169 -1.084039
H -1.901239 0.029488 -1.979582
H -0.165660 0.713012 -3.786496
H 0.040748 2.140726 -2.792731
H 2.525252 1.519652 -2.609344
H 1.664954 -0.535006 -4.364225
H 2.482210 1.299670 -5.609364

1b-C47, ΔG = 5.6513 kcal/mol, population = 0.00 %
C 5.229903 3.999842 -2.051848
C 5.260547 3.883100 -3.436499
C 5.158983 2.629928 -4.035178
C 5.025410 1.477887 -3.262082
C 5.013240 1.605379 -1.869520
C 5.109660 2.853931 -1.268282
C 4.882060 0.120229 -3.904065
C 3.515018 -0.545701 -3.643844
C 3.601134 -2.030529 -3.993546
O 2.569348 -2.452530 -4.727882
O 4.501493 -2.740750 -3.608211
C 2.478744 -3.876414 -5.031969
C 1.797633 -4.622709 -3.904830
C 0.067585 0.334162 -2.027679
C 0.801974 0.881006 -0.801169
C 0.294283 -1.172846 -2.181616
C 0.356694 1.157195 -3.296036
C 1.825783 1.312819 -3.717944
N 2.408094 0.113012 -4.339530
C 1.938085 2.482573 -4.713268

O 2.456562 2.137063 -5.891343
O 1.579664 3.612178 -4.467542
H 5.301949 4.973722 -1.584460
H 5.356356 4.766932 -4.054594
H 5.171104 2.548755 -5.114674
H 4.925974 0.720427 -1.249708
H 5.093536 2.933969 -0.188509
H 5.032631 0.193582 -4.982347
H 5.645766 -0.551399 -3.510462
H 3.347767 -0.542765 -2.558994
H 1.897052 -3.910862 -5.949761
H 3.481828 -4.252208 -5.222875
H 1.681137 -5.670834 -4.186441
H 2.388295 -4.578210 -2.989840
H 0.808543 -4.207747 -3.708149
H -1.004385 0.473080 -1.851556
H 0.637044 1.954348 -0.683813
H 0.454085 0.383836 0.106425
H 1.880037 0.714836 -0.866590
H 1.353511 -1.428887 -2.212250
H -0.135047 -1.706743 -1.331447
H -0.174185 -1.559990 -3.088948
H -0.202255 0.737203 -4.138958
H -0.031988 2.164139 -3.137028
H 2.421600 1.609561 -2.855148
H 1.689618 -0.564253 -4.557404
H 2.667678 1.167817 -5.773145

1b-C51, ΔG = 5.6865 kcal/mol, population = 0.00 %
C 5.255150 4.027748 -2.177784
C 5.315611 3.791086 -3.546108
C 5.184395 2.495118 -4.038218
C 4.990191 1.419281 -3.173664
C 4.947839 1.666510 -1.797935
C 5.074838 2.958384 -1.302880
C 4.818485 0.016869 -3.701664
C 3.430347 -0.590339 -3.417500
C 3.481617 -2.100729 -3.648279
O 2.414074 -2.560687 -4.304378
O 4.380601 -2.796611 -3.234834
C 2.323104 -3.995970 -4.548892
C 3.063423 -4.376521 -5.812872
C 0.000924 0.568217 -1.948809
C 0.740936 1.205339 -0.770249

C 0.175502 -0.953364 -1.953403
C 0.333589 1.253599 -3.286619
C 1.812358 1.313482 -3.698987
N 2.355230 0.036089 -4.189382
C 1.982854 2.372239 -4.804224
O 2.501084 1.890181 -5.933601
O 1.668012 3.534017 -4.677913
H 5.351558 5.035273 -1.793531
H 5.458520 4.614653 -4.234372
H 5.221280 2.320638 -5.106102
H 4.812058 0.841785 -1.107727
H 5.035257 3.131524 -0.234706
H 5.555632 -0.639006 -3.237357
H 4.993063 -0.005324 -4.778639
H 3.240131 -0.496286 -2.340104
H 2.711228 -4.518132 -3.676627
H 1.254692 -4.177582 -4.635391
H 2.922201 -5.441803 -6.004883
H 2.679404 -3.820363 -6.668945
H 4.131924 -4.184932 -5.715655
H -1.067279 0.760611 -1.802555
H 1.812282 0.993034 -0.801955
H 0.616264 2.290462 -0.760717
H 0.362916 0.813247 0.175958
H -0.300455 -1.410474 -2.823641
H -0.277907 -1.386007 -1.059264
H 1.225494 -1.246735 -1.949436
H -0.231229 0.773508 -4.092873
H -0.019426 2.284401 -3.232060
H 2.408673 1.673223 -2.861221
H 1.613645 -0.632935 -4.347088
H 2.671588 0.930203 -5.715385

1b-C55, ΔG = 5.8609 kcal/mol, population = 0.00 %
C 5.426730 4.200036 -2.752396
C 5.138832 3.832813 -4.062143
C 5.047977 2.486806 -4.406948
C 5.236773 1.490573 -3.450299
C 5.540059 1.870827 -2.139514
C 5.633083 3.212803 -1.791112
C 5.068438 0.034855 -3.799183
C 3.753334 -0.559380 -3.254717
C 3.761794 -2.073707 -3.454562
O 2.587870 -2.544247 -3.886192

O 4.724802 -2.764451 -3.212162
C 2.459771 -3.979747 -4.109694
C 2.937336 -4.353495 -5.496466
C -0.505245 0.585082 -2.631233
C -1.440071 0.269956 -1.460097
C -0.563411 -0.536425 -3.673671
C 0.903986 0.869603 -2.086931
C 2.009837 1.212392 -3.100886
N 2.566878 0.069417 -3.844640
C 1.574678 2.292731 -4.097545
O 1.025174 3.321888 -3.776923
O 1.871218 2.010294 -5.368171
H 5.495221 5.246061 -2.481808
H 4.981959 4.592703 -4.817405
H 4.818908 2.209290 -5.428193
H 5.698080 1.108863 -1.384725
H 5.866550 3.489459 -0.770510
H 5.078533 -0.095756 -4.882525
H 5.891457 -0.547642 -3.384998
H 3.748678 -0.435262 -2.163501
H 3.016979 -4.499896 -3.333141
H 1.396679 -4.169112 -3.983340
H 4.002489 -4.153607 -5.612836
H 2.769095 -5.419675 -5.659535
H 2.386931 -3.799327 -6.257772
H -0.866276 1.501843 -3.106927
H -2.466477 0.135010 -1.807335
H -1.132826 -0.651216 -0.956658
H -1.435651 1.074099 -0.721135
H -0.123740 -1.460993 -3.288640
H -0.053251 -0.270045 -4.601740
H -1.600644 -0.750844 -3.938409
H 0.831057 1.702522 -1.385417
H 1.254252 0.008622 -1.508145
H 2.828040 1.673021 -2.546116
H 1.858374 -0.639565 -3.975808
H 2.342783 1.135115 -5.314075

1b-C38, ΔG = 5.9494 kcal/mol, population = 0.00 %
C 7.165993 2.242911 -4.454221
C 7.132262 0.923888 -4.009687
C 6.098335 0.489036 -3.188398
C 5.074105 1.359031 -2.801400
C 5.131217 2.683666 -3.237140

C 6.165129 3.123848 -4.058950
C 3.934674 0.874644 -1.941013
C 2.842328 0.081542 -2.703567
C 3.418486 -1.148732 -3.391229
O 3.946648 -1.992906 -2.505489
O 3.388125 -1.334109 -4.587283
C 4.500614 -3.245121 -3.009801
C 3.414709 -4.286850 -3.176153
C -0.977796 -0.397152 -3.597464
C -1.508640 -0.330328 -2.163340
C -0.055489 -1.603887 -3.784343
C -0.377932 0.943893 -4.054844
C 0.845445 1.472598 -3.282937
N 2.124969 0.881057 -3.693779
C 0.920897 3.000570 -3.456132
O 2.050957 3.423597 -4.020682
O 0.039712 3.759525 -3.120633
H 7.971140 2.582665 -5.093400
H 7.915791 0.233903 -4.297340
H 6.095211 -0.533753 -2.833719
H 4.367988 3.383135 -2.921116
H 6.188748 4.155976 -4.385949
H 3.441878 1.721079 -1.460599
H 4.310350 0.229402 -1.146062
H 2.150941 -0.296492 -1.943417
H 5.016794 -3.043340 -3.946561
H 5.225096 -3.533892 -2.252318
H 3.865710 -5.229768 -3.490933
H 2.892159 -4.454382 -2.233568
H 2.691331 -3.983220 -3.932682
H -1.840075 -0.549669 -4.255385
H -0.697884 -0.225723 -1.437609
H -2.188643 0.513841 -2.030069
H -2.050052 -1.244552 -1.912074
H 0.397458 -1.620600 -4.777174
H -0.617517 -2.531084 -3.655751
H 0.746137 -1.617511 -3.044696
H -0.108323 0.883671 -5.114328
H -1.161017 1.699457 -3.977022
H 0.697244 1.313810 -2.212505
H 2.037478 0.352544 -4.554018
H 2.582713 2.588504 -4.142924

1b-C35, ΔG = 6.0222 kcal/mol, population = 0.00 %

C 6.926292 2.471813 -3.550789
C 7.002092 1.086498 -3.435308
C 5.927629 0.368023 -2.922270
C 4.757004 1.016780 -2.522540
C 4.699136 2.407088 -2.629620
C 5.771952 3.131200 -3.140653
C 3.574624 0.237233 -2.007637
C 2.719859 -0.412794 -3.118872
C 3.448453 -1.519848 -3.870358
O 3.947407 -2.431118 -3.034296
O 3.512393 -1.587534 -5.077921
C 4.585291 -3.609159 -3.613612
C 6.043980 -3.355181 -3.928796
C -1.272801 0.461567 -2.763860
C -1.739463 1.841841 -2.296821
C -0.697075 -0.347745 -1.598975
C -0.306832 0.530941 -3.960935
C 1.018083 1.288525 -3.717534
N 2.240153 0.566467 -4.088710
C 1.059860 2.659388 -4.411245
O 0.102340 3.383273 -4.549687
O 2.279221 3.013731 -4.828191
H 7.762410 3.032510 -3.949192
H 7.900571 0.564321 -3.740115
H 6.003660 -0.705906 -2.821445
H 3.807983 2.930701 -2.305885
H 5.705325 4.209344 -3.216802
H 3.896111 -0.552118 -1.328633
H 2.924079 0.899717 -1.434628
H 1.885494 -0.924073 -2.621942
H 4.468290 -4.370507 -2.846255
H 4.026429 -3.900682 -4.500856
H 6.490133 -4.274485 -4.312874
H 6.156825 -2.579376 -4.685725
H 6.592647 -3.062033 -3.032859
H -2.153472 -0.077472 -3.128460
H -0.909759 2.431137 -1.898176
H -2.179655 2.409901 -3.116744
H -2.483685 1.743952 -1.503412
H 0.171303 0.144127 -1.151866
H -1.439907 -0.462731 -0.807096
H -0.392670 -1.346839 -1.919396
H -0.053895 -0.490461 -4.255230
H -0.825988 0.983795 -4.806922

H 1.103226 1.532629 -2.655016
H 2.126389 0.106990 -4.987397
H 2.852775 2.228030 -4.625728

1b-C39, ΔG = 6.2437 kcal/mol, population = 0.00 %
C 5.523375 4.000898 -2.573435
C 5.266613 3.739861 -3.914964
C 5.095833 2.428969 -4.350896
C 5.170652 1.363335 -3.455070
C 5.444487 1.635436 -2.111449
C 5.618492 2.942458 -1.672336
C 4.897265 -0.046690 -3.907276
C 3.486587 -0.531259 -3.492293
C 3.286911 -1.971131 -3.941623
O 4.138356 -2.791484 -3.328242
O 2.462124 -2.318469 -4.758676
C 4.057337 -4.213616 -3.645321
C 2.988595 -4.894110 -2.816524
C -0.428660 0.255135 -2.584239
C -0.242258 -1.257124 -2.767165
C -1.116639 0.876650 -3.801892
C 0.854267 0.994626 -2.147111
C 1.911026 1.369150 -3.198556
N 2.425164 0.298921 -4.062817
C 1.503621 2.559406 -4.079327
O 1.890666 2.447478 -5.353161
O 0.932345 3.542493 -3.667326
H 5.653362 5.019853 -2.231576
H 5.196112 4.555595 -4.623472
H 4.893241 2.233020 -5.396330
H 5.512370 0.817499 -1.403238
H 5.826933 3.136432 -0.627591
H 5.628986 -0.730270 -3.475794
H 4.973891 -0.112625 -4.993880
H 3.434585 -0.539221 -2.398462
H 3.872787 -4.320232 -4.712612
H 5.049947 -4.590031 -3.410686
H 2.996823 -5.965017 -3.028249
H 3.177384 -4.754526 -1.751325
H 1.998572 -4.505721 -3.054942
H -1.111175 0.375631 -1.736749
H 0.273231 -1.514450 -3.693180
H 0.326474 -1.688995 -1.940594
H -1.214969 -1.752814 -2.799177

H -1.320871 1.936966 -3.650412
H -2.064831 0.371905 -3.997699
H -0.511482 0.778837 -4.707784
H 0.570188 1.932064 -1.665313
H 1.350188 0.396052 -1.378764
H 2.765851 1.763975 -2.644757
H 1.680325 -0.304025 -4.390873
H 2.355324 1.569742 -5.388362

1b-C42, ΔG = 7.0112 kcal/mol, population = 0.00 %
C 6.767721 2.296834 -4.472633
C 5.750048 3.082614 -3.940813
C 4.830455 2.527872 -3.056154
C 4.907043 1.183068 -2.691111
C 5.944079 0.409446 -3.217340
C 6.865274 0.958340 -4.102809
C 3.876654 0.575300 -1.775682
C 2.751972 -0.182766 -2.519530
C 3.257621 -1.424851 -3.242449
O 3.909923 -2.240927 -2.414417
O 3.045662 -1.659008 -4.411786
C 4.369258 -3.520859 -2.945460
C 5.720375 -3.394828 -3.616370
C -1.061332 0.100665 -3.635815
C -1.102522 0.719228 -5.035559
C -0.567905 -1.352081 -3.683108
C -0.329539 0.971539 -2.592635
C 1.044447 1.564165 -2.949657
N 2.062209 0.661360 -3.489226
C 0.955865 2.796018 -3.866446
O 1.977781 2.908949 -4.719968
O 0.084244 3.630816 -3.799481
H 7.483686 2.725628 -5.162385
H 5.670543 4.127437 -4.213979
H 4.046832 3.149958 -2.641373
H 6.036752 -0.627620 -2.924954
H 7.661614 0.341491 -4.500508
H 4.340430 -0.122017 -1.078509
H 3.409863 1.356591 -1.174396
H 2.062448 -0.562177 -1.753780
H 4.415482 -4.162712 -2.069121
H 3.612964 -3.898211 -3.631071
H 6.469327 -3.015026 -2.920311
H 6.042552 -4.381512 -3.954515

H 5.672269 -2.736095 -4.483049
H -2.094989 0.056502 -3.277975
H -0.107679 0.787492 -5.485121
H -1.709669 0.104402 -5.702827
H -1.528005 1.723023 -5.017167
H -0.495715 -1.776747 -2.679430
H 0.407572 -1.456548 -4.160376
H -1.266468 -1.965892 -4.255937
H -0.964523 1.820782 -2.334652
H -0.216672 0.387271 -1.676203
H 1.448491 1.984093 -2.023099
H 1.684765 0.076761 -4.224939
H 2.542885 2.111311 -4.545980
